# The Study to Understand the Genetics of the Acute Response to Metformin and Glipizide in Humans (SUGAR-MGH): Design of a pharmacogenetic Resource for Type 2 Diabetes

**DOI:** 10.1371/journal.pone.0121553

**Published:** 2015-03-26

**Authors:** Geoffrey A. Walford, Natalia Colomo, Jennifer N. Todd, Liana K. Billings, Marlene Fernandez, Bindu Chamarthi, A. Sofia Warner, Jaclyn Davis, Katherine R. Littleton, Alicia M. Hernandez, Rebecca R. Fanelli, Amelia Lanier, Corinne Barbato, Rachel J. Ackerman, Sabina Q. Khan, Rosa Bui, Laurel Garber, Elliot S. Stolerman, Allan F. Moore, Chunmei Huang, Varinderpal Kaur, Maegan Harden, Andrew Taylor, Ling Chen, Alisa K. Manning, Paul Huang, Deborah Wexler, Rita M. McCarthy, Janet Lo, Melissa K. Thomas, Richard W. Grant, Allison Goldfine, Margo S. Hudson, Jose C. Florez

**Affiliations:** 1 Center for Human Genetic Research, Massachusetts General Hospital, Boston, Massachusetts, United States of America; 2 Diabetes Research Center, Diabetes Unit, Department of Medicine, Massachusetts General Hospital, Boston, Massachusetts, United States of America; 3 Harvard Medical School, Boston, Massachusetts, United States of America; 4 Department of Endocrinology and Nutrition. Hospital Universitario Regional de Málaga. Instituto de Investigación Biomédica de Málaga (IBIMA). Málaga, Spain; 5 Boston Children’s Hospital, Boston, Massachusetts, United States of America; 6 Division of Endocrinology and Metabolism, NorthShore University Health System, Evanston, Illinois, United States of America; 7 Department of Medicine, Brigham and Women’s Hospital, Boston, Massachusetts, United States of America; 8 Division of Endocrinology, Diabetes, and Hypertension, Brigham and Women’s Hospital, Boston, Massachusetts, United States of America; 9 Joslin Diabetes Center, Boston, Massachusetts, United States of America; 10 Genomics Platform, Broad Institute, Cambridge, Massachusetts, United States of America; 11 Division of Research, Kaiser Permanente Northern California, Oakland, California, United States of America; Uppsala University, SWEDEN

## Abstract

**Objective:**

Genome-wide association studies have uncovered a large number of genetic variants associated with type 2 diabetes or related phenotypes. In many cases the causal gene or polymorphism has not been identified, and its impact on response to anti-hyperglycemic medications is unknown. The Study to Understand the Genetics of the Acute Response to Metformin and Glipizide in Humans (SUGAR-MGH, *NCT01762046*) is a novel resource of genetic and biochemical data following glipizide and metformin administration. We describe recruitment, enrollment, and phenotyping procedures and preliminary results for the first 668 of our planned 1,000 participants enriched for individuals at risk of requiring anti-diabetic therapy in the future.

**Methods:**

All individuals are challenged with 5 mg glipizide × 1; twice daily 500 mg metformin × 2 days; and 75-g oral glucose tolerance test following metformin. Genetic variants associated with glycemic traits and blood glucose, insulin, and other hormones at baseline and following each intervention are measured.

**Results:**

Approximately 50% of the cohort is female and 30% belong to an ethnic minority group. Following glipizide administration, peak insulin occurred at 60 minutes and trough glucose at 120 minutes. Thirty percent of participants experienced non-severe symptomatic hypoglycemia and required rescue with oral glucose. Following metformin administration, fasting glucose and insulin were reduced. Common genetic variants were associated with fasting glucose levels.

**Conclusions:**

SUGAR-MGH represents a viable pharmacogenetic resource which, when completed, will serve to characterize genetic influences on pharmacological perturbations, and help establish the functional relevance of newly discovered genetic loci to therapy of type 2 diabetes.

**Trial Registration:**

ClinicalTrials.gov NCT01762046

## Introduction

In recent years we have witnessed an explosion of genetic discovery, driven by the development of high-throughput genotyping and sequencing techniques, the implementation of rigorous and novel analytical methods, and widespread international collaboration [1, 2]. In type 2 diabetes, there are now over 60 loci associated with the disease at genome-wide levels of statistical significance [3, 4]; similar progress has been made for type 2 diabetes-related quantitative traits [5].

However, in spite of substantial advances in the mapping of genomic regions whose variation contributes to type 2 diabetes pathogenesis, both the elucidation of functional mechanism and their clinical translation lag behind. In most cases the precise nucleotide variant or gene whose alteration gives rise to the phenotype have not been identified, hindering the rapid generation of animal or cellular experimental models, the validation of drug targets, and the development of gene-based therapeutics. Beyond the absence of clear molecular mechanisms, the ability of type 2 diabetes-associated genetic markers to improve disease prediction over common clinical variables is limited [6–8], and the selection of therapeutic approaches for patients with type 2 diabetes remains algorithmic despite recent attempts at greater individualization [9, 10].

Pharmacogenetic studies offer an opportunity to address both scientific shortcomings [11]. The robust association of a genomic region with drug response (regardless of whether the tested variant tags the causal nucleotide or is itself causative) can help tailor therapy based on genetic determinants relevant to a specific agent; similarly, differential perturbation of the human organism with a medication that has known physiological effects *in vivo*, contingent on the allele at a specific locus, can help implicate a gene associated with type 2 diabetes through an agnostic genomic search but for which a clear mechanism of action was lacking.

We therefore designed a study that might serve multiple purposes. The Study to Understand the Genetics of the Acute Response to Metformin and Glipizide in Humans (SUGAR-MGH) employs two pharmacological interventions (glipizide and metformin) chosen to perturb two different limbs of the glucose homeostatic system (insulin secretion and insulin action), under basal and hyperglycemic conditions; it collects physiological, hormonal, metabolomic and genetic measures; and it does so under a relatively simple protocol that allows for the efficient enrollment and retention of a sufficiently large number of participants to support genetic analyses. This paper describes the study protocol, recruitment methods, physiological measurements, and intervention outcomes. We also perform baseline comparisons to guide the selection of primary and secondary phenotype endpoints in the first two thirds of our intended final enrollment of 1,000 participants, and demonstrate the use of genetic risk scores (GRS) based on fasting glucose or insulin.

## Materials and Methods

### Cohort and Study Design

**Fig 1:**
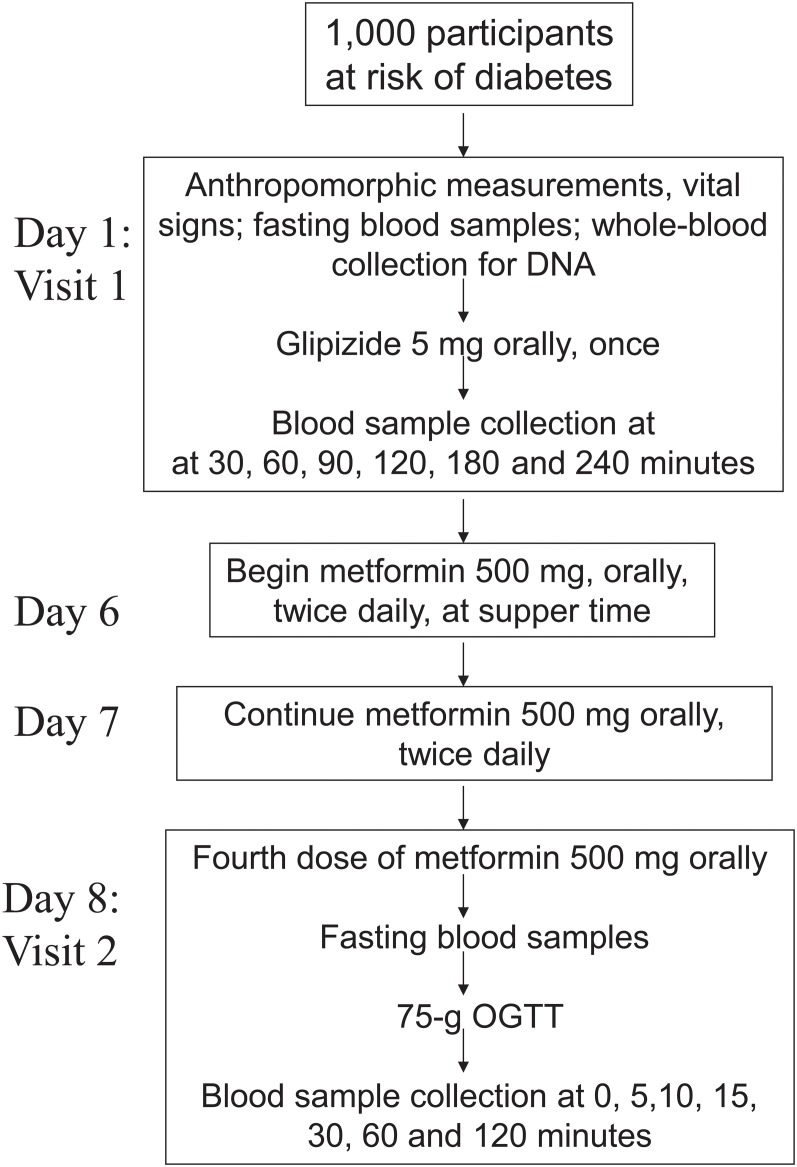
SUGAR-MGH protocol and schedule of events. All events at Visit 1 and Visit 2 occur in the Clinical Research Center.

SUGAR-MGH (*ClinicalTrials*.*Gov*: *NCT01762046*, clinicaltrials.gov/ct2/show/NCT01762046) was established in 2007 with funding from the National Institutes of Health, and a streamlined protocol was designed that requires only two clinical research center (CRC) visits and 1 week of active treatment per subject (Fig. 1). SUGAR-MGH was approved by the local human research committee on April 14, 2007. The TREND Statement Checklist has been uploaded as a supplementary file (S1 TREND Checklist). The first participant was enrolled on February 14, 2008. Recruitment is ongoing, and all subjects are followed until study completion or loss to follow-up. All CRC visits took place at an academic medical center (Massachusetts General Hospital, Brigham and Women’s Hospital, or the Joslin Diabetes Center, all in Boston, MA). Interventions were administered by study staff at the CRC to each participant separately.

Registration in *ClinicalTrials*.*gov* was not immediately sought because the aim of the study, using existing approved drugs to modulate the glycemic homeostasis system and compare acute responses across genotypes, was meant to characterize the physiological impact of genetic changes and not to support new indications for these drugs. Despite the issuance of stricter guidelines effective July 1, 2008, communications from both the program officer at the National Institutes of Health (August 21, 2007) and the local human research committee (September 14, 2011) confirmed that our study did not require registration. However, in the face of continuously evolving guidelines and their potentially equivocal interpretation with respect to SUGAR-MGH, the study team initiated registration in late 2012 and accomplished registration in *ClinicalTrials*.*gov* in January 2013. The authors confirm that all ongoing and related trials for this drug/intervention are registered.

### Ethics Statement

The protocol is approved by the Partners Human Research Committee (Partners Healthcare, Boston, MA). Written informed consent is obtained from all study participants; the original consent form (S1 File) and the original study protocol (S1 Protocol), as well as the most recent and current versions of these documents (S2 File and S2 Protocol, respectively), approved by the human research committee are provided as supplementary files.

#### Visit 1 (Day 1)

After an overnight fast of at least 8 hours, participants receive a single, open-label oral dose of 5 mg glipizide in the CRC and remain resident in the CRC through conclusion of the 240 minute glipizide challenge. Participants with a fasting blood glucose <4.44 mmol/L are not dosed with glipizide. In addition, the period of observation following glipizide administration may be terminated prior to 240 minutes if a participant develops neuroglycopenic symptoms (confusion, blurred vision, slurred speech), a blood glucose ≤2.77 mmol/L with symptoms of hypoglycemia, blood glucose <2.50 mmol/L with or without symptoms of hypoglycemia, or at the discretion of study staff based on clinical assessment. All participants are provided with a meal at the end of the study visit and discharged only when blood glucose is documented to be greater than 4.44 mmol/L. Five days later, an adequate “wash-out” period for glipizide, participants begin a two-day open-label course of 500 mg metformin, orally, twice daily. Participants who are discovered to have contraindications to safe metformin use at Visit 1 screening laboratories are informed not to take the medication. Participants are permitted to take fewer than the four prescribed doses of metformin should they develop side effects consistent with metformin intolerance.

#### Visit 2 (Day 8)

After another overnight fast of at least 8 hours, participants return to the CRC, receive the fourth dose of metformin and one hour later undergo a standard, two-hour, 75-g oral glucose tolerance test (OGTT).

### Rationale for interventions

Glipizide and metformin are generic medications commonly used to treat type 2 diabetes [12]. Metformin and glipizide are considered first and second-line therapy, respectively, for individuals with newly diagnosed diabetes by major professional organizations [9, 13]. Metformin has further been demonstrated to be effective in preventing incident diabetes in at-risk individuals [14, 15]. Yet, clinical response to both therapies is heterogeneous, and many patients with type 2 diabetes treated with either metformin or glipizide eventually require additional therapy [16, 17]. Therefore, understanding and characterizing the role of genetics in the response to both medications has direct clinical relevance. Given their different mechanisms of action, glipizide through increased insulin secretion and metformin through reduced hepatic glucose output, the study of the response to these two medications is hypothesized to reveal distinct influences on glucose homeostasis. A 75-g OGTT is used in clinical practice for the diagnosis of diabetes, and, in SUGAR-MGH, tests the physiological response to oral glucose ingestion in the presence of metformin.

### Participants

Male or non-pregnant female adults, naïve to glipizide and metformin, are eligible for the study. Individuals at high risk of developing type 2 diabetes are preferentially enrolled by targeting for recruitment persons with the metabolic syndrome, obesity, a history of gestational diabetes, a history of polycystic ovary syndrome, or a family history of type 2 diabetes; individuals with lifestyle-controlled type 2 diabetes are also eligible for the study. The protocol excludes individuals who are currently taking medications used to treat diabetes or that are known to affect glycemic parameters, have had onset of diabetes before age 25 with autosomal transmission of diabetes across three generations, a history of liver or kidney disease (including estimated glomerular filtration rate <60 ml/min/1,73m^2^), severe allergic reactions to sulfonamides, have porphyria (which can be exacerbated by glipizide), established coronary artery disease, a history of bariatric surgery, a history of seizures or stroke, planned radiographic studies requiring contrast within one week of study completion, or are enrolled in any other interventional study at the time of screening through completion of the study protocol. These criteria are implemented to exclude conditions that would confound the interpretation of pharmacological responses or that would make the administration of glipizide or metformin potentially unsafe. For example, a history of established coronary artery disease, seizure or stroke is grounds for exclusion given the concern that hypoglycemia or the adrenergic response to it during the glipizide challenge could exacerbate these conditions; the history of bariatric surgery is excluded given the altered metabolism or absorption following this procedure that would not correlate with genetic or other physiological parameters. Initially and until January 2010, only White participants of European descent were eligible for SUGAR-MGH; restriction of participants to a single ethnic group was mandated by the NIH Study Section due to concerns over confounding factors related to ethnicity. With subsequent demonstration of techniques to control for these factors in genetic analyses, subjects of any ethnic background have been enrolled, and additional efforts have been employed to recruit and enroll subjects from non-White ethnic groups, many of which are at increased risk of type 2 diabetes. Eligible participants are identified using electronic databases and local advertising, and the majority of subjects who consent for participation also agree to be re-contacted for future studies. Participants are reimbursed $50 for completion of Visit 1 and $50 for completion of Visit 2.

### Measurements

A medical history is obtained from all participants; and weight, height, blood pressure, and heart rate are measured at each visit to the CRC. DNA is extracted and genotyping is performed using the iPLEX-GOLD assay from Sequenom by allele-specific primer extension of amplified products, with detection by mass spectroscopy [18]. Plasma glucose is measured by hexokinase assay (Roche; Indianapolis, IN). Insulin international units are determined using a radio-immunoassay (Beckman Coulter; Fullerton, CA). Insulin resistance by homeostasis model assessment (HOMA-IR) is calculated as described in [19]. C-peptide is measured by radio-immunoassay (Siemens, KPED1, Erlangen, Germany); glucagon is measured by radioimmunoassay (LINCOplex Kit, HENDO-65K-Rev, St. Charles, MO) and proinsulin by immunochemiluminometric assay, both at Mayo Medical Laboratories. Incretin hormones (glucagon-like peptide-1 [GLP-1] and glucose-dependent insulinotropic polypeptide [GIP]) are measured from blood samples collected in pre-chilled EDTA tubes containing DPP-IV inhibitor (Millipore; Billerica, MA) in a subset of participants. Active GLP-1 (7–36, 7–37) is measured using the GLP-1(Active) ELISA kit (Millipore; Billerica, MA); total GLP-1 is measured using the GLP-1(7–36 and 9–36) ELISA kit from Alpco Immunoassays (Salem, NH); and GIP is measured by using the Human GIP Total ELISA kit from Millipore (Millipore; Billerica, MA). Metabolite profiling has been conducted in subset of participants on plasma as previously described [20–22]; a pilot metabolomic study in this cohort has been published [23]. Additional blood samples are stored at -80°C for future analysis. Participants are given a prospective food log, completed on three week days and one weekend day between the glipizide challenge and OGTT challenge. All of the food records are analyzed using Nutrition Data System for Research 2009.

### CAMP MGH

Sixty-four (64) participants who completed SUGAR-MGH had previously participated in the MGH Cardiology and Metabolic Patient Cohort study (CAMP MGH). These CAMP MGH participants all underwent a 75-g OGTT in the absence of anti-hyperglycemic therapies, in which plasma glucose was measured by hexokinase assay (Abbott; Chicago, IL), and insulin international units were determined using a radio-immunoassay (Roche; Indianapolis, IN). Of these participants, complete data during the CAMP MGH study were available for measures of fasting glucose (n = 64), fasting insulin (n = 51), and glucose at 30 and 120 minutes during the OGTT (n = 64). Data from the CAMP MGH OGTT (without metformin) and SUGAR-MGH OGTT (with metformin) were compared in this subset of participants.

### Statistical Analyses

A sample size of 1000 participants was chosen to permit sufficient statistical power to detect the effect of commonly occurring genetic variation on pharmacological responses. At alpha = 0.05, there will be 89% power to detect a variant in 10% of the population that explains 1% of the variance in a phenotype. There will be 99% power to detect the same variant if it explains 5% or more of the variance in a phenotype.

Study data are collected and managed using Research Electronic Data Capture (REDCap) electronic data capture tools hosted by Partners HealthCare Research Computing, Enterprise Research Infrastructure & Services group [24]. Area under the curve (AUC) for increases in insulin during the glipizide challenge and for increases in glucose and insulin during the OGTT challenge was calculated by the trapezoidal method and adjusted for baseline values. Area over the curve for decreases in glucose during the glipizide challenge was calculated by subtracting AUC by the trapezoidal method from the baseline glucose value at Visit 1 × total time for the glipizide challenge. All data underlying the findings described in this manuscript have been uploaded as a supplementary file (S3 File).

Mean ± standard deviation or median [interquartile range (IQR)], respectively, are provided for continuous normally or non-normally distributed traits unless otherwise specified. For statistical comparisons, non-normally distributed data were log-transformed, and all groups were compared using t-tests, and serial assessments within participants were compared using paired t-tests. Missing data were not imputed. If results at one assessment from a paired comparison were missing, the participant was excluded from analyses. Mean of group difference or paired difference and 95% confidence intervals are provided for all comparisons. For log-transformed data, mean of group log difference or paired log difference was derived from the mean comparison between log-transformed values at the two group or paired assessments, respectively. Association of selected endpoints with ethnicity was performed by linear regression models adjusted for age, sex, and BMI. Linear regression models, with and without age, sex, and ethnicity, were also used to test the association of genotype or genetic risk score on fasting glucose and insulin. The threshold for statistical significance in all analyses was set at two-tailed α = 0.05.

Statistical analyses were initially performed by NC using IBM SPSS Statistics for Windows (version 20.0; Armonk, NY) and independently confirmed by JT and MF using STATA (version 12.1; College Station, Texas). Genetic power calculations were performed using QUANTO (version 1.2.4; University of Southern Califonia). Figures were constructed using GraphPad Prism (version 5.00 for Windows; San Diego, CA).

## Results

### Participants

The demographic characteristics of the study population at two-thirds completion are summarized in Table 1; this cohort approximates the target population of individuals at risk of requiring future anti-diabetic therapy. More than 30% of the current cohort was from ethnic minority populations, average BMI was in the overweight or obese range, and participants were nearly evenly distributed between men and women.

**Table 1:** Demographic characteristics of SUGAR-MGH participants across ethnic groups.

	All participants (n = 653)	Non-Hispanic White (n = 436)	Non-Hispanic Black (n = 133)	Hispanic (n = 45)	Asian (n = 39)	*P* value
Men (n, %)	310 (47.5)	210 (48.2)	59 (44.4)	22 (48.9)	19 (48.7)	0.88^a^
Women (n, %)	343 (52.5)	226 (51.8)	74 (55.6)	23 (51.1)	20 (51.3)	
Age (yrs)	47.73±16.09	50.94±16.38	44.36±12.49	35.31±12.79	37.55±14.38	<0.0001^b^
BMI (kg/m^2^)	30.83±7.26	31.02±7.25	31.30±7.14	31.01±7.41	26.99±5.07	0.008^c^

Age and body mass index (BMI) are shown as mean ± standard deviation.

^*a*^ *P*-value for Fisher’s Chi-Square for sex.

^*b*^ *P*-value for ANOVA of age; Tukey’s pairwise comparison was significant at *P*<0.05 for non-Hispanic White vs. non-Hispanic Black, non-Hispanic White vs. Asian, non-Hispanic White vs. Hispanic, and Hispanic vs. non-Hispanic Black.

^c^ *P*-value for ANOVA of BMI; Tukey’s pairwise comparison was significant at *P*<0.05 for Asian vs. non-Hispanic White and Asian vs. non-Hispanic Black.

**Fig 2:**
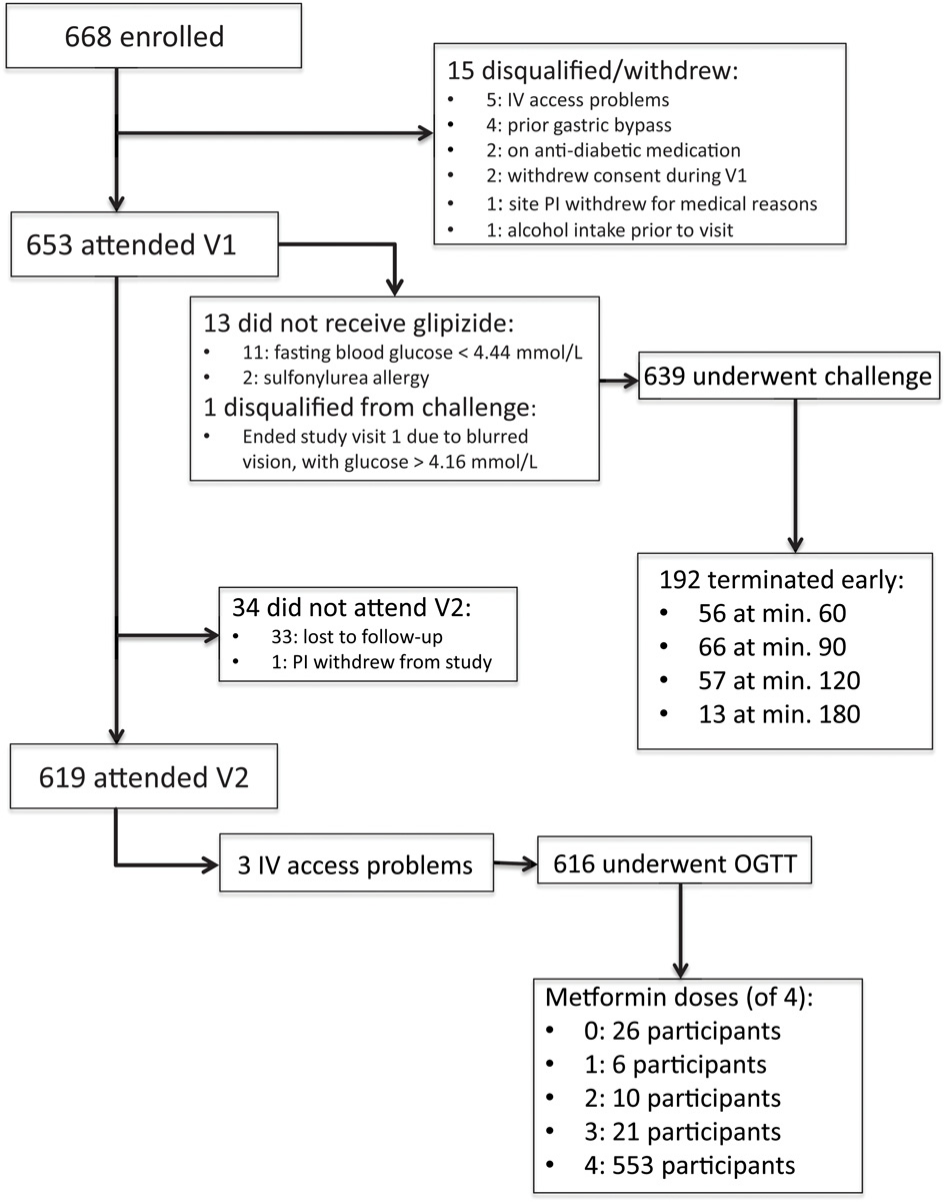
SUGAR-MGH Recruitment and study flow sheet.

Among 1,797 individuals screened for SUGAR-MGH, 1,324 were found to be eligible, and 668 were enrolled as of March 2013. The participant disposition following enrollment is depicted in Fig. 2. Of the 668 enrolled, 13 participants did not receive glipizide at the initial visit. The most common reason for not administering glipizide at Visit 1 was a fasting capillary blood glucose value <4.44 mmol/L. Six hundred and thirty-nine participants underwent the glipizide challenge, and 192 (30.0%) terminated the observation period earlier than 240 minutes due to reaching one of the pre-specified safety thresholds for hypoglycemia. No participant experienced a hypoglycemia-related serious adverse event, and no participant required intravenous glucose or glucagon to treat hypoglycemia. Six hundred and nineteen participants (94.8% of those who attended Visit 1) attended Visit 2. The most common reason for loss to follow-up in Visit 2 was failure to attend the visit by the study participant. There was no difference between the participants who attended and who did not attend Visit 2 with respect to age, sex, BMI, fasting plasma glucose, fasting insulin, race or ethnicity, or any other measured baseline characteristic (all *P*> 0.05). The majority of Visit 2 participants took four doses of metformin as specified in the protocol; approximately 10% took three or fewer doses.

### Biochemical response to glipizide

**Fig 3:**
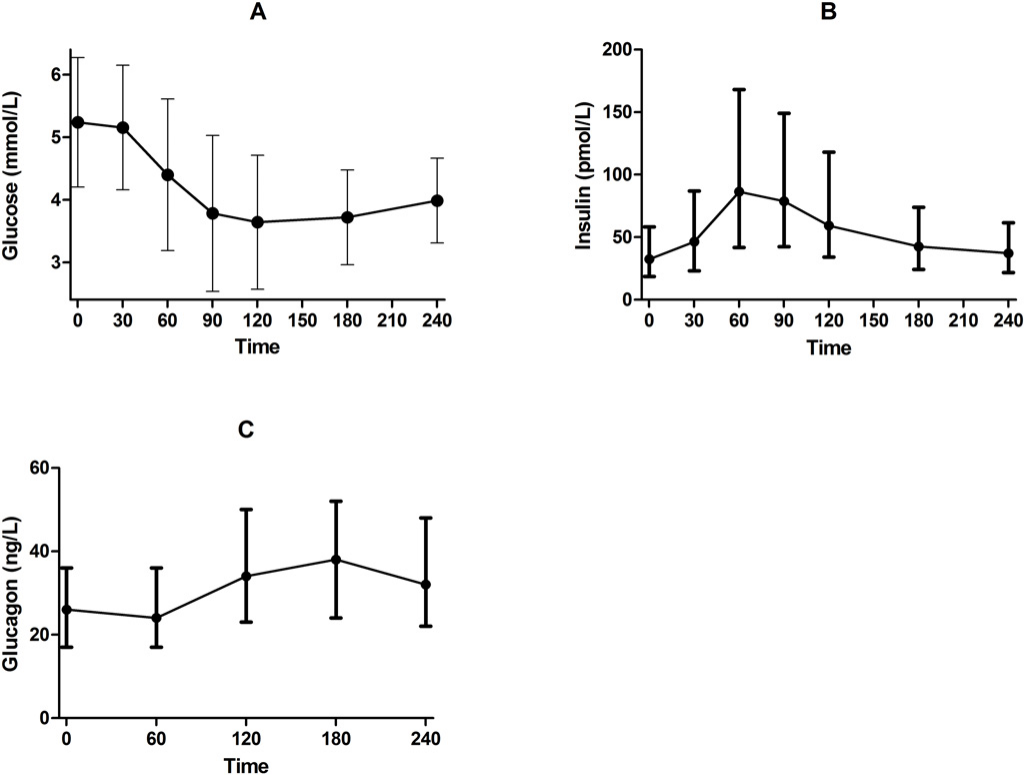
Glucose and insulin changes during glipizide challenge. Shown are A) mean ± standard deviation for blood glucose (mmol/L), B) median [IQR] for insulin (pmol/L), and C) median [IQR] for glucagon (ng/L) values prior to and during the glipizide challenge.

Glipizide raised serum insulin and lowered blood glucose, as expected (Figs. 3A and 3B). Insulin peaked at 60 minutes, and blood glucose values nadired at 120 minutes. Glucagon levels during the glipizide challenge peaked at 180 minutes, a time point after mean blood glucose had reached its lowest value (Fig. 3C).

**Fig 4:**
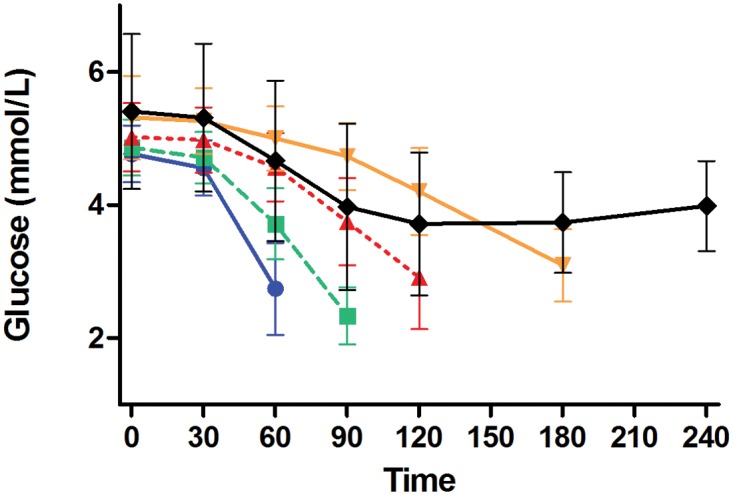
Glucose values during glipizide challenge stratified by time of challenge termination. Shown are mean ± standard deviation for blood glucose (mmol/L) during the glipizide challenge stratified by the time of challenge termination: 60 minutes (blue solid line with blue circles), 90 minutes (green dashed line with green squares), 120 minutes (red dashed line with red triangles), 180 minutes (orange solid line with orange inverted triangles), and 240 minutes (black solid line with black diamonds).

Approximately 30% of participants terminated the glipizide challenge early due to hypoglycemia or hypoglycemia-related symptoms, with 56, 66, 57, and 13 participants terminating at 60, 90, 120, and 180 minutes, respectively; 447 subjects completed the entire challenge. Participants who completed the entire glipizide challenge had higher fasting glucose values prior to receiving glipizide and higher trough glucose during the glipizide challenge than did participants who terminated the glipizide challenge due to hypoglycemia or hypoglycemia-related symptoms (Fig. 4). Specifically, mean fasting blood glucose levels of participants who completed 240 minutes of the glipizide challenge were significantly higher than those of participants who terminated the challenge at 60 min (5.41 ± 1.17 *vs*. 4.77 ± 0.43 mmol/L respectively; group difference 0.64 [95% CI 0.33, 0.95]; *P*<0.0001), at 90 min (4.86 ± 0.42 mmol/L; group difference 0.55 [95% CI 0.26, 0.83]; *P*<0.0001) and at 120 min (5.02 ± 0.51 mmol/L; group difference 0.39 [95% CI 0.08, 0.70]; *P*<0.0001), but were not different from those of participants who terminated the challenge at 180 min (5.32 ± 0.63 mmol/L; group difference 0.09 [95% CI -0.54, 0.73]; *P* = 0.61). Mean glucose trough values of participants who completed 240 minutes of the glipizide challenge were significantly higher than those participants who terminated the challenge at 60 min (3.18 ± 0.74 *vs*. 2.74 ± 0.69 mmol/L respectively; group difference 0.43 [95% CI 0.23, 0.64]; *P*<0.0001), at 90 min (2.33 ± 0.43 mmol/L; group difference 0.85 [95% CI 0.66, 1.03]; *P*<0.0001) or at 120 min (2.85 ± 0.72 mmol/L; group difference 0.33 [95% CI 0.12, 0.53]; *P* = 0.002), but were not different from those of participants who terminated the challenge at 180 min (3.06 ± 0.51 mmol/L; group difference 0.12 [95% CI -0.28, 0.53]; *P* = 0.43).

### Biochemical response to metformin

Biochemical measures at Visit 1 and Visit 2 are shown in Table 2. Among all participants, fasting glucose, insulin, HOMA-IR, and proinsulin were all lower at Visit 2 than at Visit 1 (*P* = 0.0008, *P* = 0.003, *P* = 0.0004, and *P*<0.0001, respectively, Table 2); fasting glucagon was not different between the two visits (*P* = 0.32, Table 2). In a sub-analysis, the change in HOMA-IR was similar in the whole cohort and when women on hormonal contraceptives or women under age 55 years were excluded, suggesting that the effect of metformin on insulin sensitivity was not strongly influenced by changes in menstrual cycle.

**Table 2:** Biochemical response to metformin.

	**Visit 1** ^a^	**Visit 2** ^a^	**Paired difference Mean (95% CI)**	***P*-value** ^b^
Glucose (mmol/L)	5.27 ± 1.06	5.08 ± 0.94	-0.20 (-0.31, -0.08)	0.0008
Took any metformin	5.27 ± 1.07	5.04 ± 0.89	-0.23 (-0.34, -0.11)	0.0002
Took no metformin	5.42 ± 0.08	5.85 ± 1.48	0.44 (-0.28, 1.15)	0.22
Insulin (pmol/L)	36.12 [22.62, 61.46]	29.97 [17.36, 57.60]	-0.16 (-0.26, -0.05)	0.003
Took any metformin	36.18 [22.76, 61.65]	29.37 [17.21, 56.48]	-0.19 (-0.29, -0.08)	0.0006
Took no metformin	30.03 [17.22, 55.04]	51.87 [27.44, 86.62]	0.44 (-0.91, 0.03)	0.07
HOMA-IR (mmol*pmol/L^2^)	8.31 [4.99, 14.29]	6.49 [3.60, 13.22]	-0.20 (-0.30, -0.09)	0.0004
Took any metformin	8.31 [4.99, 14.33]	6.31 [3.50, 12.70]	-0.23 (-0.34, -0.12)	<0.0001
Took no metformin	8.33 [4.12,12.24]	13.19 [5.93, 27.88]	0.50 (-0.02, 1.03)	0.06
Glucagon (ng/L)	26.00 [17.00, 37.00]	26.00 [17.00, 36.00]	0.02 (-0.06, 0.02)	0.32
Took any metformin	26.00 [17.00, 36.00]	26.00 [17.00, 36.00]	-0.02 (0.06, 0.02)	0.35
Took no metformin	32.00 [22.00, 46.00]	24.00 [19.00, 42.00]	-0.04 (-0.26, 0.17)	0.68
Proinsulin (mmol/L)	14.0 [8.70, 24.25]	12.25 [7.70, 22.00]	-0.12 (-0.16, -0.08)	<0.0001
Took any metformin	13.75 [8.45, 24.00]	12.00 [7.53, 21.00]	-0.13 (-0.17, -0.09)	<0.0001
Took no metformin	17.50 [13.38, 29.25]	18.00 [11.75, 30.75]	0.07 (-0.08, 0.21)	0.33

Values are shown at Visit 1 and Visit 2 for all participants and stratified by those participants who took no or any number of (1, 2, 3, or 4) metformin pills.

^a^ The mean ± SD for glucose and median [interquartile range] for non-normally distributed measures (insulin, HOMA-IR, glucagon, and proinsulin) are shown at Visit 1 and Visit 2. For statistical comparison, non-normally distributed data were log-transformed and paired difference for log-transformed measures is the mean of the log-transformed value at Visit 2 minus log-transformed value at Visit 1.

^b^ *P*-value is for paired t-test for glucose and for log-transformed insulin, HOMA-IR, glucagon, and proinsulin.

SD: standard deviation. CI: confidence interval. HOMA-IR: homeostatic model assessment of insulin resistance.

For participants who took any number of metformin doses (1, 2, 3, or 4; n = 583), fasting blood glucose, fasting insulin, and fasting HOMA-IR were lower at Visit 2 than at Visit 1 (*P* = 0.0002, *P* = 0.0006, and *P*<0.0001, respectively, Table 2). For participants who took no doses of metformin (n = 26), fasting blood glucose, fasting insulin, and fasting HOMA-IR were not different at Visit 1 and Visit 2 (*P* = 0.22, *P* = 0.07, *P* = 0.06, respectively, Table 2). As a result, fasting blood glucose, fasting insulin, and fasting HOMA-IR at Visit 2 were lower for participants who took any dose of metformin as compared with participants who took no dose of metformin (5.04 ± 0.89 *vs*. 5.85 ± 1.48 mmol/L; group difference -0.81 [95% CI -1.18, -0.45], *P*<0.0001 for glucose; 29.37 [17.21, 56.48] *vs*. 51.87 [27.44, 86.62] pmol/L, group log difference -0.43 units [95% CI -0.80, -0.07], *P* = 0.02 for insulin; and 6.31 [3.50, 12.70] *vs*. 13.19 [5.93, 27.88] mmol*pmol/L^2^, group log difference -0.57 units [95% CI -0.96, -0.18], *P* = 0.004 for HOMA-IR). There was no difference in the magnitude of fasting glucose, insulin, or HOMA-IR change between Visit 1 and Visit 2 between participants who took 1, 2, 3, or 4 metformin doses (n = 6, 10, 21, and 546, respectively).

**Fig 5:**
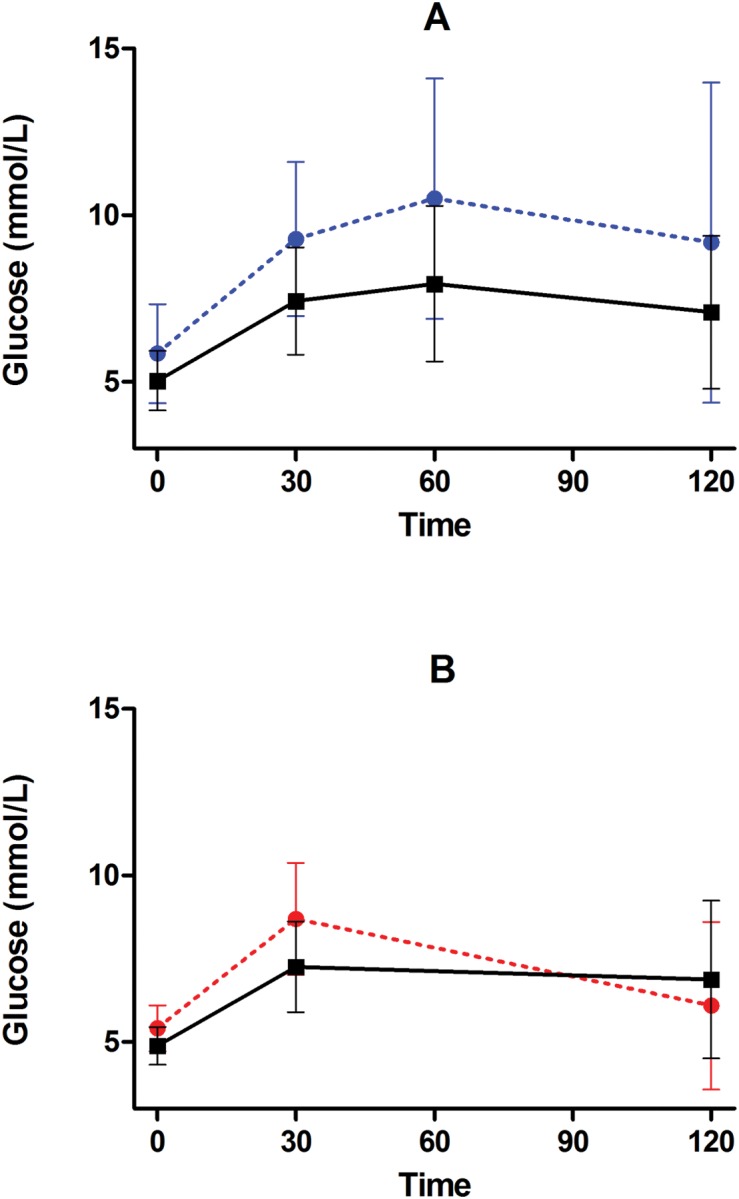
Glucose values during oral glucose tolerance test (OGTT) in the presence and absence of metformin. Shown are mean ± standard deviation for A) blood glucose (mmol/L) prior to and during the SUGAR-MGH oral glucose tolerance test (OGTT) stratified by participants who took no metformin doses (blue dashed line with blue circles) and participants who took any dose of metformin (black solid line with black squares); B) blood glucose (mmol/L) prior to and during an oral glucose tolerance test in the subset of participants who underwent an OGTT as part of the CAMP MGH study before receiving metformin (red dashed line with red circles) and after receiving four doses of metformin as part of the SUGAR-MGH study (black solid line with black squares).

During the OGTT in the presence of metformin, glucose and insulin both increased, as expected (S1 Fig.). After adjustment for differences in fasting glucose, glucose AUC during the OGTT was significantly higher in participants who took no metformin doses as compared with participants who took any dose of metformin (424.0 ± 216.9 *vs*. 267.1 ± 150.9 min*mmol/L; group difference 156.8 [95% CI 94.0, 219.7]; *P* = 0.002, Fig. 5A). There was no statistically significant difference in glucose AUC during the OGTT among participants who took one, two, three, or four metformin doses after adjustment for baseline glucose.

A subset of SUGAR-MGH participants had undergone fasting glucose and insulin measures and had completed an OGTT in the absence of metformin or other anti-hyperglycemic agents as part of another study (CAMP MGH). Participants had completed the CAMP MGH study an average of 10 months (range 3.4 years to 2 days) before completing the SUGAR-MGH study. Among these participants, all of whom took four doses of metformin during SUGAR-MGH, fasting glucose, fasting insulin, and HOMA-IR were higher prior to the metformin intervention than after the metformin intervention (5.47 ± 0.78 *vs*. 4.89 ± 0.61 mmol/L; paired difference 0.58 [95% CI 0.44, 0.73], *P*<0.0001; 61.80 [26.34, 96.00] vs.32.04 [12.96, 57.09] pmol/L; paired log difference 0.57 units [95% CI 0.43, 0.72]; *P*<0.0001; and 16.48 [5.74, 26.32] *vs*. 7.47 [2.60, 13.76] mmol*pmol/L^2^; paired log difference 0.68 units [95% CI 0.53, 0.84]; *P*<0.0001, respectively). After adjustment for fasting glucose, the glucose AUC during the OGTT in the absence of metformin was the same as the glucose AUC during OGTT in the presence of metformin (236.1 ± 161.0 *vs*. 236.9 ± 126.2 min*mmol/L; paired difference -0.85 [95% CI -43.8, 26.7]; *P* = 0.63 Fig. 5B). Insulin sensitivity, as estimated by the Matsuda index [25], was higher in the presence of metformin than in the absence of metformin (20.14 ± 16.0 units in the absence of metformin; paired difference 20.3 [95% CI 12.5, 28.0]; *P*<0.0001).

### Novel phenotypes for acute pharmacological and physiological responses

SUGAR-MGH is a resource to understand the acute effects of pharmacological perturbations. With adequate description of the biological responses to these interventions at two-thirds study enrollment, we here describe phenotypes of the human response to acute glipizide and metformin challenges. Upon full enrollment in SUGAR-MGH, these novel phenotypes can be employed in SUGAR-MGH to understand the influence of genetic variation on the human response to metformin and glipizide.

Phenotypes of glipizide response (Table 3A) were divided into those that are glucose-based (attempting to reflect a clinically relevant endpoint) and insulin-based (attempting to reflect a more proximal pharmacodynamic endpoint). We also considered several potential glucose-based measures to define the counter-regulatory response during the recovery from hypoglycemia. During the glipizide challenge, both glucose trough (S2 Fig.) and time to glucose trough differed across participants (S3 Fig.). Therefore, we considered phenotypes that captured both the magnitude and time component of the glipizide response: 1) glucose trough adjusted for baseline glucose (S4 Fig.), 2) time to glucose trough with adjustment for baseline glucose (S5 Fig.), and 3) insulin peak adjusted for baseline insulin (S6 Fig.). For the counter-regulatory recovery after hypoglycemia, we selected the slope in glucose between trough and the end of the study Visit 1 (240 minutes, S7 Fig.), capturing both magnitude and time in one measure. This measure of counter-regulatory response excludes participants who, due to hypoglycemia, received rescue carbohydrate and therefore reflects the endogenous response to hypoglycemia driven by counter-regulatory hormones alone.

**Table 3:** Proposed glipizide and metformin challenge endpoints.

Glipizide Challenge	Metformin Challenge
**Glucose-based**	**Glucose-based**
Glucose trough	Δ fasting glucose Visit 1 and Visit 2
Glucose trough adjusted for baseline glucose *	Fasting glucose at Visit 2 adjusted for fasting glucose at Visit 1 *
Δ (glucose 0 min to trough)	Δ fasting glucose Visit 1 and Visit 2 adjusted for BMI
Δ (glucose 0 to 90 min)	Fasting glucose at Visit 2 adjusted for baseline glucose at Visit 1 and BMI
Glucose at 120 min adjusted for baseline glucose	
Δ (glucose 0 to 120 min)	
Area over the glucose curve from 0 to 120 min	
Time to glucose trough *	
Time to glucose trough adjusted for baseline glucose *	
Δ (glucose 0 min to trough)/time to trough	
**Insulin-based**	**Insulin-based**
Insulin peak	Δ fasting insulin Visit 1 and Visit 2
Insulin peak adjusted for baseline insulin *	Fasting insulin at Visit 2 adjusted for fasting insulin at Visit 1
Insulin at 60 min adjusted for baseline insulin	Δ fasting insulin Visit 1 and Visit 2 adjusted for BMI
Δ (insulin 0 min to peak insulin)	Fasting insulin at Visit 2 adjusted for fasting insulin at Visit 1 and BMI
Δ (insulin 0 to 60 min)	
Area under the insulin curve from 0 to 240 min	
Time to insulin peak	
Δ (insulin 0 min to peak insulin) / time to peak insulin	
**Recovery period**	**HOMA-IR based**
Δ (glucose 120 to 240 min)	Δ HOMA-IR Visit 1 and Visit 2
Δ (glucose trough to 240 min)	HOMA-IR at visit 2 adjusted for HOMA-IR at Visit 1
Δ (glucose trough to 240 min) /time from trough to 240 min *	Δ HOMA-IR Visit 1 and Visit 2 adjusted for BMIHOMA-IR at Visit 2 adjusted for HOMA-IR at Visit 1 and BMI

Δ: change

* Selected endpoints

Δ: change

BMI: body mass index; HOMA-IR: homeostatic model assessment of insulin resistance.

Novel phenotypes of metformin response (Table 3B) were divided into those that were glucose-based, insulin-based, and HOMA-IR based. Fasting glucose at Visit 2, adjusted for fasting glucose at Visit 1 (S8 Fig.), was chosen as the most robust, clinically relevant phenotype.

The putative effect of ethnicity on all response phenotypes was tested. The glucose and insulin changes following glipizide administration in each ethnic group are provided in S9 Fig. The glucose and insulin changes following the 75-g oral glucose load in the presence of metformin in each ethnic group are provided in S10 Fig. After adjusting for age, sex, and BMI, there were no statistically significant differences in glucose or insulin-based endpoints after glipizide or metformin by ethnic group. These analyses may be underpowered and will need to be confirmed in the completed cohort.

### Genetic association preliminary results

While genetic analyses performed at two-thirds study enrollment may be underpowered, we tested whether known genetic associations with sufficiently large effects could be validated currently in SUGAR-MGH.

First, we tested whether the T risk allele at the single nucleotide polymorphism (SNP) rs7903146 in the type 2 diabetes-associated locus *TCF7L2* was associated with higher fasting glucose in our cohort, as previously shown by the MAGIC investigators [26]. Consistent with these published findings, which showed an effect of +0.023 ± 0.004 mmol/L*allele in a cohort of > 45,000 individuals, the same risk allele was associated with higher fasting glucose in 527 SUGAR-MGH participants with genotype information (β = +0.139 ± 0.05 mmol/L*allele, *P* = 7×10^-3^).

Next we tested whether an aggregate genetic risk score (GRS) composed of SNPs confirmed to be associated with glycemic traits in genome-wide association studies [26–28] are associated with fasting glucose and insulin levels in SUGAR-MGH. The GRSs were constructed by summing the number of risk alleles carried by each participant at loci previously associated with fasting glucose (for the fasting glucose GRS) or with fasting insulin (for the fasting insulin GRS). Thus, we combined 34 SNPs associated at genome-wide significance with higher fasting blood glucose levels and 14 SNPs associated with higher fasting insulin levels (S1 Table) to construct a GRS for fasting blood glucose levels (range 0–68 on the basis of the number of risk alleles carried per individual) and a separate GRS for fasting insulin levels (range 0–28). The median fasting blood glucose GRS was 36 (range 24–47). Prior to adjustment for demographic characteristics, the association between fasting blood glucose GRS and fasting blood glucose values at Visit 1 was not significant but trended in the expected direction of effect (β = +0.0113 mmol/L*allele, *P* = 0.215). After adjustment for age, sex, and ethnicity, the relationship between the fasting glucose GRS and fasting glucose became significant (β = +0.0256 mmol/L*allele, *P* = 0.006). The increase in effect size was due to inclusion of the ethnicity co-variate in the model, and models corrected for age and/or sex did not substantially change the β-coefficient and remained non-significant. The relationship between the fasting glucose GRS and fasting glucose was lower in Non-Hispanic Black participants (β = +0.004 ± 0.01 mmol/L*allele, *P* = 0.81) than in other race/ethnic groups (Non-Hispanic White β = +0.03 ± 0.01 mmol/L*allele, *P* = 0.01; Asian β = +0.03 ± 0.02 mmol/L*allele, *P* = 0.23; Hispanic β = +0.07 ± 0.03 mmol/L*allele, *P* = 0.02). The median fasting insulin GRS was 15 (range 5–23). The direction of effect between the insulin GRS and fasting insulin level trended in the expected direction but did not reach conventional statistical significance (β = +0.078 pmol/L*allele, *P* = 0.08).

## Discussion

It is highly important to translate the plethora of recent genetic findings for glycemic traits into clinically relevant outcomes [5, 29]. An initial step on this path involves the physiological characterization of loci associated with type 2 diabetes or one of its related phenotypes, as a way to establish their likely anatomical site or mechanism of action. In addition, one of the paramount promises of “precision medicine” is the potential use of genetic information to guide therapeutic choices: for that promise to be realized, tests of specific hypotheses must be performed rigorously. This requires the construction of appropriate resources in the relevant model system (the human) where both tasks can be carried out.

Indeed, pharmacological perturbations in humans may illustrate the function of associated genes while establishing whether differential genotypic responses exist. When performed on an acute time course, the influence of lifestyle and dietary changes that occur during longer-term studies can be limited. We have therefore begun to build a comprehensive resource that tests two limbs of the glycemic homeostatic system, having enrolled more than two thirds of our pre-specified recruitment goal of 1,000 participants. When SUGAR-MGH is completed, samples and data will be available for collaborative studies, and the great majority of our participants have consented for future follow-up investigation.

In SUGAR-MGH, we have achieved a safe glipizide challenge in the fasting state, with no subject experiencing severe hypoglycemia. As sulfonylurea medications are not generally administered in the fasting state, this perturbation is unique and allows characterization of sulfonylurea-related biology and the counterregulatory response in the absence of exogenous glucose. In addition, we have shown a measureable glycemic effect of short-term metformin treatment, comparable to that seen in other short-term studies [30] and similar in magnitude to that observed after 1 year of metformin treatment in the Diabetes Prevention Program [14]. The effect of this intervention impacts both fasting glucose and fasting insulin measures. Among participants who underwent an OGTT in both the absence and presence of metformin, fasting glucose captures most of the effect of acute metformin treatment, as differences in glucose AUC during an OGTT before and after metformin treatment are explained by the differences in the fasting measure. Interestingly, glucose AUC during an OGTT remains different between participants who took either 0 or any dose of metformin after adjustment for fasting glucose, suggesting that inter-individual variation, outside of the effect of metformin, contributes to glucose excursions. Thus, additional opportunity exists, within the completed SUGAR-MGH cohort, to examine genetic, environmental, and hormonal drivers of the heterogeneous response to glipizide and metformin in the fasting and dynamic states.

We have selected a number of novel phenotypes for the acute response to glipizide and metformin for potential pharmacogenetic experiments in this cohort. In addition, we have shown that there are no large ethnic influences on these phenotype outcomes, and that ethnic differences in genetic analyses can be controlled by our statistical methods. These findings will be confirmed in the completed cohort. And finally, as a proof of concept, we have shown that in this cohort the association of a polymorphism at *TCF7L2* and a GRS constructed on the basis of previously known associations with fasting glucose are consistent with expectations. These are provided as evidence that genetic associations can be detected in SUGAR-MGH even prior to completion of the full cohort.

After full enrollment of the SUGAR-MGH resource, the ability to test genetic associations with pharmacological responses will be adequately powered based on our calculations. For example, the completed resource will be able to test relationships between known glycemic genetic variants with aspects of glipizide and metformin response. Similarly, SUGAR-MGH can be used to test directly the effect of novel variants, discovered in other cohorts and with unknown functional implications, on the human response to glipizide, metformin, and oral glucose challenges.

Last, SUGAR-MGH could be employed to identify genetic variants that differentially influence the responses to the two pharmacological challenges. Given the different time scales of the interventions, this approach would require that a chosen glipizide phenotype (*e*.*g*. time to trough glucose adjusted for baseline glucose) be normalized across all participants and compared against a similarly normalized metformin phenotype (*e*.*g*, change in fasting glucose adjusted for baseline glucose). For this hypothetical example, there would be 85% power to detect an effect difference of 0.45 (medium to large effect) between the two endpoints for a variant with minor allele frequency of 5% in the SUGAR-MGH cohort. For a variant with a 20% minor allele frequency, there would be 85% power to detect an effect difference of 0.26 (small to medium effect). Given the large number of potential questions that can be tested using SUGAR-MGH, appropriate statistical thresholds, correcting for the number of hypotheses tested, must be used to limit false positive findings.

In summary, the SUGAR-MGH cohort illustrates a paradigm for the construction of a pharmacogenetic resource in humans, which is free of the uncontrolled nature of retrospective clinical datasets, achievable at a local site with an investigator-initiated award, and simple enough to enable the recruitment of a large cohort with excellent retention rates and short duration. Future studies will evaluate the impact of specific genetic variants on the outcomes described here, and guide the design of prospective pharmacogenetic clinical trials.

## Supporting Information

S1 Fig Glucose and insulin response following 75-g oral glucose load in the presence of metformin. Shown are mean ± standard deviation for blood glucose (mmol/L, black solid line with black circles, left axis) and median [IQR] for insulin (pmol/L, blue dashed line with blue squares, right axis)
(TIF)

S2 Fig Distribution of glucose trough following glipizide challenge. Shown is the number of participants at each trough glucose value (mmol/L) following administration of glipizide.
(TIF)

S3 Fig Distribution of time to glucose trough following glipizide challenge. Shown is the number of participants at each time point at which trough glucose (mmol/L) was reached following administration of glipizide. Data were not collected at 150 minutes or 210 minutes and these categories are subsequently empty.
(TIF)

S4 Fig Distribution of glucose trough following glipizide challenge adjusted for baseline glucose. Shown is the number of participants with residuals of the regression equation at each category in which glucose trough (mmol/L) was the dependent variable and baseline glucose (mmol/L) during the glipizide challenge was the covariate.
(TIF)

S5 Fig Distribution of time to glucose trough following glipizide challenge adjusted for baseline glucose. Shown is the number of participants with the residuals of the regression equation at each category in which time to glucose trough (minutes) was the dependent variable and baseline glucose (mmol/L) during the glipizide challenge was the covariate.
(TIF)

S6 Fig Distribution of insulin peak following glipizide challenge adjusted for baseline insulin. Shown is number of participants with residuals of the regression equation at each category in which insulin peak (pmol/L) was the dependent variable and baseline insulin (pmol/L) during the glipizide challenge was the covariate.
(TIF)

S7 Fig Distribution of the slope in glucose between time of trough following glipizide administration and the end of study visit 1 (240 minutes). Shown is the number of participants at each data point for the relationship between the difference in glucose at trough and end of study visit (mmol/L) divided by difference in time of trough and 240 minutes (minutes).
(TIF)

S8 Fig Distribution of the fasting glucose at Visit 2 adjusted for fasting glucose at Visit 1. Shown is the number of participants with the residual values of the regression equation at each category in which fasting glucose at second visit (mmol/L) was the dependent variable and fasting glucose at first visit (mmol/L) was the covariate.
(TIF)

S9 Fig Glucose and insulin responses following glipizide administration stratified by ethnic group. In panel A are shown the mean ± standard deviation for glucose (mmol/L) for White non-Hispanic (black solid line with black circles), Asian (red dashed line with red inverted triangles), Black non-Hispanic (blue dashed line with blue squares), and Hispanic (green solid line with green triangles). In panel B are shown the median [IQR] for insulin (pmol/L) for the same groups.
(TIF)

S10 Fig Glucose and insulin responses following 75-gram oral glucose load in the presence of metformin stratified by ethnic group. In panel A are shown the mean ± standard deviation for glucose (mmol/L) for White non-Hispanic (black solid line with black circles), Asian (red dashed line with red inverted triangles), Black non-Hispanic (blue dashed line with blue squares), and Hispanic (green solid line with green triangles). In panel B are the median [IQR] for insulin (pmol/L) for the same groups.
(TIF)

S1 File Trial Consent Form. (PDF)

S2 File Trial Consent Form. (PDF)

S3 File Study data. (XLSX)

S1 Protocol Trial Protocol. (DOCX)

S2 Protocol Trial Protocol. (DOCX)

S1 Table Investigated genetic markers. Single nucleotide polymorphisms (SNPs) previously associated with fasting glucose were used to calculate the fasting glucose genetic risk score (GRS); SNPs previously associated with fasting insulin were used to calculated the fasting insulin GRS. For each SNP, an effect allele, which raises fasting glucose or fasting insulin, is provided.
(DOCX)

S1 TREND Checklist TREND Checklist. (PDF)
