# Supplementary figures and images for: The Study to Understand the Genetics of the Acute Response to Metformin and Glipizide in Humans (SUGAR-MGH): Design of a pharmacogenetic Resource for Type 2 Diabetes
Source: PLoS One. 2015 Mar 26;10(3):e0121553. doi: 10.1371/journal.pone.0121553 (PMC4374872; doi:10.1371/journal.pone.0121553)

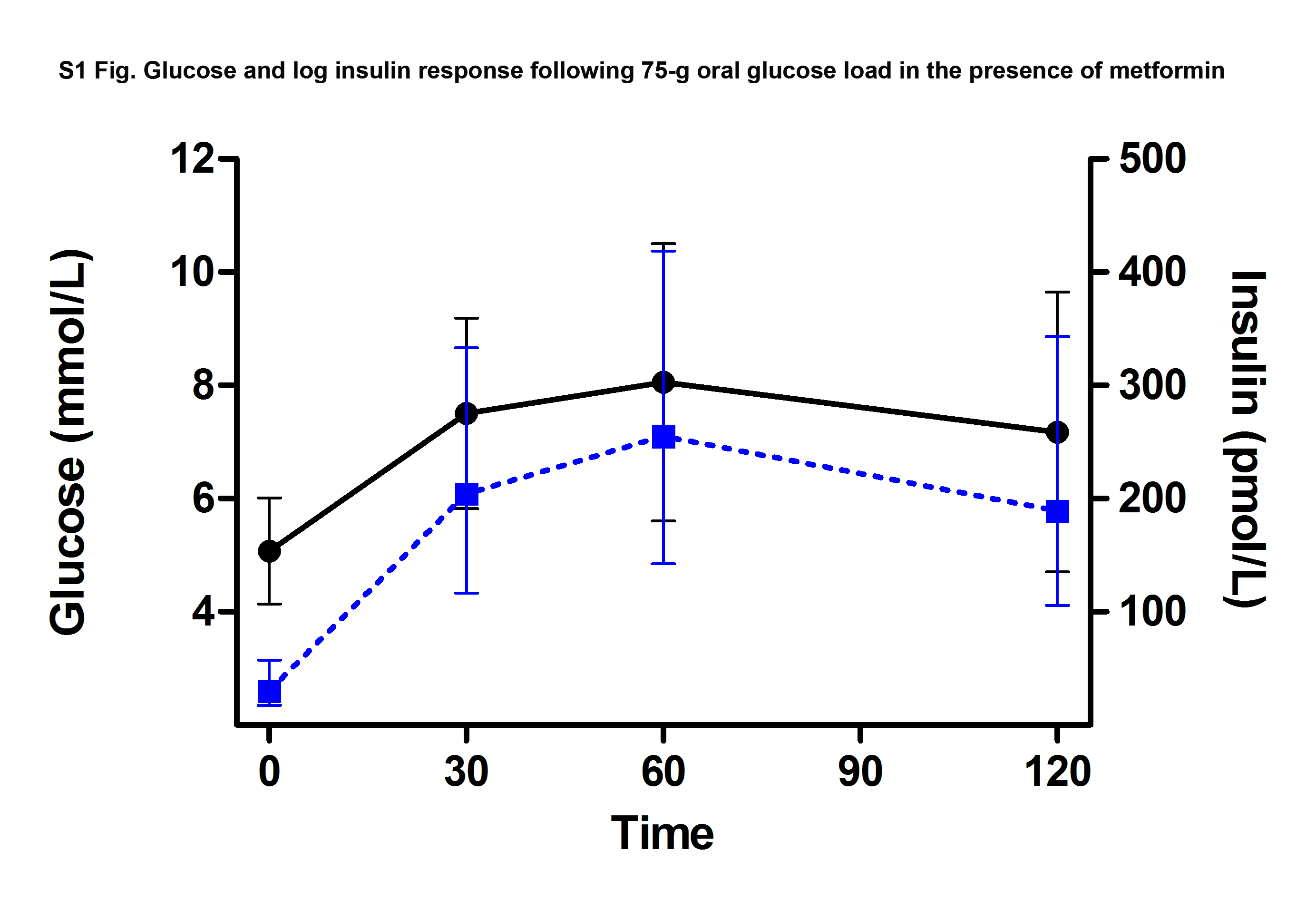

Supplement: S1 Fig — Shown are mean ± standard deviation for blood glucose (mmol/L, black solid line with black circles, left axis) and median [IQR] for insulin (pmol/L, blue dashed line with blue squares, right axis) (TIF) [file pone.0121553.s001.tif]

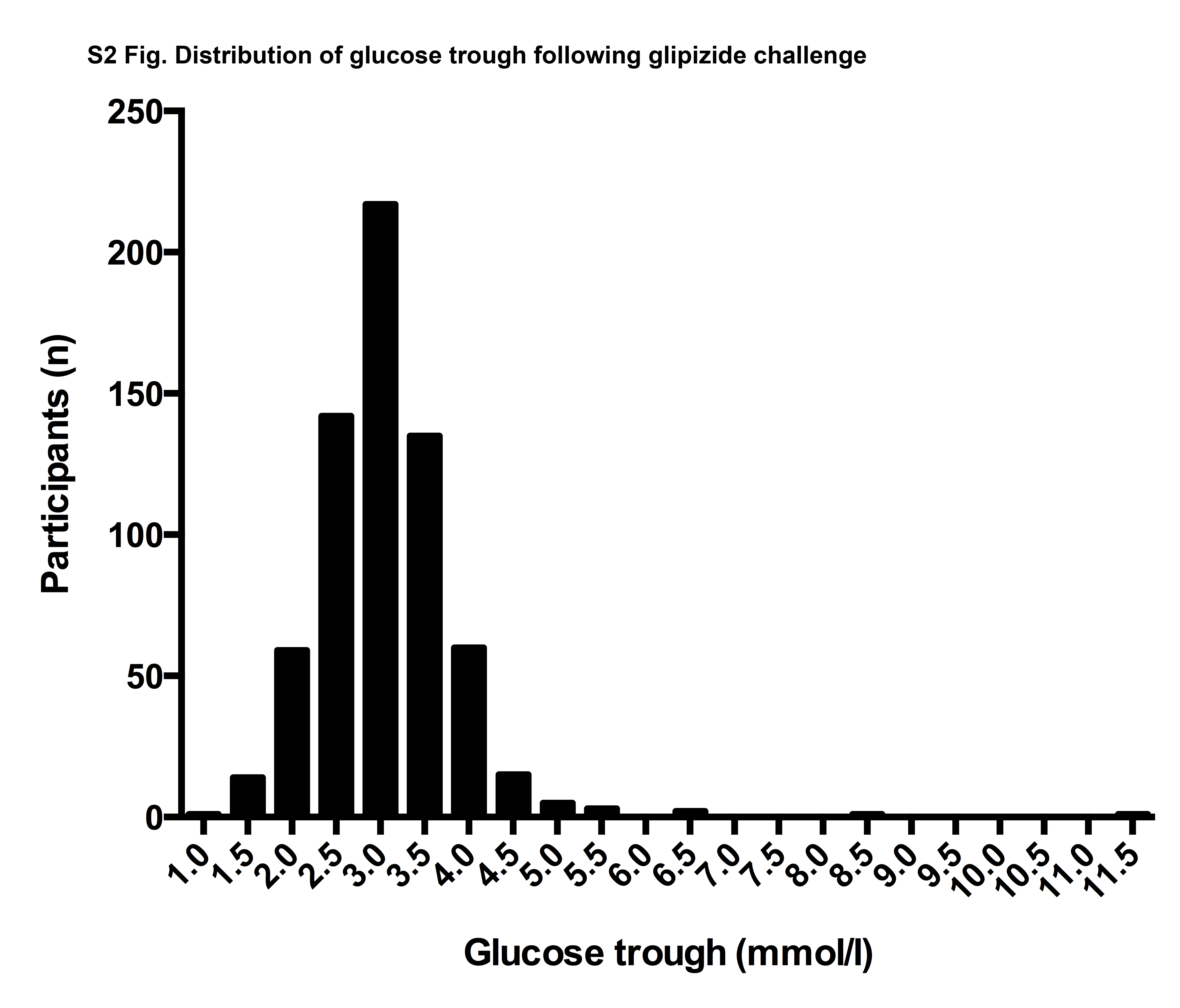

Supplement: S2 Fig — Shown is the number of participants at each trough glucose value (mmol/L) following administration of glipizide. (TIF) [file pone.0121553.s002.tif]

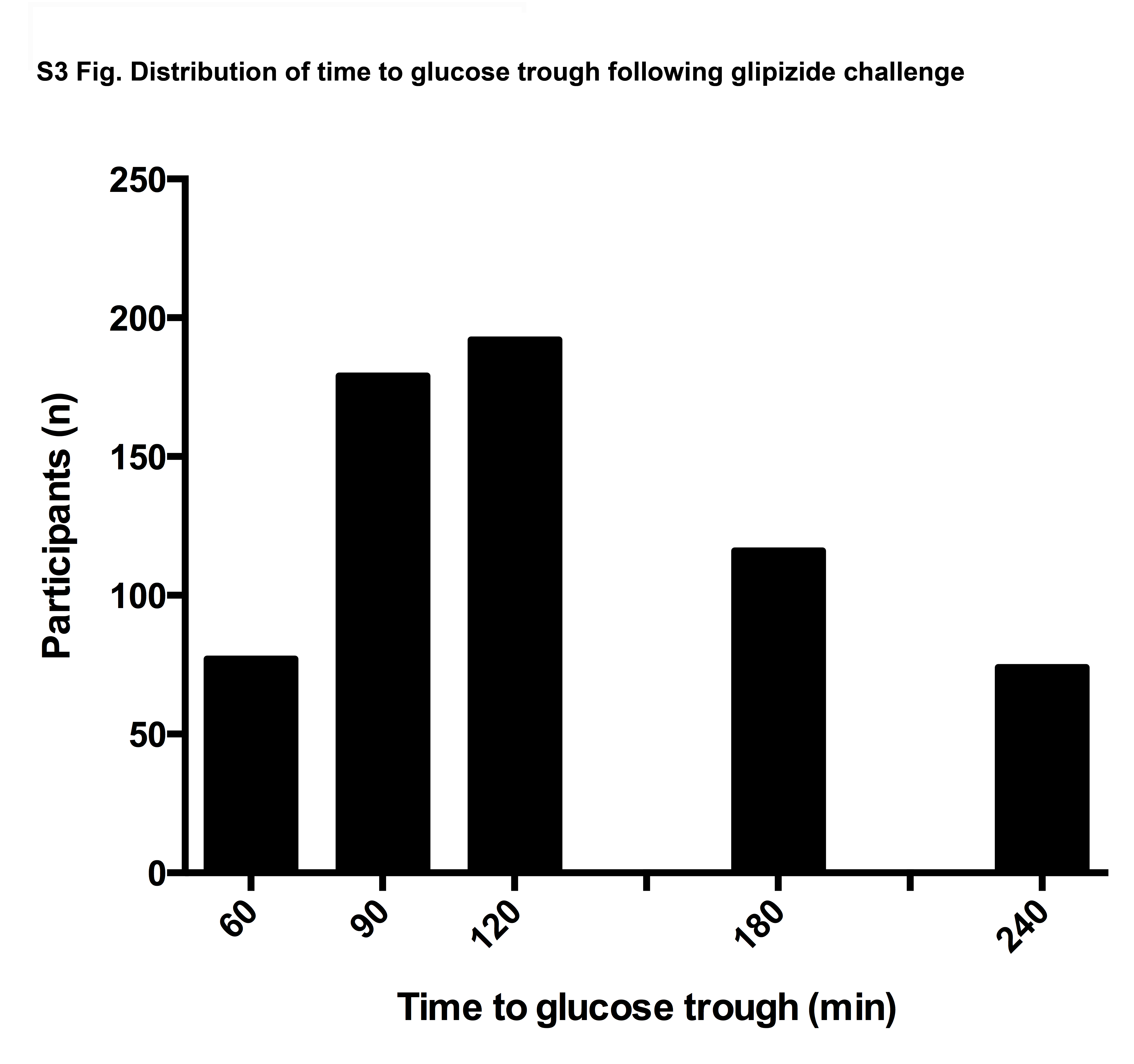

Supplement: S3 Fig — Shown is the number of participants at each time point at which trough glucose (mmol/L) was reached following administration of glipizide. Data were not collected at 150 minutes or 210 minutes and these categories are subsequently empty. (TIF) [file pone.0121553.s003.tif]

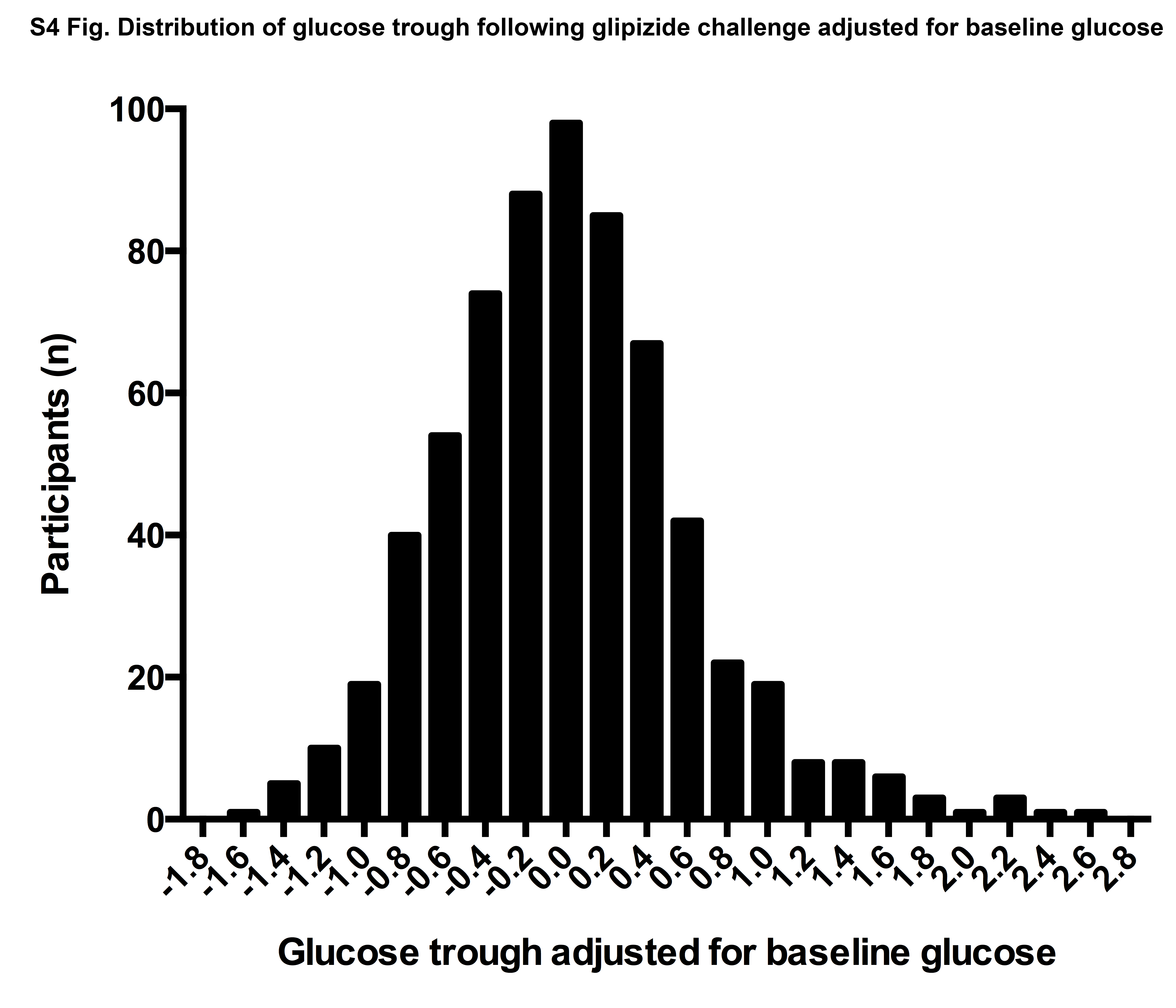

Supplement: S4 Fig — Shown is the number of participants with residuals of the regression equation at each category in which glucose trough (mmol/L) was the dependent variable and baseline glucose (mmol/L) during the glipizide challenge was the covariate. (TIF) [file pone.0121553.s004.tif]

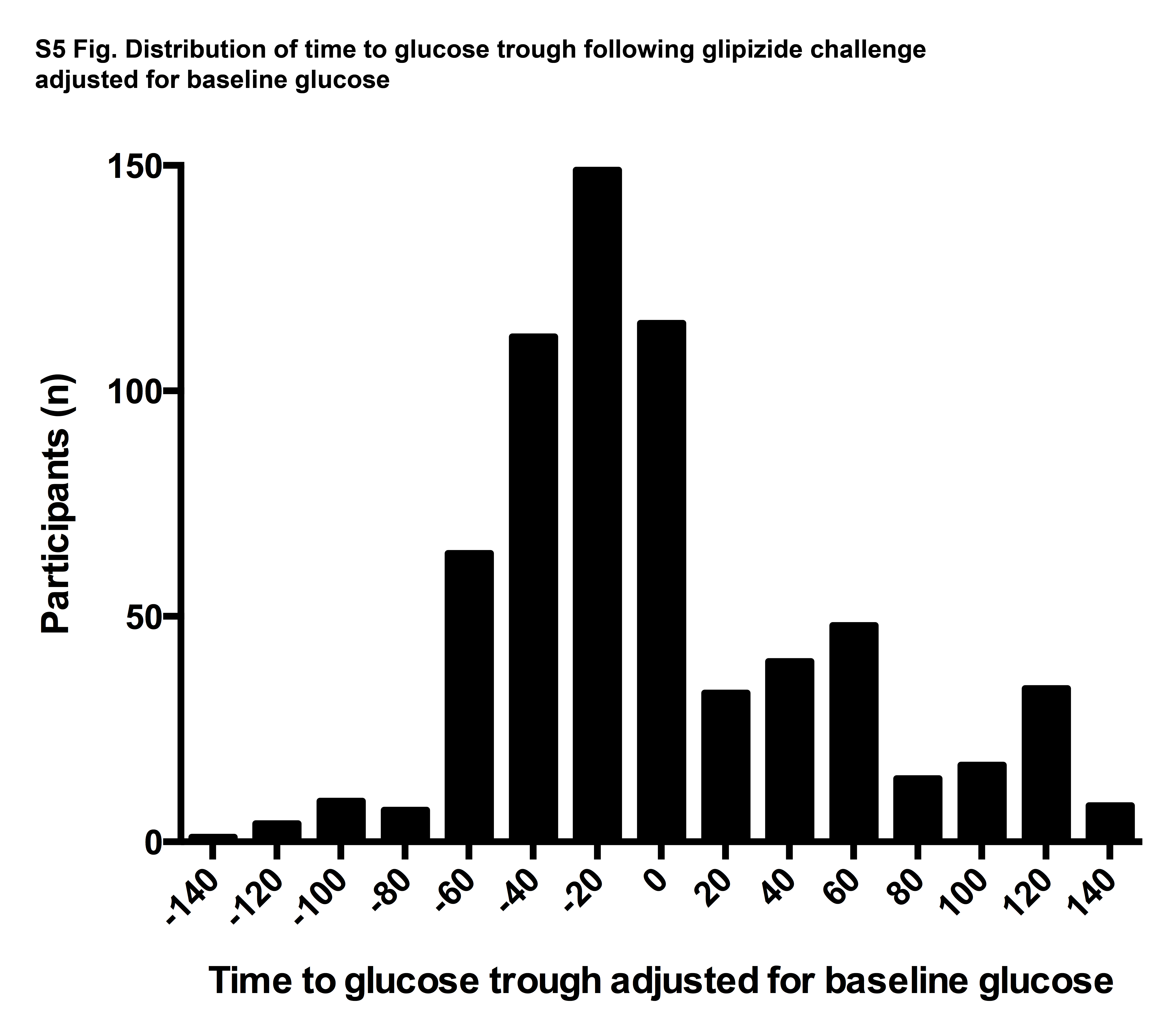

Supplement: S5 Fig — Shown is the number of participants with the residuals of the regression equation at each category in which time to glucose trough (minutes) was the dependent variable and baseline glucose (mmol/L) during the glipizide challenge was the covariate. (TIF) [file pone.0121553.s005.tif]

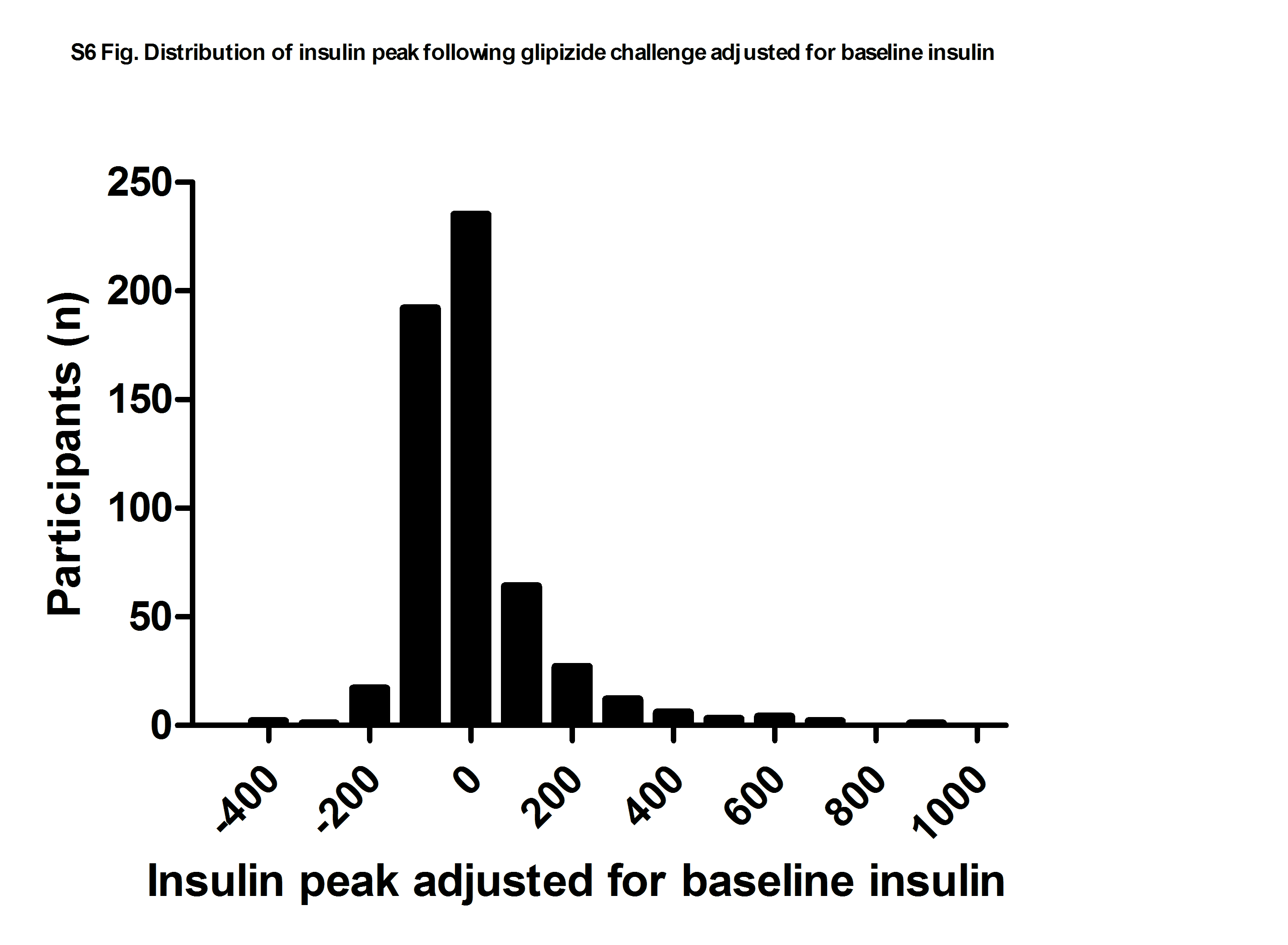

Supplement: S6 Fig — Shown is number of participants with residuals of the regression equation at each category in which insulin peak (pmol/L) was the dependent variable and baseline insulin (pmol/L) during the glipizide challenge was the covariate. (TIF) [file pone.0121553.s006.tif]

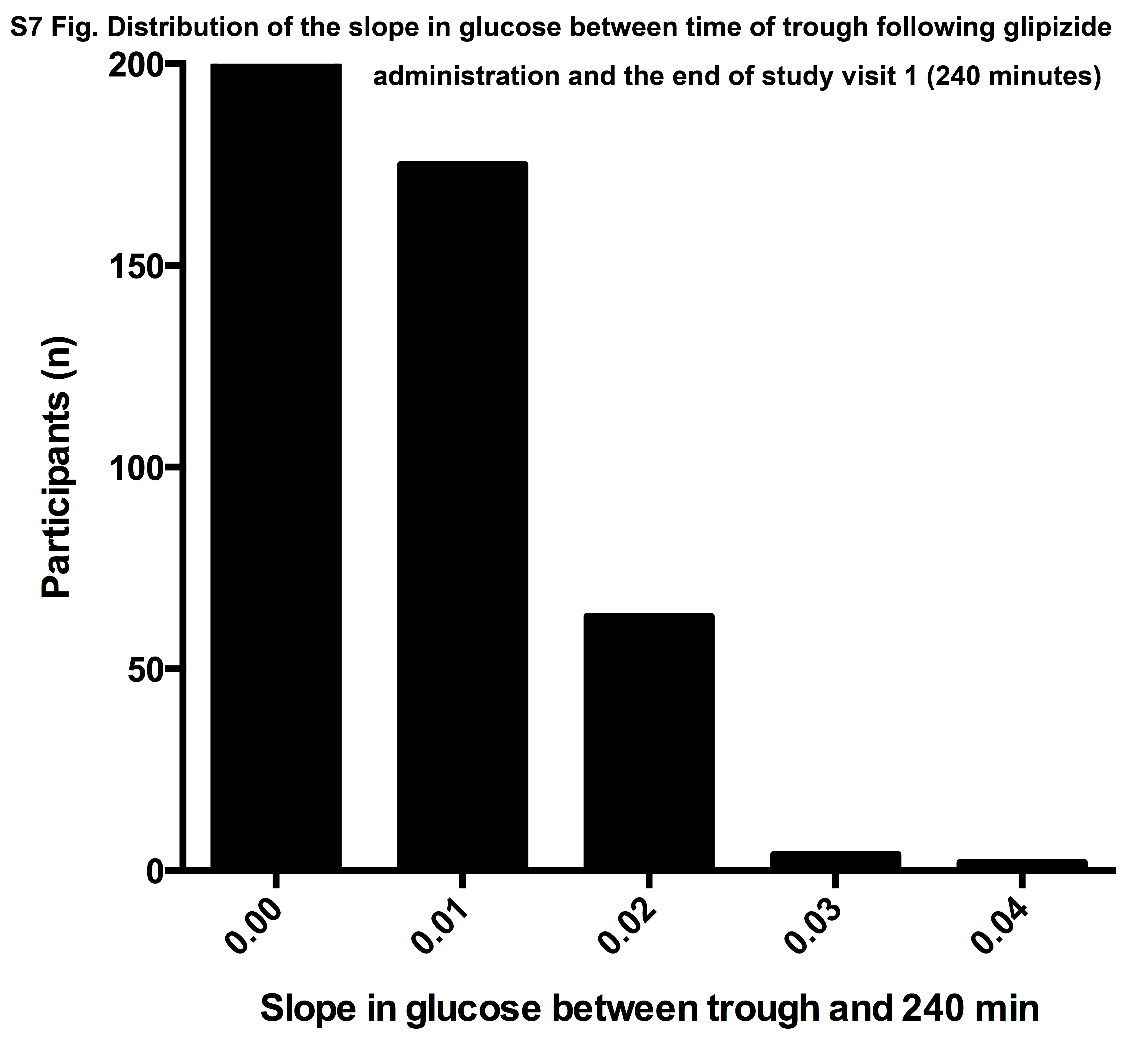

Supplement: S7 Fig — Shown is the number of participants at each data point for the relationship between the difference in glucose at trough and end of study visit (mmol/L) divided by difference in time of trough and 240 minutes (minutes). (TIF) [file pone.0121553.s007.tif]

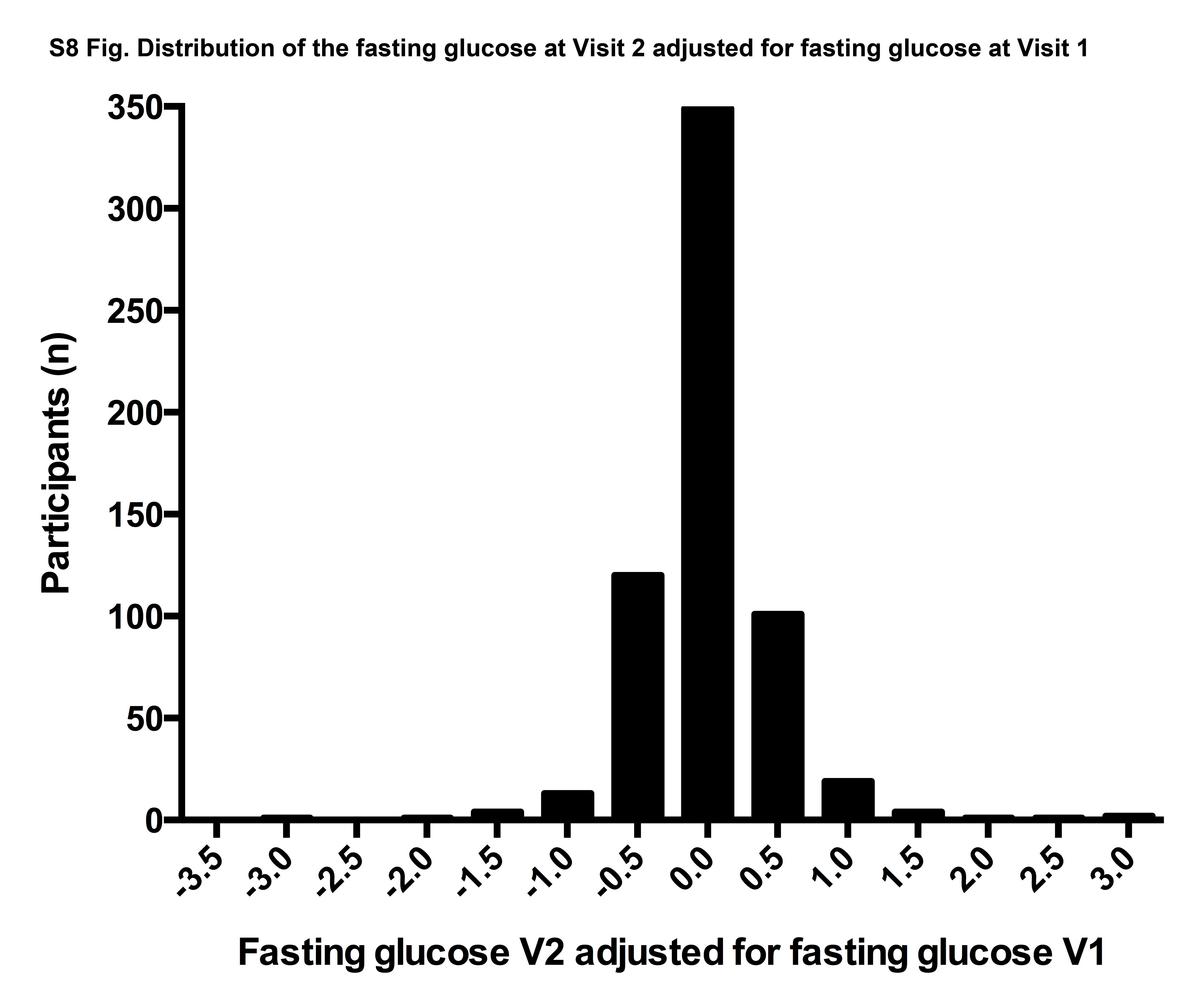

Supplement: S8 Fig — Shown is the number of participants with the residual values of the regression equation at each category in which fasting glucose at second visit (mmol/L) was the dependent variable and fasting glucose at first visit (mmol/L) was the covariate. (TIF) [file pone.0121553.s008.tif]

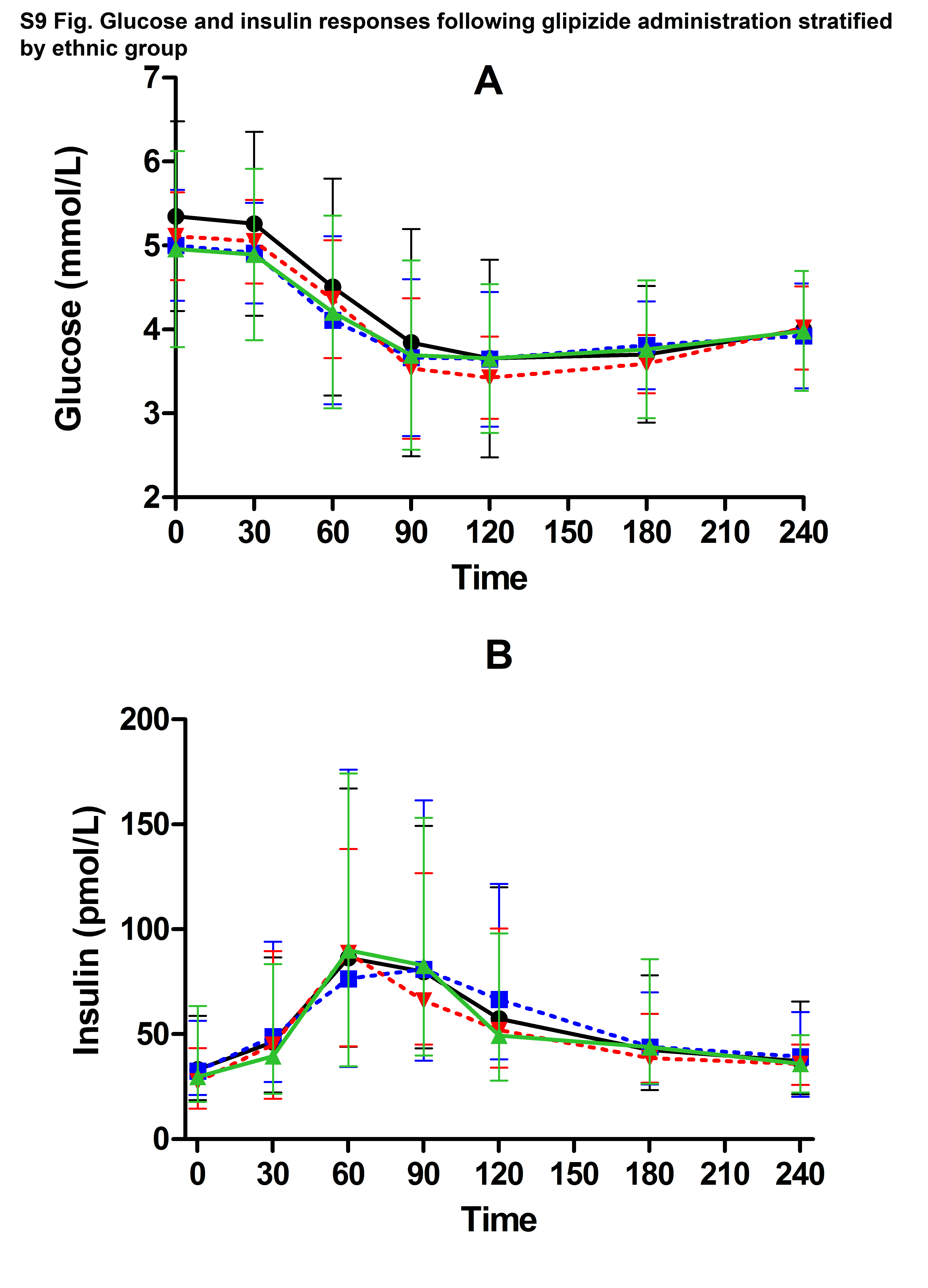

Supplement: S9 Fig — In panel A are shown the mean ± standard deviation for glucose (mmol/L) for White non-Hispanic (black solid line with black circles), Asian (red dashed line with red inverted triangles), Black non-Hispanic (blue dashed line with blue squares), and Hispanic (green solid line with green triangles). In panel B are shown the median [IQR] for insulin (pmol/L) for the same groups. (TIF) [file pone.0121553.s009.tif]

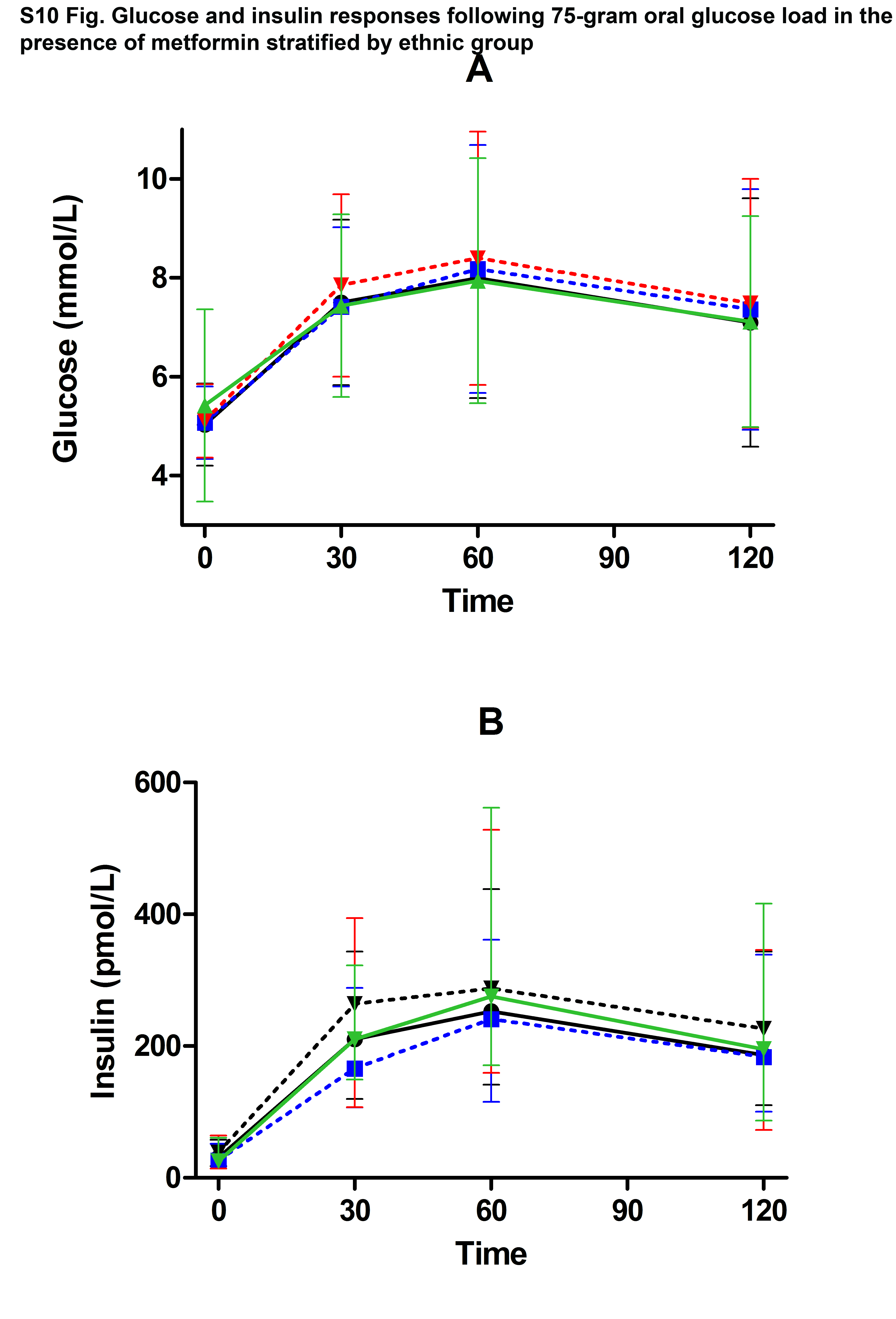

Supplement: S10 Fig — In panel A are shown the mean ± standard deviation for glucose (mmol/L) for White non-Hispanic (black solid line with black circles), Asian (red dashed line with red inverted triangles), Black non-Hispanic (blue dashed line with blue squares), and Hispanic (green solid line with green triangles). In panel B are the median [IQR] for insulin (pmol/L) for the same groups. (TIF) [file pone.0121553.s010.tif]
