# Supplementary material for: The Study to Understand the Genetics of the Acute Response to Metformin and Glipizide in Humans (SUGAR-MGH): Design of a pharmacogenetic Resource for Type 2 Diabetes
Source: PLoS One. 2015 Mar 26;10(3):e0121553. doi: 10.1371/journal.pone.0121553 (PMC4374872; doi:10.1371/journal.pone.0121553)
Supplement: S1 File — (PDF) [file pone.0121553.s011.pdf]

Partners HealthCare System
Research Consent Form

General Template
Version Date: November 2005

Subject Identification

Protocol Title: Study to Understand the Genetics of the Acute Response to Metformin and Glipizide in Humans (SUGAR MGH)

Principal Investigator: Jose C. Florez, MD, PhD

Site Principal Investigator: Massachusetts General Hospital

Description of Subject Population: Adults at risk of requiring antidiabetic medications

About this consent form

Please read this form carefully. It tells you important information about a research study. A member of our research team will also talk to you about taking part in this research study. People who agree to take part in research studies are called "subjects." This term will be used throughout this consent form. If you have any questions about the research or about this form, please ask us. If you decide to take part in this research study, you must sign this form to show that you want to take part. We will give you a copy of this form to keep.

Why is this research study being done?

The purpose of this research study is to examine whether changes in genes that influence the risk of type 2 diabetes affect how human beings respond to two anti-diabetic drugs. The drugs we are studying are glipizide and metformin. Genes are sections or pieces of DNA inherited from our parents. DNA is the material from which genes are made, which allows for the transfer of physical information from generation to generation. Genes determine many of our physical traits and how our bodies react to disease.

We are asking you to take part in this study:

- because you may have early type 2 diabetes or you may be at risk of developing type 2 diabetes, or
- because of your family history, your weight, and blood sugar measurements in your medical record, or other conditions.

The two drugs used in this study (glipizide and metformin) are approved by the U.S. Food and Drug Administration (FDA) for the treatment of type 2 diabetes. Glipizide lowers blood sugar (glucose), while metformin decreases the amount of glucose your liver makes. Metformin also improves the ability of other tissues (muscle and fat) to take glucose out of your bloodstream in response to the insulin your own pancreas makes to decrease blood sugar. Metformin, by itself, does not lower blood sugar. Metformin has been shown to delay the onset of type 2 diabetes in subjects at risk.

The effect of these drugs on human beings is similar regardless of whether the person taking them has diabetes or not. They are being used here to study how your genes affect your body's response to the drug, and not as the standard treatment for type 2 diabetes approved by the Federal Drug Administration.

About 1,000 subjects will take part in this study. We expect to enroll about 700 subjects at Massachusetts General Hospital (MGH).

How long will I take part in this research study?

It will take you one week to complete the study. During this time you will be asked to make 2 study visits, lasting about 5 to 6 hours (Visit 1) and 3 to 4 hours (Visit 2).

What will happen in this research study?

The following procedures will take place during this research study:

Screening Visit at the Clinical Research Center (CRC):

You are asked to come to the Clinical Research Center (CRC) at MGH for this visit. To prepare for the Screening Visit to find out if you qualify for the study, you will have an overnight fast. After midnight before the Screening Visit, you cannot have any food or drinks. You can drink water. In addition, please limit your alcohol intake the evening before to one or fewer drinks. One drink is defined as 12 ounces of beer, 5 ounces of wine or 1.5 ounces of liquor.

We will answer all questions that you may have about this study before you sign this consent form. We will ask a few questions in order to verify that you qualify for this study. If you are a woman of childbearing age who is currently sexually active we will do a urine pregnancy test. The CRC nurse will also measure your heart rate and blood pressure and take your height and

weight. If you are found to qualify for the study and are willing, you will immediately move on to the study procedures described below. In the event of any scheduling conflict, we may ask you to come and sign the consent form prior to your first visit. If this is the case, you will be compensated an additional $25 for your time and trouble.

Study Day 1: Glipizide challenge at the CRC: "Glipizide challenge" means that you will receive a dose of glipizide which is expected to lower your blood sugar.

Immediately after the screening procedures and during the same visit on Day 1, we will check a baseline fingerstick blood sugar to make sure you can take part in this study. If your first blood sugar is very close to the threshold, we may repeat it. "Baseline" means that this is an early test which will be compared to tests later in the study. The CRC nurse will place an intravenous catheter in a vein in your arm or hand. The intravenous catheter is a very thin, flexible tube which is inserted with a needle. The nurse will draw some blood for basic measurements. At the same time, a blood sample for DNA (gene analysis) will also be taken.

When your blood is drawn, you will also receive a single dose of glipizide (5 mg) to be taken by mouth. We will draw additional blood samples at 30, 60, 90, 120, 180 and 240 minutes. The total amount of blood will be about 11.3 tablespoons. Glucose measurements will also be taken every half an hour to make sure that your blood sugar does not drop too low.

If you develop symptoms of low blood sugar (sweating, nervousness, jitteriness, shakiness, confusion, blurred vision, lightheadedness, slurred speech, palpitations/fast heart rate, new onset of hunger or tiredness) another blood sugar measurement will be done. If any blood sugar is less than 50 mg/dl (with symptoms) or 45 mg/dl (without symptoms), or if nurses detect symptoms such as confusion, blurred vision and slurred speech, regardless of readings, the study will stop. If your blood sugar drops below 50 mg/dl, we will check it more often (every 5 minutes) even if you don't have symptoms. After this part of the study ends, you will be given juice and a sugar tablet. It will be important that you have a full breakfast immediately, which will be given to you at the CRC. If you continue to have symptoms after your full breakfast, we will check another blood sugar; in the very unlikely event that your blood sugar stays lower than 80 mg/dl, we will monitor you for a longer period. If you feel too uncomfortable, you are free to ask the nurse to stop the challenge at any time.

At the end of this visit, we will give you three 500 mg metformin pills to take home with instructions about how to take the drug. These instructions are also shown below.

Study Days 6-7: Short-term metformin treatment (at home)

You don't have to do anything special between the end of Study Day 1 and Day 6; you can continue your normal life. If your kidney function is adequate and your liver tests are not elevated as measured in the blood collected on the first visit, you will be asked by telephone or email to begin taking the first dose (1 pill) of metformin in the evening of Day 6. You will be asked to take the other two doses with breakfast (1 pill) and supper (1 pill) on Day 7. If you are taking diuretic medications (drugs that increase your urination, such as furosemide [Lasix] or hydrochlorothiazide [HCTZ]), your doses must be stable throughout this study. If they change, you should not take metformin. Once again, please limit your alcohol intake to one or fewer drinks on the evening of Day 7. One drink is defined as 12 ounces of beer, 5 ounces of wine or 1.5 ounces of liquor. You will then be asked to again fast overnight. As before, after midnight (the night of Study Day 7), you cannot have any food or drinks. Water is allowed and encouraged. If the first blood measurement shows that your kidney or liver function is not adequate to take metformin, or your diuretic doses change in the interim, you will be asked not to take metformin. However, you may continue with the procedures outlined below for Day 8 if you so desire.

Study Day 8: Oral glucose tolerance test on metformin at the CRC

On Study Day 8, you will be asked to have a study visit at the CRC after an overnight fast starting at midnight. As before, no food or drinks are allowed. Water is allowed. As before, we will do a baseline fingerstick blood sugar prior to administering any medication. If your blood sugar is above 250 mg/dl we will not continue the study for safety reasons, so as not to increase your blood sugar too much. We will give you the fourth dose of metformin (500 mg) and you will have another intravenous catheter placed. One hour later we will give you a drink containing 75g of sugar. Blood samples will be drawn before the drink, and again at 5, 10, 15, 30, 60 and 120 minutes. The total amount of blood drawn in this portion of this study will be about 8 tablespoons.

After the last blood sample, your part in the study will be complete, and you may go home.

Blood samples that we get from this study will be used to study the human response to the two drugs (glipizide and metformin), and how genes that influence risk of type 2 diabetes affect that response. All blood and DNA samples will be labeled with a number that does not identify you. The key linking this number to your identity will be kept separately in a password-protected file. The file will be kept in a locked file cabinet in a locked office. No one should be able to link any information that we get in this study to you. Also, this information will not be part of your medical record.

Samples that we get from this study will be frozen and stored for analyses of diabetes-related genes and molecules carried by the blood by Dr. Florez and his research collaborators. You can request that your samples be destroyed at any time by sending a letter to Dr. Florez at the Diabetes Unit, Massachusetts General Hospital, Boston, MA 02114. Because none of these genetic tests have an impact on medical practice currently, the results of the tests will not be told to you or to your primary care doctor. If some of the blood testing shows laboratory findings that may be relevant to your health and that your doctor may want to know about, for example that you may have pre-diabetes or diabetes, we will talk with you about this and suggest that you contact your primary care doctor so he/she can do the appropriate tests.

What are the risks and possible discomforts from being in this research study?

Expected risks and discomforts related to this study include:

1) low blood sugar,

2) catheter insertion, fingerstick measurements and blood drawing,

3) nausea, vomiting or diarrhea due to metformin,

4) stress from a possible diagnosis of diabetes, and

5) possible loss of confidentiality.

These are explained below:

1. During the glipizide challenge, your blood sugar is expected to drop. It may drop to the point of causing symptoms (sweating, nervousness, jitteriness, shakiness, confusion, blurred vision, lightheadedness, slurred speech, palpitations/fast heart rate). We will reduce this risk by asking you whether you have normal liver and kidney function and confirming this in your medical record. We will make sure that your starting blood sugar is above 80 mg/dl, and will give you a small dose of glipizide. However, we cannot guarantee that you will not experience these side effects.

   In order to reduce side effects from too severe a drop in blood sugar, we will also check blood measurements every half an hour, or at any time if you develop the symptoms described above. If you do, you should notify the study nurse. He/she will check your blood sugar, and the challenge will be stopped any time your blood sugar drops below 50 mg/dl (with symptoms) or 45 mg/dl (without symptoms). If your blood sugar drops below 50 mg/dl, we will check it more often (every 5 minutes) even if you don’t have symptoms. You

are free to withdraw from the study and stop the challenge at any time. Thus far in the study, approximately 25% of our participants have stopped the study early due to low blood sugar. All symptoms disappeared after sugar intake and a meal, which are provided to subjects at the end of the study. Metformin (taken in study days 6-8) should not lead to a drop in blood sugar.

2. Blood drawing will be done in very small amounts and spread out over one week, with about 11.3 tablespoons (167.5 cc) drawn during Visit 1, and about 8 tablespoons (118cc) drawn during Visit 2. An intravenous catheter will be placed in a vein in your arm or hand each day, in order to reduce the discomfort of repeated blood drawing. Occasionally, you may get a bruise. Rarely, a local skin reaction may also occur. Some people may experience syncope, or fainting, due to catheter insertion. The fingerstick to measure fasting sugar will also be slightly uncomfortable.

3. Metformin may occasionally cause loose stools or diarrhea. Metformin very rarely causes nausea and vomiting. In order to reduce these side effects, which occur in about 15 out of 100 subjects, we will use the lowest single dose available (500 mg) and limit metformin to four doses only. If you cannot tolerate the diarrhea, you may choose to notify us and stop taking metformin. You will have a simple oral glucose tolerance test on Day 8 instead.

4. Subjects should not have radiological studies that use contrast agents with iodine (such as special X-ray tests that use dye with iodine) within one week after taking metformin. The use of a dye with iodine can cause changes in the way your kidney works, and this might lead to serious side effects when taking Metformin.

5. If you have had previously undiagnosed diabetes, you may have results from the blood testing that suggest that you do have diabetes, or pre-diabetes. However, the design of this study prevents us from making a definite diagnosis. If your results suggest the presence of undiagnosed diabetes, or pre-diabetes, you will be asked to contact your primary care doctor. We will also notify your primary care doctor if you agree. No results will be entered into your medical record.

6. It is possible that a mistake could lead to a loss of confidentiality. Although the study doctors will make every effort to make sure that your private information is protected, a gap in security due to human error may make that information available to others. If lab results or genetic tests show that you have an increased risk of type 2 diabetes, this may lead to changes in your health insurance. Because we will do our best to make sure that none of the results obtained in this study become part of your medical record, and no result shared with

other investigators will contain any information that links the results to you, this risk is very small; however, we cannot guarantee that it does not exist.

Unexpected, but possible risks and discomforts related to this study include:

1) A convulsion or seizure from a very low blood sugar. The precautions taken above should make this risk extremely small, but may not make it disappear completely. We will have liquid sugar on hand which can be given by vein immediately if this occurs. People who have a seizure from a low blood sugar usually recover immediately after sugar is given.

2) An allergic reaction to any of the study drugs. Although we will check with you to make sure that you do not have a sulfa allergy, you may have an allergic reaction to any of the drugs we use. If you do, we will treat the allergic reaction with other drugs available to us.

There may be other risks or side effects that are not known at this time.

What are the possible benefits from being in this research study?

You are not expected to receive a medical benefit from taking part in this research study. The results of this study should help us understand how a person's genes affect the human response to commonly used drugs.

Can I still get medical care within Partners if I don't take part in this research study, or if I stop taking part?

Yes. Your decision won't change the medical care you get within Partners now or in the future. There will be no penalty, and you won't lose any benefits you receive now or have a right to receive.

Taking part in this research study is up to you. You can decide not to take part. If you decide to take part now, you can change your mind and drop out later. We will tell you if we learn new information that could make you change your mind about taking part in this research study.

If you take part in this research study, and want to drop out, you should tell us. We will make sure that you stop the study safely. We will also talk to you about follow-up care, if needed.

It is possible that we will have to ask you to drop out before you finish the study. If this happens, we will tell you why. We will also help arrange other care for you, if needed.

Will I be paid to take part in this research study?

You will receive $100 upon completion of both visits to the CRC. If, for some reason, you do not qualify for the second visit, you will receive $50 at the end of the first visit. You will also receive a free meal during your first visit to the CRC. You will be responsible for your own transportation. If during the screening part of the first visit you are found not to be a good candidate for this study, you will receive a $5 transportation voucher. You will receive reimbursement in the form of a check mailed within 4-6 weeks after you complete the study.

What will I have to pay for if I take part in this research study?

The study drug and all of the tests and procedures that will be done only for the research study will be paid for by study funds. The cost of your routine medical care will be billed to you or to your health insurance company in the usual way.

What if I am unable to complete the study?

If for some reason you have to drop out of the study or do not qualify for another reason, you may choose to remain in our contact list. In the future, we will contact you about any studies for which you may be eligible. If you elect not to be contacted we will properly dispose of your contact information.

☐ I **would** like to be contacted about future studies at Massachusetts General Hospital, Joslin Diabetes Center or their affiliates.

What happens if I am injured as a result of taking part in this research study?

We will offer you the care needed to treat any injury that directly results from taking part in this research study. We reserve the right to bill your insurance company or other third parties, if appropriate, for the care you get for the injury. We will try to have these costs paid for, but you may be responsible for some of them.

Giving you care does not mean that Partners hospitals or researchers are at fault, or that there was any wrongdoing. There are no plans for Partners to pay you or give you other compensation for the injury. However, you are not giving up any of your legal rights by signing this form.

If you think you have been injured or have experienced a medical problem as a result of taking part in this research study, tell the person in charge of this study as soon as possible. The researcher's name and phone number are listed in the next section of this consent form.

If I have questions or concerns about this research study, whom can I call?

You can call us with your questions or concerns. Our telephone numbers are listed below. Ask questions as often as you want.

Jose C. Florez, MD, PhD is the person in charge of this research study. You can call him at (617)-643-3308 Monday-Friday, 9:00 a.m. to 5:00 p.m. For urgent issues related to the study you can page Geoffrey Walford, MD at (617) 726-2241 x36833, 24 hours a day, 7 days a week.

If you have questions about the scheduling of appointments or study visits, call Amelia Lanier, B.A. at (617) 643-5419 or Marlene Fernandez, B.S. at (617) 643-5417 or email sugarmgh@partners.org.

If you want to speak with someone **not** directly involved in this research study, please contact the Partners Human Research Committee office. You can call them at 617-424-4100.

You can talk to them about:

- Your rights as a research subject
- Your concerns about the research
- A complaint about the research

Also, if you feel pressured to take part in this research study, or to continue with it, they want to know and can help.

If I take part in this research study, how will you protect my privacy?

Subject Identification

Federal law requires Partners (Partners HealthCare System and its hospitals, health care providers and researchers) to protect the privacy of health information that identifies you. This information is called Protected Health Information. In the rest of this section, we refer to this simply as "health information."

If you decide to take part in this research study, your health information may be used within Partners and may be shared with others outside of Partners, as explained below.

We have marked with a ☒ how we plan to use and share your health information. If a box is not checked ☐, it means that type of use or sharing is not planned for in this research study.

We will also give you the **Partners Notice for Use and Sharing of Protected Health Information.** The Notice gives more details about how we use and share your health information.

- **Health Information About You That Might be Used or Shared During This Research**

  ☒ Information from your hospital or office health records within Partners or elsewhere, that may be reasonably related to the conduct and oversight of the research study. If health information is needed from your doctors or hospitals outside Partners, you will be asked to give permission for these records to be sent to researchers within Partners.

  ☒ New health information from tests, procedures, visits, interviews, or forms filled out as part of this research study

- **Why Health Information About You Might be Used or Shared with Others**

  The reasons we might use or share your health information are:

  - To do the research described above
  - To make sure we do the research according to certain standards - standards set by ethics and law, and by quality groups
  - For public health and safety - for example, if we learn new health information that could mean harm to you or others, we may need to report this to a public health or a public safety authority
  - For treatment, payment, or health care operations

- **People and Groups That May Use or Share Your Health Information**

  1. People or groups within Partners

  ☒ Researchers and the staff involved in this research study

  ☒ The Partners review board that oversees the research

  ☒ Staff within Partners who need the information to do their jobs (such as billing, or for overseeing quality of care or research)

  2. People or groups outside Partners

  ☒ People or groups that we hire to do certain work for us, such as data storage companies, our insurers, or our lawyers

  ☒ Federal and state agencies (such as the U.S. Department of Health and Human Services, the Food and Drug Administration, the National Institutes of Health, and/or the Office for Human Research Protections) and other U.S. or foreign government bodies, if required by law or involved in overseeing the research

  ☒ Organizations that make sure hospital standards are met

  ☒ The sponsor(s) of the research study, and people or groups it hires to help perform this research study

  ☒ Other researchers and medical centers that are part of this research study

  ☒ A group that oversees the data (study information) and safety of this research study

  ☐ Other:

  Some people or groups who get your health information might not have to follow the same privacy rules that we follow. We share your health information only when we must, and we ask anyone who receives it from us to protect your privacy. However, once your information is shared outside Partners, we cannot promise that it will remain private.

- **Time Period During Which Your Health Information Might be Used or Shared With Others**

  - Because research is an ongoing process, we cannot give you an exact date when we will either destroy or stop using or sharing your health information.

- **Your Privacy Rights**

- You have the right **not** to sign this form permitting us to use and share your health information for research. If you don't sign this form, you can't take part in this research study. This is because we need to use the health information of everyone who takes part in this research study.
- You have the right to withdraw your permission for us to use or share your health information for this research study. If you want to withdraw your permission, you must notify the person in charge of this research study in writing.

  If you withdraw your permission, we will not be able to take back information that has already been used or shared with others. This includes information used or shared to carry out the research study or to be sure the research is safe and of high quality.

  If you withdraw your permission, you cannot continue to take part in this research study.
- You have the right to see and get a copy of your health information that is used or shared for treatment or for payment. To ask for this information, please contact the person in charge of this research study.

▪ **If Research Results Are Published or Used to Teach Others**

The results of this research study may be published in a medical book or journal, or used to teach others. However, your name or other identifying information **will not** be used for these purposes without your specific permission.

Consent/Assent to take part in this research study, and authorization to use or share your health information for research

Statement of Subject or Person Giving Consent/Assent

- I have read this consent form.
- This research study has been explained to me, including risks and possible benefits (if any), other options for treatments or procedures, and other important things about the study.
- I have had the opportunity to ask questions.

Subject Identification

If you understand the information we have given you, and would like to take part in this research study, and also agree to allow your health information to be used and shared as described above, then please sign below:

Signature of Subject:

_______________________________________ ______________
Adults <u>or</u> Minors, ages 14-17 Date/Time

OR

If you understand the information we have given you, and would like to give your permission for your child/the person you are authorized to represent to take part in this research study, and also agree to allow his/her health information to be used and shared as described above, then please sign below:

Signature of Parent(s)/Guardian or Authorized Representative:

_______________________________________ ______________
Parent(s)/Guardian of Minor Date/Time

OR

_______________________________________ ______________
Court-appointed Guardian or Health Care Proxy Date/Time

OR

_______________________________________ ______________
Family Member/Next-of-Kin Date/Time

Relationship to Subject:____

Signature of a Witness:

____ _________________
Witness (when required by the PHRC or sponsor) Date/Time

Statement of Study Doctor or Person Obtaining Consent

- I have explained the research to the study subject, and
- I have answered all questions about this research study to the best of my ability.

____ _________________
Study Doctor or Person Obtaining Consent Date/Time

In certain situations, the Partners Human Research Committee (PHRC) will require that a subject advocate also be involved in the consent process. The subject advocate is a person who looks out for the interests of the study subject. This person is not directly involved in carrying out the research. By signing below, the subject advocate represents (or "says") that the subject has given meaningful consent to take part in the research study.

Statement of Subject Advocate Witnessing the Consent Process

- I represent that the subject or authorized individual signing above has given meaningful consent.

____ _________________
Subject Advocate (when required by the PHRC or sponsor) Date/Time

Subject Identification

Consent Form Version Date: 11/23/11
