# Supplementary material for: The Study to Understand the Genetics of the Acute Response to Metformin and Glipizide in Humans (SUGAR-MGH): Design of a pharmacogenetic Resource for Type 2 Diabetes
Source: PLoS One. 2015 Mar 26;10(3):e0121553. doi: 10.1371/journal.pone.0121553 (PMC4374872; doi:10.1371/journal.pone.0121553)
Supplement: S2 Protocol — (DOCX) [file pone.0121553.s015.docx]

# STUDY TO UNDERSTAND THE GENETICS of the acute response

# to metformin AND GLIPIZIDE in humans (*SUGAR MGH*)

# Background and Significance

***I.1. Genetic associations with type 2 diabetes***

**The risk of type 2 diabetes is strongly influenced by inheritance (**[**1**](#_ENREF_1)**).** In contrast with type 1 diabetes, where a single locus (the *HLA* region) explains a large proportion of the observed heritability, the genetic architecture of type 2 diabetes appears to be comprised of several variants, each of which has a modest impact on disease risk ([2](#_ENREF_2)). Despite significant advances in our understanding of the genetic determinants of the monogenic forms of diabetes ([3](#_ENREF_3)), the definitive identification of genes that increase risk of common type 2 diabetes in the general population has been far more laborious.

**One such diabetes-associated gene is *PPARG*¸ which encodes the peroxisome proliferator-activated receptor γ (PPARγ), a target for thiazolidinedione medications.** A proline → alanine change in codon 12 of *PPARG*  (P12A) has been consistently associated with type 2 diabetes ([4-10](#_ENREF_4)), with the proline allele conferring a ~20% increased risk under a recessive model. Because of its high frequency in the population, the population attributable risk of this variant nears 25% ([5](#_ENREF_5)). How this molecular change impairs protein function and leads to an increased risk of type 2 diabetes has not been fully elucidated; similarly, no discernible effects of this variant on diabetes-related traits have been consistently documented.

**A second gene is *KCNJ11*, which encodes the islet ATP-sensitive potassium channel Kir6.2.** Severe activating mutations in *KCNJ11* cause a novel form of monogenic neonatal diabetes ([11](#_ENREF_11)). A common glutamate → lysine change at position 23 (E23K) has also been consistently associated with type 2 diabetes with an overall allelic odds ratio of 1.15 ([12-18](#_ENREF_12)); normoglycemic lysine carriers seem to have a defect in insulin secretion ([15](#_ENREF_15); [18](#_ENREF_18); [19](#_ENREF_19)). In vitro, the risk lysine allele seems to affect potassium channel properties ([20](#_ENREF_20); [21](#_ENREF_21)).

**Recently, a comparable level of robust statistical significance has been achieved for the association of common variants in the transcription factor 7-like 2 gene (*TCF7L2*) with type 2 diabetes (**[**22**](#_ENREF_22)**).** These deCODE investigators documented that a common microsatellite (DG10S478) was associated with type 2 diabetes in an Icelandic case/control sample (N=2,116), and replicated this result in two additional case/control Caucasian cohorts (N=1,658). The overall estimated allelic relative risk was 1.56, with a P value of 7.8 x 10^-15^ (after Bonferroni correction for the number of alleles tested). The non-coding single nucleotide polymorphisms (SNPs) rs12255372 and rs7903146 were in strong linkage disequilibrium with DG10S478 (r^2^=0.95 and 0.78, respectively), and showed comparably robust associations with type 2 diabetes.

**The evidence accumulated for the above variants is convincing and well established.** While the field of genetic association studies is plagued by instances of irreproducibility ([23-26](#_ENREF_23)), many publications that have examined *PPARG* P12A and *KCNJ11* E23K have reported consistent results, such that the cumulative *P* value for these variants exceeds 10^-10^ ([27](#_ENREF_27)) and unpublished observations). The same level of evidence is being gathered for *TCF7L2*, where we and others have replicated this result in every population examined (see Section I.3 below).

**Since 2007 many more polymorphisms have been associated with type 2 diabetes and related traits.** The pace of discovery changed dramatically with the advent of genome-wide association studies (GWAS) ([28](#_ENREF_28)). The identification of millions of SNPs and their deposition in public databases; the manufacturing of genotyping arrays that could simultaneously query hundreds of thousands of SNPs with great precision; the understanding of an underlying correlation structure between SNPs, driven by the finite number of recombination events in human history, which reduced the complexity of the variation to be interrogated; the recognition that the scientific imperative of reproducibility required the acceptance of strict statistical thresholds that accounted for the universe of possible hypotheses in the human genome; and the corollary of such awareness, that for these very small *P* values to be achieved very large sample sizes had to be assembled through international collaboration, all coalesced to enable the pursuit of GWAS, where most of the common variants in the human genome (those with a minor allele frequency >5%) could be tested in one fell swoop. Several independent GWAS ([29-33](#_ENREF_29)) and the growing scientific exchange that led to successive meta-analyses of ever-increasing size ([34](#_ENREF_34); [35](#_ENREF_35)) soon produced a plethora of robust associations, such that the landscape of type 2 diabetes-associated variants grew from three prior to the GWAS era to several dozen in just a few years ([36](#_ENREF_36)). This list has been complemented by the implementation of similar approaches in the discovery of genetic determinants of quantitative glycemic traits ([37-42](#_ENREF_37)), the extension of GWAS to non-European populations ([43-48](#_ENREF_43)), and the deployment of custom-made arrays that allow for the rapid and efficient genotyping of top signals across thousands of additional samples ([49](#_ENREF_49); [50](#_ENREF_50)).

***I.2. Heterogeneity in pharmacological response***

**Not much is known about the basis of variable response to therapy in type 2 diabetes.** The long-recognized heterogeneity in patient response to treatment has not been studied systematically, such that in clinical practice diabetologists often choose first-line agents based on their side effect profile or known contraindications. Thus, although good reasons to select a particular agent exist (e.g. metformin to induce weight loss, a thiazolidinedione where insulin resistance is suspected), therapeutic decisions are made on a population basis, rather than being informed by more detailed knowledge of the particular characteristics of type 2 diabetes in each patient.

**As an example, preliminary evidence suggests the existence of a group of non-responders to thiazolidinedione medications.** Suter *et al.* studied the effects of troglitazone 400 mg daily in 11 subjects with type 2 diabetes, and found that 3/11 did not lower their fasting plasma glucose after a 6-12-week period of treatment; the change in fasting plasma glucose appeared to predict performance in an OGTT, a meal tolerance test and both glucose disposal and hepatic glucose production during a glucose-clamp study ([51](#_ENREF_51)). In a different study, 1/7 patients with IGT failed to show improvement in glucose tolerance after 12 weeks of troglitazone treatment (400 mg daily), 2/12 did not increase their glucose disposal rates after treatment, and 2/12 did not increase their insulin sensitivity index after treatment ([52](#_ENREF_52)). Similarly, 9/63 patients on 2 mg of rosiglitazone daily and 5/83 patients on 4 mg of rosiglitazone daily for 26 weeks had an increase in their HbA_1c_ at the end of the treatment period, rather than the expected decrease ([53](#_ENREF_53)). Finally, among Hispanic women with a history of gestational diabetes enrolled in the In the Troglitazone In the Prevention Of Diabetes (TRIPOD) study, 30% of women treated with troglitazone gained no protection from type 2 diabetes when compared with the placebo group, an effect attributed to their lack of improvement in insulin sensitivity ([54](#_ENREF_54)).

**Variability to drug response may have a genetic component.** For example, glyburide is metabolized by cytochrome P450 (*CYP2C9*), which has been found to contain two nonsynonymous polymorphisms (R144C and I359L) which affect its enzymatic activity and alter the clearance of glyburide ([55](#_ENREF_55)), ([56](#_ENREF_56)). Recently, Wolford *et al.* have shown that genetic variants in *PPARG* (other than P12A) may underlie the differential response to troglitazone shown in the TRIPOD participants ([57](#_ENREF_57)). Similarly, Sesti and colleagues recently reported a higher proportion of lysine carriers among subjects who failed sulfonylurea-metformin combined therapy (defined as a rise in fasting plasma glucose above 300 mg/dl); interestingly, islets isolated from lysine carriers showed a diminished insulin response to glibenclamide ([58](#_ENREF_58)).

**Despite the convincing associations of several genetic variants with type 2 diabetes and their involvement in physiological pathways involved in drug response, their impact on pharmacological interventions has not been systematically examined.** The completion of the Human Genome Project ([59](#_ENREF_59); [60](#_ENREF_60)) and the high-density characterization of common human variation in four different ethnic groups ([61](#_ENREF_61)) highlight the promise of genomic medicine. The elucidation of the genetic architecture of complex phenotypes may help clinicians understand disease heterogeneity, uncover new pathophysiological mechanisms, open the opportunity for novel therapeutic interventions, provide predictive diagnostic and prognostic information, and allow for individually tailored therapy that takes into account both the probability of response and the incidence of drug-induced complications ([62](#_ENREF_62)). *Our line of work, as elaborated in published reports, ongoing studies (see preliminary data below) and planned projects such as the Research Plan that follows, aims to contribute in the filling of this crucial translational gap.*

**Data from basic science and epidemiological studies support a link between glucose metabolism and the dietary intake of nutritents such as zinc, vitamin D, and calcium.** With regards to zinc intake, one might assume that impaired zinc transport in the β cell conferred by genetic variation may be attenuated by dietary zinc or zinc supplementation. Five genome-wide association scans for type 2 diabetes have confirmed a known type 2 diabetes loci at a missense single nucleotide polymporphism, rs13266634, in the gene SLC30A8 which encodes a β-cell zinc transporter ZnT-8 ([29-33](#_ENREF_29)). Prior studies have shown improvement in insulin sensitivity with zinc supplementation and epidemiologic studies have shown an association between higher dietary zinc and diabetes prevalence ([63](#_ENREF_63); [64](#_ENREF_64)). Given that zinc transport is essential for insulin secretion from the β cell, it could be hypothesized that high dietary zinc may not only lessen the risk of type 2 diabetes, but furthermore, accentuate the effect of sulfonureas on insulin secretion in the presence of certain risk SNPs, such as rs13266634. From the perspective of calcium and vitamin, the vitamin D receptor is expressed in β cells and the human insulin receptor gene has a vitamin D response element ([65](#_ENREF_65)). An NHANES study showed an inverse association between higher vitamin D status and type 2 diabetes in non-Hispanic whites and Mexican Americans ([66](#_ENREF_66)). The Nurses Health Study demonstrated lower risk of t2DM in individuals with higher calcium and vitamin D intake ([67](#_ENREF_67)). In order to further support vitamin D’s role in glucose metabolism, a recent study established that vitamin D and calcium supplementation improved fasting glucose and insulin resistance in subjects with impaired fasting glucose ([68](#_ENREF_68)). There has been conflicting data regarding the influence of Vitamin-D genetic variants on glycemic traits and type 2 diabetes incidence. Some studies have illustrated that Vitamin-D genetic variants are associated with insulin resistance. Given this interesting data, one might imagine that dietary vitamin D and vitamin D-associated genetic variants may not only influence insulin resistance, but possibly potentiate the glucose lower effects of metformin. *By obtaining dietary data from study participants,* o*ur proposed study hopes to elucidate the link between these dietary nutrients and associated genetic variants with glycemic traits and response to anti-diabetic medications.*

# *I.3. Preliminary Data*

**I.3.a. Studies on candidate genes that encode drug targets in type 2 diabetes**

**We have conducted well-powered, comprehensive genetic association studies designed to establish whether common variation in genes that encode drug targets are associated with type 2 diabetes.** By combining a well-phenotyped, large patient collection with a comprehensive approach to capture all common genetic variation in a given genomic region, we have been able to systematically pursue comprehensive association studies in candidate genes of high interest. Thus, we have completed the entire common haplotype structure of the genes encoding the sulfonylurea receptor (*ABCC8*) and its associated potassium channel (*KCNJ11*) ([18](#_ENREF_18)), the protein tyrosine phosphatase 1B (*PTPN1*) ([69](#_ENREF_69)), the insulin degrading enzyme (*IDE*) ([70](#_ENREF_70)), the seven AMP kinase genes ([71](#_ENREF_71)) and *TCF7L2* ([72](#_ENREF_72)). We have confirmed the published associations of the E23K variant of *KCNJ11* and rs7903146 in *TCF7L2* with type 2 diabetes ([18](#_ENREF_18); [72](#_ENREF_72); [73](#_ENREF_73)), and demonstrated that the K allele at *KCNJ11* E23K leads to decreased insulin secretion in normoglycemic subjects (Fig. 1).

Fig. 1: Differences in insulin secretion (as measured by the insulinogenic index) in normoglycemic individuals, depending on genotype at *KCNJ11* E23K (*P*<0.02).

**In addition to the comprehensive evaluation of common variation in candidate genes, we have tested specific genetic variants examined by other groups.** Through our long-standing collaborations with Drs. Leif Group, Tom Hudson and Kristin Ardlie, we have gained access to large patient collections that comprise both case/control and family-based samples. These samples have adequate power to reproduce previously published associations under specific genetic models, and their family-based components are robust to population stratification. While these samples have confirmed results widely observed by other groups, such as *PPARG* P12A ([5](#_ENREF_5)), *KCNJ11* E23K ([18](#_ENREF_18); [73](#_ENREF_73)) and *TCF7L2* (([72](#_ENREF_72)), we have been unable to replicate published associations in the genes that encode the insulin receptor substrate 1 (*IRS1*) ([73](#_ENREF_73)) and the ectoenzyme nucleotide pyrophosphate phosphodiesterase 1 *(ENPP1*) ([74](#_ENREF_74)), as well as *PTPN1* ([69](#_ENREF_69)) and *IDE* ([70](#_ENREF_70)).

**I.3.b. Prospective evaluation of associated polymorphisms**


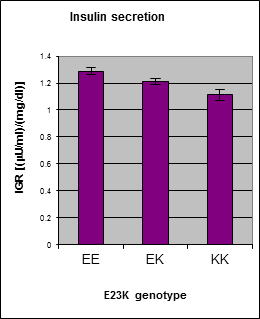


P < 0.01

**We have examined these validated associations prospectively**. As part of the second Specific Aim in the PI’s K23 Research Career Development Award, and under the mentorship of Dr. David Nathan, we have collaborated with the Diabetes Prevention Program (DPP) in testing whether specific genetic variants predict the development of diabetes in a multi-ethnic population of subjects with IGT and affect their response to preventive interventions. Consistent with prior cross-sectional studies, homozygotes for the proline allele in *PPARG* P12A progressed more rapidly from IGT to diabetes than alanine carriers (HR 1.24, 95% CI 0.99-1.57, *P*=0.07), with no interaction between genotype and intervention. There was a significant interaction of genotype x BMI, with a stronger relationship between BMI and progression to diabetes in alanine carriers. We detected no statistically significant effect of genotype on quantitative glycemic traits at baseline, nor on troglitazone response at one year (Florez *et al.,* in preparation).

Fig. 2: Effects of *KCNJ11* E23K genotype on baseline insulin to glucose ratio (IGR) in the Diabetes Prevention Program.

**The effect of *KCNJ11* E23K on development of diabetes and on related traits was more complex.** As previously reported in normal subjects, lysine carriers at *KCNJ11* E23K had reduced insulin secretion at baseline (Fig. 2). Nevertheless, EK heterozygotes were *less* likely to develop diabetes than EE homozygotes (HR 0.70, 95% CI 0.54-0.91, *P*<0.01), in a direction consistent with a large prospective study ([19](#_ENREF_19)). There was a novel interaction of metformin with E23K genotype (*P*=0.02): lysine carriers displayed a diminished preventive effect of metformin (HR 0.89 [95% CI 0.66-1.19] for EK and 0.95 [95% CI 0.54-1.67] for KK vs placebo), while EE homozygotes had a greater preventive effect (HR 0.55 [95% CI 0.42-0.71]).

**We have also replicated the association of *TCF7L2* with type 2 diabetes.** Over an average of 3 years, DPP participants with the risk TT genotype at rs7903146 were more likely to progress from IGT to diabetes than GG homozygotes (HR 1.55, 95% CI 1.20-2.01, *P*<0.001). The effect of genotype was stronger in the placebo group (HR 1.81, 95% CI 1.21-2.70, *P*<0.01) than in the metformin and lifestyle intervention groups (HR 1.62 and 1.15, respectively), although the genotype x intervention interaction was not statistically significant. Genotype was not associated with the response of these measures to intervention at one year. Similar results were obtained for rs12255372 ([75](#_ENREF_75)).

**I.3.c. Effects on quantitative glycemic traits**

**As mentioned above, *KCNJ11* E23K affects insulin secretion in normoglycemic individuals and in persons with IGT.** We ([18](#_ENREF_18)) and others ([15](#_ENREF_15); [19](#_ENREF_19)) have shown that the K allele leads to diminished insulin secretion during the initial phase of an OGTT in normoglycemic Caucasian individuals (Fig. 1); this effect is also seen in the multi-ethnic DPP cohort of individuals with IGT (Fig. 2) ([76](#_ENREF_76)).

**The lack of a preventive response to metformin noted in DPP participants with the KK genotype at *KCNJ11* E23K is correlated with a lack of improvement in insulin sensitivity.** While metformin seems to protect EE homozygotes at *KCNJ11* E23K, it does not delay or prevent the onset of diabetes in K allele carriers (see above). This lack of response may be explained by the obliteration of any improvement in insulin sensitivity observed in KK homozygotes, in contrast to the improvements noted in the lifestyle intervention and troglitazone arms (Fig. 3).


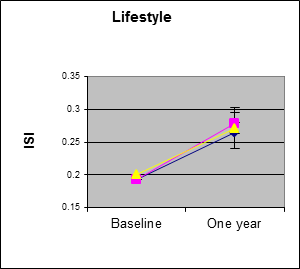

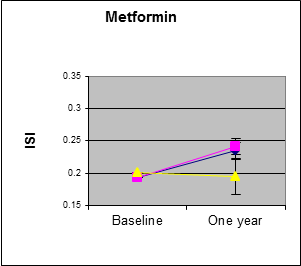

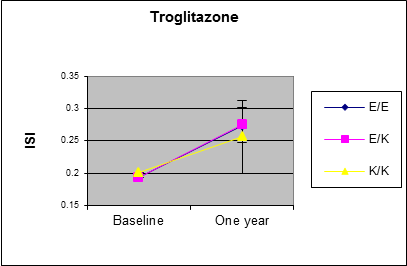


**Variants in *TCF7L2* also impair insulin secretion.** We have demonstrated lower insulinogenic index values both in normoglycemic ([72](#_ENREF_72)) and IGT ([75](#_ENREF_75)) individuals who carry the risk TT genotype at *TCF7L2* rs7901346 when compared to CC homozygotes (in control individuals, the insulinogenic index was 0.61 ± 0.71 vs 1.00 ± 1.84, *P* <0.001; mean ± SD). This transcription factor is postulated to influence expression of GLP-1 ([22](#_ENREF_22)), an enteroendocrine incretin which activates insulin secretion after a meal ([77](#_ENREF_77)). Thus, while a glucose oral load might be expected to elicit different insulin secretory responses depending on variation at *TCF7L2*, bypassing GLP-1 by intervening at a more distal step in the insulin secretion pathway should eliminate the immediate genetic consequences of *TCF7L2* variants. Whether GLP-1 levels are actually different according to *TCF7L2* genotype has not been measured directly.

Fig. 3: Insulin sensitivity Index (ISI) according to *KCNJ11* E23K genotype one year after a lifestyle intervention, metformin or troglitazone. ISI does not improve in KK homozygotes after metformin treatment (*P*<0.01).

**This proposal intends to validate and extend these observations.** While *KCNJ11* E23K is known to impair insulin secretion, whether allelic variation at this locus affects the acute response to sulfonylurea treatment in vivo has not been determined. In addition, the proposed mechanism of action of *TCF7L2* in regulating insulin secretion via GLP-1 (or glucagon) has not been tested. Finally, it is not clear whether the unexpected lack of improvement in insulin sensitivity in individuals with the KK genotype at *KCNJ11* after metformin treatment is an acute or chronic effect, or merely a statistical fluctuation. *The pilot studies proposed here intend to answer these questions and set the stage for an outcomes-based clinical trial that extends these pharmacogenetic findings into the clinical arena.*

# Statement of Hypothesis and Specific Aims

**Given these preliminary findings, we hypothesize that variants in genes that are reproducibly associated with type 2 diabetes or related traits may impact the effect of anti-diabetic medications.** In particular, sulfonylureas may have differential effects on individuals depending on the allelic variant they carry at *KCNJ11* E23K; conversely, because *TCF7L2* is postulated to influence insulin secretion by regulating levels of glucagon-like peptide 1 (GLP-1), and sulfonylureas act at a more distal step in the insulin secretion pathway, the effect of sulfonylureas on insulin secretion should be independent of genetic variation at *TCF7L2*. Finally, it is not known whether the effect of metformin on insulin sensitivity in KK homozygotes at *KCNJ11* occurs in the acute setting or only after long-term treatment.

**We therefore propose the following Specific Aims:**

Specific Aim No.1: To examine the acute response to a sulfonylurea challenge (glipizide 5 mg orally) in subjects at risk of diabetes or with early diabetes (on diet treatment alone), depending on genotype at *KCNJ11* E23K, *TCF7L2* rs7903146, or newly discovered variants linked to type 2 diabetes via associations with related phenotypes, individually or in aggregate

- We hypothesize that subjects with a subset of variants will have an attenuated response, whereas those with a different subset will have no discernible impact on this response

Specific Aim No. 2: To examine the acute response to short-term metformin treatment on the insulin sensitivity index in the same group, depending on genotype at *KCNJ11* E23K, or newly discovered variants linked to type 2 diabetes via associations with related phenotypes, individually or in aggregate

- We hypothesize that subjects with a subset of variants will have a diminished improvement in insulin sensitivity compared to non-carriers

Specific Aim No. 3: To examine acute insulin secretion (by the insulinogenic index derived from an OGTT), GLP-1 and glucacon levels, and metabolomic profiling after short-term metformin treatment (500 mg bid x 4 doses) in the same group of subjects, depending on genotype at *TCF7L2* rs7903146, or newly discovered variants linked to type 2 diabetes via associations with related phenotypes, individually or in aggregate

- We hypothesize that subjects with the risk TT genotype at *TCF7L2* rs7903146, or a subset of variants will have a reduced insulinogenic index and GLP-1 levels than non-carriers

***If successful, this proposal should help clarify the pathophysiologic mechanisms by which these key genetic variants increase risk of type 2 diabetes, and assess their impact on commonly used antidiabetic treatments. In addition, this pilot study will lay the groundwork for a long-term, outcomes-based pharmacogenetic clinical trial.***

# Subject Selection

***III.1. Available samples***

**Our goal is to recruit research subjects likely to require antidiabetic medications in their lifetime.** These include individuals with early type 2 diabetes (on diet treatment alone), or at higher-than-average risk of developing diabetes (impaired fasting glucose, IGT, a history of gestational diabetes, or suffering from the metabolic syndrome, obesity and/or the polycystic ovary syndrome [PCOS]). We have assembled a local team of collaborators with a shared interest in diabetes and metabolic traits, who have ready access to suitable research subjects.

**Our main target for recruitment will be the local Partners Network practices.** Making use of the electronic medical record available to all 12 MGH-based primary care practices, Dr. Richard Grant has compiled a Practice-Based Research Network (PBRN), based on the approximately 90,000 primary care patients with regular care at MGH (i.e. an identified MGH primary care physician *and* a clinic visit in the prior 2 years). This database includes a cohort of 7,692 patients with type 2 diabetes seen between 07/01/04 and 06/30/05. Of those, there were 1,547 patients (20.1%) not on glycemic medication therapy (i.e. diet/lifestyle); their characteristics are shown in Table 1.

| Table 1: Demographic characteristics of diet-treated MGH patients with diabetes | |
| --- | --- |
| Female | 775 (50.1%) |
| Age (years) | 65.3 ± 14.7 |
| Caucasian | 1,202 (77.7%) |
| Current smoker | 230 (14.9%) |
| Coronary artery disease | 382 (24.7%) |
| BMI (kg/m^2^) | 31 ± 7.6 |
| N medications | 7.9 ± 5.2 |
| Clinic visits previous year | 7.6 ± 6.2 |
| HbA_1C_ (%) | 6.7 ± 1.1 |
| Mean blood pressure (mm Hg) | 130/74 |
| LDL cholesterol (mg/dl) | 93.7 ± 32.5 |
| Variables are expressed as n (%) or mean ± SD. | |

**A second possible source of volunteers will be non-diabetic subjects with at least one elevated random glucose (>200 mg/dl).** In addition to the above diet-treated patients with diabetes, Dr. Grant has identified another ~1,500 MGH primary care patients who do not carry the diagnosis of diabetes, but have at least one random glucose >200 mg/dl in the medical record. Because hyperglycemia is predictive of future diabetes ([78](#_ENREF_78); [79](#_ENREF_79)), this group is at higher-than-average risk of ever requiring antidiabetic drugs.

**Other Partners and Joslin collaborators work with susceptible patient populations.** Dr. Corrine Welt at the MGH Reproductive Endocrine Unit is currently carrying out a study with about 500 patients with PCOS, only a small fraction of whom (~10%) are on current metformin treatment. Drs. Steven Grinspoon and Janet Lo have an established research program on the metabolic syndrome which comprises approximately 100 subjects with and without HIV infection. Dr. Ravi Thadhani has been following a cohort of ~200 women with a history of gestational diabetes mellitus (GDM) ([80](#_ENREF_80)). Finally, we have access to 1,004 non-diabetic subjects formerly enrolled in a Partners-Roche Consortium who have consented to diabetes-related genetic investigation, 25% of whom have BMI >30 kg/m^2^. These cohorts can be further expanded, if necessary, with additional help from the MGH and Brigham Obesity Clinics. At the Joslin Diabetes Center, family members of the diabetic patients will also be recruited.

As the study aims to test the effect of specific genetic variants on pharmacological response, recruitment efforts will also include queries within an institutional biorepository containing samples adequate for genotyping and demographic information on individuals interested in participating in clinical trials. Individuals who have already indicated interest in participating in future studies and given consent for genotype-directed call back into future research studies will be contacted first by mail to assess their interest in participating in SUGARMGH and then, unless otherwise indicated, by phone.

In the event that an individual does not qualify to participate in the study, we will ask if they would like to be contacted by collaborators involved in other research studies.

In the event that recruitment lags, we will advertise outside of the Partners and Joslin networks. This may include posting flyers, internet advertising, newspaper ads and any other forms of displaying the approved flyer.

***III.2. Eligibility criteria***

**As stated above, we intend to enroll research subjects at higher-than-average risk of requiring antidiabetic medications in their lifetime.** Keeping in mind the possible side effects of the study drugs (glipizide and metformin), we will adhere to the following inclusion and exclusion criteria:

Inclusion criteria:

- Male or non-pregnant female > 18 years of age
- We will target preferentially people at risk of diabetes or requiring diabetes meds
  - The first tier of risk will be illustrated by one of the following variables (e.g. established type 2 diabetes on diet therapy alone, elevated random glucose in electronic medical record, PCOS, metabolic syndrome, obesity, history of gestational diabetes, etc.)
  - The second tier of risk will be illustrated by other features that correlate with diabetes risk, such as a history of hypertension or dyslipidemia
- Otherwise healthy subjects may also be candidates for the study.
- Able and willing to give consent relevant to genetic investigation

Exclusion criteria:

- Pregnant, nursing or at risk of becoming pregnant
- Currently taking any medications for the treatment of diabetes
- Currently on metformin for any other indication (e.g. PCOS)
- Onset of diabetes before age 25, with autosomal transmission of diabetes across three generations
- History of liver or kidney disease
- Known severe allergic reactions to sulfonamides
- History of porphyria
- Documented estimated glomerular filtration rate (GFR) < 60 ml/min/1.73 m^2^, based on the most recent serum creatinine measurement available in the electronic medical record, and calculated by the Modification of Diet in Renal Disease equation ([81](#_ENREF_81)) available at <http://www.nephron.com/cgi-bin/MDRD_GFR.cgi>
- Currently taking medications known to affect glycemic parameters, such as glucocorticoids, growth hormone or fluoroquinolones
- Planned radiologic or angiographic study requiring contrast within one week of completion of this study
- Established coronary artery disease (CAD), defined as:
  1. History of myocardial infarction.
  2. History of revascularization (coronary artery bypass grafting, percutaneous coronary intervention (e.g. stenting or balloon angioplasty).
  3. Evidence of ischemia on cardiac stress test.
- Enrolled in any other interventional study at time of screening through completion of study protocol
- History of bariatric surgery
- History of seizures
- History of stroke/CVA

1. **Subject Enrollment**

**Eligible patients will be identified by review of electronic data queried from the central data repositories (labs, visits, billing diagnoses) and electronic medical records (problem lists, medications, diagnoses) using previously validated methods.** Some subjects will also be identified from lists of participants from previous research studies. Primary care physicians will be given a single list of their own patients with the option to exclude patients deemed inappropriate for contact. A letter co-signed by the patient’s primary care physician and the study PI will then be sent to each subject explaining the general idea for the study in simple terms, with a stamped postcard and phone number providing an option to either “opt-in” or “opt-out”. In the event that the subject has been recommended by a collaborator, previously participated in a research study at a Partners network site, and gave consent to be contacted in the future; we will substitute their PCP signature with that of the recommending study PI. If the potential research subject has not declined further contact in 2 weeks, the study staff may call him/her and invite him or her to participate in the study. Prior experience with this approach by our group has yielded a 15% initial decline rate and 75% final calculated response rate. Subjects who agree to participation will be further screened via telephone with a brief questionnaire, sent further information and an informed consent form in the mail, and invited to come to the first visit. In addition, flyers will be posted in the internal medicine clinics and the study will be advertised via Partners email. Materials will be prepared both in English and in Spanish; for subjects who request another language arrangements will be made with a translation service.

1. **Study Procedures**

***V.1. Visit 1: Screening***

**On Day 1, the research subject will present to the clinical research center after an overnight fast.** He/she will have had the opportunity to read the consent form ahead of time (see below). All questions will be answered, signed informed consent will be obtained and a brief history will be taken in order to verify inclusion and exclusion criteria. During the consenting process, the subjects will be informed on how to fill out a four day food record. On occasion, if it is convenient for the subject, signed informed consent may be obtained prior to Day 1. Women of childbearing age who are currently sexually active and not using birth control will receive a urine pregnancy test. Vital signs and anthropometric measurements (height and weight) will be obtained by a nurse. Eligible and willing subjects will then proceed to the sulfonylurea challenge as part of this initial visit.


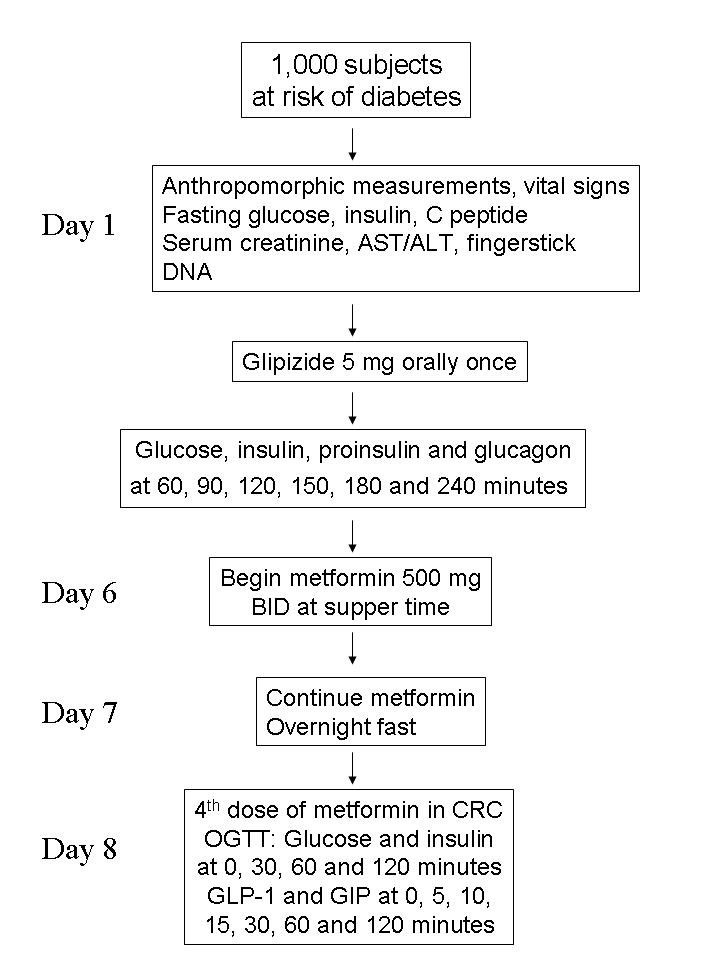

***V.2. Day 1: Sulfonylurea challenge***

**In order to prevent significant hypoglycemia, a baseline fingerstick >80 mg/dl will be required.**

If the blood glucose is between 70 mg/dL and 80 mg/dL, and the subject has arrived before 8am, *or* the patient is very interested in proceeding with the study, he or she will be offered the opportunity to have a second blood glucose drawn at least 30 mins after the initial check. If the second glucose is above 80 mg/dL, proceed with the standard study protocol. If the subject does not wish to proceed, or if the fasting glucose is below 80 mg/dL, only baseline blood will be drawn. The subject will still be invited to come back for Visit 2 (Day 8).

Subjects will then have a 20G intravenous catheter placed, and baseline (time 0) serum insulin, metabolomic profiling, C peptide and glucose will be obtained. In addition, a comprehensive metabolic panel and an extra 20 cc tube of whole blood for DNA extraction will also be drawn. They will then receive a single oral dose of glipizide 5 mg, and simultaneous insulin and glucose will be obtained at 30, 60, 90, 120, 180 and 240 minutes. Additionally, metabolomic profiling samples will be drawn at 60 and 120 minutes. Routine glucose measurements (for subject safety) will also be obtained every half an hour from intravenous blood. The frequency of blood glucose measurements will increase to every 5 minutes for an asymptomatic glucose <50 mg/dl. If the subject develops symptoms of hypoglycemia (diaphoresis, lightheadedness, confusion, anxiety, new onset of hunger or tiredness) another glucose measurement will be obtained: 1. If glucose > 50 mg/dl, glucose measurements will increase to every 10 minutes. If subject no longer feels symptoms of hypoglycemia, the nurse may return to routine checks after 3 documented rising or stable blood sugars. 2. If any glucose measurement is <50 mg/dl (with symptoms) or 45 mg/dl (without symptoms) or if nurses detect symptoms such as confusion, blurred vision and slurred speech, regardless of glucose measurements, the challenge will be aborted and the patient will be given juice and a glucotab, and advised to break his/her fast immediately. If the subject cannot swallow, 1 amp of D50 will be administered by IV. At the conclusion of the sulfonylurea challenge, every subject will be fed a provided meal rich in carbohydrate content. Any subject with persistent symptoms of hypoglycemia after the full meal will have another glucose measurement; persons with a glucose <80 mg/dl will be monitored for another 3 hours at the clinical research center, or longer if deemed necessary for their safety by the PI.

Before discharge, the patient will receive three 500 mg metformin pills to take home with the appropriate instructions. If subject suffered symptoms of hypoglycemia, the nurse will provide them with an educational pamphlet on monitoring blood sugar as well as a snack to eat at home. Days 1-6 will constitute the washout period for the single administered dose of glipizide (~12 half-lives by a conservative estimation).

**V.3**. **Days 2-7: Food record**

**The participants will fill out a dietary intake food record.** The food record will span 3 weekdays and one weekend day. They will use a standardized food record provided by the MGH Bionutrition/Metabolic Phenotyping Core. They will be asked to bring this detailed food record to Visit 2.

***V.4. Days 6-7: Short-term metformin treatment***

**The baseline serum creatinine will be used to recalculate GFR.** If the new estimated GFR is < 60 ml/min/1.73 m^2^ based on the MDRD equation, the subject will undergo a simple OGTT on Day 8 in the absence of any metformin treatment. In addition, we will also ensure that for subjects who are taking a diuretic medication, their dose remains stable between Visits 1 and 2; if it doesn’t, the patient will be asked to refrain from taking metformin and will present for a simple OGTT. Likewise, we will check ALT and AST levels from the baseline comprehensive metabolic panel, and if either one is 3x higher than the upper limit of normal the subject will be asked not to take the metformin and instead report for a simple OGTT. Otherwise, he/she will be asked by telephone or email to begin taking the first dose of metformin in the evening of Day 6, and to take the other two doses with breakfast and supper on Day 7. The subject will then be asked to keep another overnight fast.

Should the subject need to reschedule his/her second visit due to an unforeseen circumstance, and he/she took one or more metformin at home, arrangements will be made to supply another course of metformin to him/her before his/her rescheduled second visit.

***V.5. Visit 2: OGTT on metformin***

**On Day 8, the research subject will again present to the clinical research center after an overnight fast.** A baseline fingerstick will be taken and if the fasting blood sugar is <60 mg/dl, the subject will receive the metformin ten minutes after the Glucola administration instead of an hour prior**.** If fasting blood sugar is >250 mg/dl subject will be excluded and study terminated. As long as the fingerstick glucose is >60 mg/dl and <250 mg/dl, the subject will receive the 4^th^ dose of metformin 500 mg, and have a 20G intravenous catheter placed; one hour later a 75g OGTT will commence. Insulin and glucose will be obtained at 0, 30, 60 and 120 minutes, and a separate tube will be frozen for GLP-1 determination at each time point (in addition, GLP-1 tubes will also be obtained at 5, 10 and 15 minutes; leftover serum will be frozen for future metabolomic profiling). Additionally, metabolomic profiling samples will be drawn at 0, 60 and 120 minutes. After the last dose of metformin has been administered, the subject will review his/her food record with an MGH diet technician. If he/she forgets to bring the food record, they will be provided with a stamped envelope to mail a copy of the food record to the study coordinators and the study coordinators will review the food records by phone.

At the conclusion of the OGTT the subject will be dismissed and the study concluded. A schematic version of the protocol for medication administration and blood draws is shown in Fig. 4.

***V.6. Choice of drugs and doses***

**Glipizide** was chosen among sulfonylureas because it has great oral bioavailability, immediate onset (initial response at 30 minutes, peak response at 2-3 hours), relatively short half-life (2-8 hours, 10 hours in the elderly) and no need of adjustment for renal insufficiency or age. The chosen dose (5 mg) is the initial dose commonly used in type 2 diabetes, and represents a reasonable compromise between achieving an effect of sufficient magnitude while avoiding severe hypoglycemia. Severe hypoglycemia requiring assistance occurs 0.19-2.5 episodes per 1000 patient years. **Metformin** is the only drug available in its class, and was chosen to replicate and extend the findings observed in the DPP. We have elected submaximal doses to avoid gastrointestinal side effects and ensure short-term adherence to the protocol. Metformin will not be administered to individuals with renal dysfunction.

***V.7. Subject confidentiality***

**At all moments, the privacy of research subjects will be protected.** Subjects will receive a coded, anonymous numerical identifier that will link their anthropomorphic, biochemical and genetic measurements to each other but not to the individual. The key will be stored in the PI’s office in a locked cabinet and in his password-protected hard drive. A code will also be used to mark his/her self-reported ethnic group. If the OGTT suggests the presence of diabetes in a previously undiagnosed subject, the subject (and his/her primary care physician if agreed beforehand) will be contacted for appropriate follow-up and confirmation.

***V.8. Laboratory procedures***

**V.8.a. Biochemical measurements**

**The local assay laboratory will perform all biochemical measurements of glucose, insulin, C peptide and serum creatinine.** Urine HCG pregnancy tests and fingersticks will be carried out in the clinical research center by the study nurse. Separate tubes for DNA (Visit 1) and GLP-1 (Visit 2) will be frozen and stored in the PI’s laboratory in a -80^o^C freezer. All tubes will be labeled with a coded, anonymous identifier.

**V.8.b. DNA extraction and genotyping**

**DNA will be extracted with a standard DNA isolation kit.** The Puregene alcohol precipitation kit from Gentra systems will be used to extract DNA from 10cc of whole blood (the other 10cc will be kept frozen as a backup), expected to yield 150-300 μg of DNA. DNA will be quantified by Picogreen analysis and plated onto 96-well storage plates, from which 384-well working plates will be prepared. Each plate will have a unique configuration of empty wells so as to be able to detect plate misassignment, and will also include a number of duplicate samples. The gender of each sample will be verified by genotyping the sex-specific AMELXY polymorphism. All tubes will be labeled with a coded, anonymous identifier.

**Genotyping will be performed by allele-specific primer extension of single-plex amplified products, with detection by matrix-assisted laser desorption ionization-time of flight mass spectroscopy on a Sequenom platform (**[**82**](#_ENREF_82)**).** Hardy-Weinberg equilibrium will be tested within each self-described ethnic group, and overall call and consensus rates will be determined. In our hands, this genotyping platform routinely produces call rates >98% and consensus rates >99%. Genotyping activities will take place in collaboration with the Genomics Platform at the Broad Institute, of which the PI is an Associate Member.  Similarly, metabolomic profiling will take place in collaboration with the Metabolite Profiling Platform at the Broad Institute.  Only samples and basic phenotypic data will be shared with investigators at the Broad Institute; all PHI will remain at the MGH site under password-protected electronic files and/or in a locked office.

**V.8.c. GLP-1 measurements**

**GLP-1 will be measured through radioimmunoassays validated in the MGH-based Boston Area Diabetes Endocrinology Research Center (P30) core laboratories.** Due to the relatively short half-life of active GLP-1 (amino acids 7-36) that is cleaved by DPPIV to generate inactive GLP-1 (amino acids 9-36), we plan to measure both total and active GLP-1 levels in our subjects. We will use a specific antibody to the C-terminus of GLP-1 to measure total GLP-1 levels, and a second specific antibody targeting the N-terminus of GLP-1 to measure the active GLP-1 (amino acids 7-36) levels. Laboratory technician Varinderpal Kaur, who has extensive longitudinal experience in conducting GLP-1 assays, will perform these measurements. For GLP-1 measurements, blood samples will be collected in prechilled tubes containing EDTA, kallikrein-trypsin inhibitor (Trasylol), and diprotin A. GLP-1 radioimmunoassays will be conducted according to established laboratory protocols ([83](#_ENREF_83)).

1. **Data Analysis**

***VI.1. Study endpoints***

**The study will have the following primary endpoints, according to each Specific Aim:**

Specific Aim No. 1 – Sulfonylurea challenge: Trough glucose and peak insulin levels will be compared by genotype. Secondary endpoints will be differences in glucose and insulin areas under the curve (AUC, calculated by the trapezoidal method), as well as glucose and insulin at 2 hours. Leftover serum samples will be frozen for glucagon level measurements.

Specific Aim No. 2 – Acute metformin effect on insulin sensitivity: The insulin sensitivity index (reciprocal of insulin resistance by homeostasis model assessment ([84](#_ENREF_84))) will be calculated as 22.5/[fasting insulin x (fasting glucose/18.01)], and compared by genotype. A secondary endpoint will be differences in the insulinogenic index by genotype after metformin treatment, calculated as [(insulin at 30 min) – (insulin at 0 min)]/[(glucose at 30 min) – (glucose at 0 min)].

Specific Aim No. 3 – insulinogenic index and GLP-1 levels during OGTT: The insulinogenic index, calculated as above, will be compared by genotype, and the differences between genotypic groups will be contrasted to those observed during the sulfonylurea challenge (Specific Aim No. 1). A subset of subjects (selected to have equal numbers in each genotypic group) will have their GLP-1 levels measured, and peak GLP-1 as well as AUC will be compared by genotype. Leftover serum samples will be frozen for metabolomic profiling and glucagon level measurements.

***VI.2. Statistical analysis***

**Non-normal variables will be log transformed.** They will be adjusted for age, gender and BMI, and residuals will be compared between each group of homozygous individuals at each locus by Student t tests. Since these polymorphisms may influence glycemic traits by affecting BMI, both BMI-adjusted and unadjusted analyses will be reported. Secondary analyses will include exploration of genetic models (dominant, recessive or additive), by including heterozygous individuals and performing ANOVA across the three genotypic groups, with subsequent Bonferroni adjustment for multiple comparisons. The role of self-reported ethnicity will be evaluated with an interaction term; analyses will also be repeated within each self-reported ethnic group, and in the aggregate of groups where minor allele frequencies are comparable. As these experiments involve validation of previous hypotheses, nominal *P* values below 0.05 will be considered statistically significant.

***VI.3. Power calculations***

**Our power calculations suggest that we will need to enroll ~750 subjects.** Based on our own multi-ethnic data, we have estimated a minor allele frequency of 0.32 for the risk T allele at rs7903146 *TCF7L2* and 0.35 for the risk K allele at *KCNJ11* E23K. Assuming Hardy-Weinberg equilibrium, the proportion of subjects carrying the risk homozygous genotype at either locus will be ~10% and ~12% respectively. With the difference in insulinogenic index between CC and TT homozygotes at rs7903146 *TCF7L2* documented by Saxena *et al.,* (see Section C.3 above), 750 subjects should provide >80% power to detect the same difference at a one-sided alpha of 0.05. Similarly, assuming that the difference in insulin sensitivity after one year of metformin treatment by E23K genotype is also detectable in the acute setting, 750 subjects provide >90% power at the same one-sided alpha of 0.05 (73% for a two-sided test). With regard to the sulfonylurea challenge, this sample size has >99% power to detect a 10% difference in trough glucose between genotypic groups at a one-sided alpha of 0.05, and 94% power to detect a 20% difference in peak insulin at a one-sided alpha of 0.05.

While these power calculations apply to a study population of a single ethnic group, expanding our study to include a multi-ethnic population would require enrollment of ~1,000 subjects.

1. **Risks and DIscomforts**

***VII.1. Risks to the subjects and implementation of safeguards***

**We will enroll 1,000 male or non-pregnant female adults with early type 2 diabetes (on diet treatment only).** No vulnerable populations will be enrolled. Although glipizide is labeled as a pregnancy Class C drug and metformin as a Class B drug, no pregnant or nursing women will be enrolled; in part, this is due to the known alterations in glycemic physiology during pregnancy. Women at risk of being pregnant (i.e. sexually active without adequate birth control) will receive a urine pregnancy test before enrollment.

**In order to avoid an exaggerated response to either of the two study medications, we will exclude subjects with impaired renal or hepatic function.** This will be initially determined by both history and examination of the medical record, which will be available to us due to our exclusive use of the Partners-specific source database. Renal dysfunction will be defined as a decrease in estimated GFR < 60 ml/min/1.73 m^2^, based on the most recent serum creatinine measurement available in the electronic medical record (within the past year), and calculated by the Modification of Diet in Renal Disease equation ([81](#_ENREF_81)). Subjects with advanced diabetes (i.e. on antidiabetic medications), type 1 diabetes (i.e. on insulin therapy) or suspected Maturity Onset Diabetes of the Young (based on age at diagnosis and familial transmission) will also be excluded. Finally, subjects with a history of porphyria or severe sulfonamide allergy will be excluded to avoid potential hypersensitivity reactions to glipizide.

**In order to prevent the rare potential complication of *lactic acidosis* in subjects with impaired renal function who receive metformin, we will recalculate GFR based on a current serum creatinine level.** A serum creatinine level will be measured on Day 1, and current GFR will be estimated by the Modification of Diet in Renal Disease equation ([81](#_ENREF_81)). Because subjects with GFR < 60 ml/min/1.73 m^2^ (based on a recent historical creatinine measurement) will have been excluded from enrollment, we expect that only a few subjects will meet this definition after enrollment. These subjects will be able to complete the sulfonylurea challenge (as glipizide does not require dose adjustment based on renal function), but will not receive metformin for the second phase of this study (due to begin on Day 6): instead, they will undergo a simple OGTT on Day 8 in the absence of any metformin treatment. In addition, if they are being treated with diuretic therapy we will make sure their doses are stable throughout the study. Likewise, we will be performing an ALT/AST measurement, and if the result is 3x higher than the upper limit of normal the subject will not take the metformin, and instead report for a simple OGTT. As a further precaution, any subject with a planned radiologic or angiographic study requiring IV contrast within one week of completion of this study will also be excluded from metformin treatment, because iodinated contrast may precipitate the acute alteration of renal function, and this might lead to metformin accumulation and resulting lactic acidosis. Finally, subjects will also be instructed to limit their alcohol intake to one drink or less on the night preceding their second visit while on active metformin therapy.

**After these exclusion criteria are implemented, the risks to the subjects will be minor.** These include 1) hypoglycemia, 2) blood drawing, 3) gastrointestinal side effects due to metformin, 4) stress from a potential diagnosis of pre-diabetes or diabetes and 5) loss of confidentiality. We will address these sequentially.

- - 1. **During the sulfonylurea challenge, subjects are expected to become hypoglycemic.** The degree of hypoglycemia may be blunted in our population at risk of diabetes, for whom some amount of insulin resistance is likely to be present. In addition to the exclusion of subjects with impaired renal or hepatic function (in whom duration of action may be more pronounced), we will take the following precautions to avoid complications of severe hypoglycemia: 1) exclusion of subjects with a fasting fingerstick < 80 mg/dl, 2) selection of a low dose of glipizide, 3) close monitoring of subjects for the appearance of hypoglycemic signs and symptoms (diaphoresis, nervousness, jitteriness, shakiness, confusion, blurred vision, lightheadedness, slurred speech), 4) rapid fingerstick measurement if any of the above are noted, 5) half-hourly fingerstick glucose measurements even in the absence of symptoms, 6) increase in the frequency of fingerstick measurements (even when asymptomatic) if these drop below 50 mg/dl, and 7) termination of the challenge with juice and a glucotab if the fingerstick is <50 mg/dl (with symptoms) or <45 mg/dl (without symptoms), with indications that the subject break his/her fast immediately. We will also have IV dextrose on standby, and will provide all subjects with a carbohydrate-rich meal at the conclusion of the challenge. Metformin only causes hypoglycemia when used in combination with other hypoglycemic agents; in order to avoid this eventuality we have established a safe washout period. Even with the conservative estimation of prolonged glipizide half-life in the elderly (~10h), a total of 12 half-lives will have transpired between Day 1 and Day 6, essentially eliminating any chance of both drugs interacting in any subject.
    2. **Blood drawing will be minimal and spread out over one week.** On Day 1, blood work will involve baseline measurements (35.5 cc), DNA 20 cc, two time points with 22cc of blood and four time points with 17cc of blood each, for a total of 167.5 cc of blood (~11.3 tablespoons). On Day 8, blood work will involve a baseline measurement of 27 cc, two time points with 22cc of blood, one time point~~s~~ with 17cc and three time points with 10cc. This totals 118cc (~8.0 tablespoons) of blood taken during Day 8 and 285.5cc (~.3 tablespoons) for both days. A 20G intravenous catheter will be placed in the antecubital vein each day, in order to minimize the discomfort of repeated venipuncture. Occasionally a bruise may be produced; rarely, infiltration of the catheter with a resulting local skin reaction may also occur. Some subjects may experience syncope due to catheter insertion.
    3. **Metformin may cause loose stools or overt diarrhea.** In order to minimize this side effect, which occurs in ~15% of subjects, we have elected to use the lowest single dose used clinically (500 mg) and limit it to four doses only. If diarrhea becomes intolerable the subject may elect to discontinue metformin, and will undergo a simple OGTT on Day 8 instead.
    4. **Subjects with previously undiagnosed pre-diabetes or diabetes may have results consistent with either of these diagnoses.** As the study is targeted toward individuals at risk of developing diabetes, this eventuality will be prominently discussed during the informed consent process. A fasting glucose between 100 and 125 mg/dl in either visit (indicating impaired fasting glucose) or a 2h OGTT between 140 and 200 mg/dl (indicating impaired glucose tolerance) would be suggestive of pre-diabetes. A fasting glucose ≥ 126 mg/dl in either visit or 2h OGTT glucoses ≥ 200 mg/dl would be suggestive of diabetes; because the diagnosis of pre-diabetes or diabetes requires repeat confirmation and the OGTT will be performed under the influence of metformin, this study will not make a definitive diagnosis. Subjects who meet these criteria or who have other laboratory values deemed by the study physicians to be clinically actionable will be informed and asked to follow up with their primary care physician, who may also be notified if the subject agrees. Some of these values are not required as safety measures in our study, but are provided to us by the laboratory because the measurement of study-required creatinine or liver transaminases occurs in a bundle with other electrolytes and liver function tests.  These other incidental laboratory values will include results outside of the following ranges, determined by our study physicians to merit clinical action: sodium 130-150 mmol/L, potassium 3.2-5.2 mmol/L, 8.5-11 mg/dL, or alkaline phosphatase above 300 IU/L. Owing to the above considerations, results will not be entered into the medical record.
    5. **Subject confidentiality will be protected.** In order to protect the privacy of the subjects, we will provide them with a coded, anonymous numerical identifier at enrollment. Anthropomorphic, biochemical, , and genetic data will be linked to this anonymous identifier only, and will not be part of the medical record. The key will be stored in the PI’s office in a locked cabinet and in his password-protected hard drive. Only study personnel who have undergone appropriate human research training and signed standard confidentiality agreements will have access to these data. All subject-related documents will be stored in locked file cabinets within locked offices.

1. **Potential Benefits**

**This study will have no personal direct benefit to subjects, other than provide initial diagnostic tests which may indicate pre-diabetes or the presence of diabetes.** On a societal level, however, this proposal should help clarify the pathophysiologic mechanisms by which these key genetic variants increase risk of type 2 diabetes, and assess their impact on commonly used antidiabetic treatments. In addition, this pilot study will lay the groundwork for a long-term, outcomes-based pharmacogenetic clinical trial.

**A number of genetic variants have already been reproducibly associated with type 2 diabetes; the list is only expected to grow.** It will be crucial to harness this new genetic knowledge so that it can refine our understanding of the pathophysiology of diverse forms of diabetes, enhance our prognostic ability and direct our choice of appropriate therapies. The discovery that some of these polymorphisms have measureable effects on glycemic parameters opens the door to targeted pharmacogenetic studies. The information obtained from pilot experiments such as the ones outlined in this proposal should provide the foundation necessary to design and implement genome-based clinical trials, with the hope that these novel genetic insights will translate into improved medical care and preventive measures for public health.

1. **Monitoring and Quality Assurance**

***IX.1. Data monitoring***

**A Data Safety Monitoring Plan will be implemented.** The PI will review the safety and progress of this study on a monthly basis. At the request of the NIH, the DSMP has been modified to include a Designated Safety Officer not involved in the conduct of the study. Dr. Enrico Cagliero of the Diabetes Center at MGH has graciously agreed to perform this function, and will meet with the PI and the Research Coordinator on a quarterly basis to monitor subject safety. In addition, the PI will include results of the review in the annual progress reports submitted to the clinical research center, IRB, and NIDDK. The annual report will include a list of adverse events. It will address: (1) whether adverse event rates are consistent with pre-study assumptions; (2) reason for dropouts from the study; (3) whether all participants met entry criteria; (4) whether continuation of the study is justified on the basis that additional data are needed to accomplish the stated aims of the study; and (5) conditions whereby the study might be terminated prematurely. Finally, the research review committee at the clinical research center will review each protocol annually for safety.

***IX.2. Adverse events***

**IX.2.a. Adverse event grading**

1. Attribution scale. An adverse event is defined as both an expected side effect that is of a serious nature, or an unexpected side effect/event regardless of severity. All events will be graded as to their attribution (unlikely, possibly, probably, or definitely related to protocol) and their severity (mild, moderate or severe). **Severe or serious** adverse events are events that result in death, a life threatening experience, hospitalization, persistent or significant disability, a congenital birth defect or a medical intervention designed to prevent any of the above; **moderate** adverse events cause discomfort enough to interfere with usual activities, are persistent and/or require medical evaluation and treatment; **mild** adverse events involve the awareness of signs and symptoms that are easily tolerated, cause no loss of time from normal activities, do not require medical evaluation and/or treatment, and are transient. Any event that is reported to either the PI or his designated research associates by the subject or medical staff caring for the subject and which meets the criteria will be documented as such.
2. Expected risks. As detailed in the protocol and consent form, the expected risks include:

- Hypoglycemia during sulfonylurea challenge
- Blood drawing and intravenous catheter insertion
- Gastrointestinal side effects due to metformin
- Stress from a potential diagnosis of diabetes, and
- Loss of confidentiality.

**IX.2.b. Plan for reporting both anticipated and unanticipated adverse events**

**Each subject is evaluated for any adverse events.** Any event that is reported to either the PI or his designated research associates by the subject or medical staff caring for the subject and which meets the criteria will be documented as such. Any event that is reported will then generate an adverse event report, which will be submitted to the IRB and the clinical research center. The report will include a description of the event, when and how it was reported, as well as any official chart records or documentation to corroborate the event or the reporting of the event.

**Any severe and/or unanticipated adverse event will be immediately reported to the IRB and the clinical research center.** All other adverse events will be reported in a timely fashion to the IRB and the clinical research center, preferably within 2 weeks of the date of the event. All adverse events will be summarized annually and submitted to the IRB and the clinical research center. Any action resulting in a temporary or permanent suspension of this study (e.g. FDA actions, IRB actions, or actions by a commercial sponsor or by the investigators or co-investigators) will be reported to the appropriate NIDDK program official.

**Bibliography and References Cited**

1. Barroso I: Genetics of type 2 diabetes. Diabetic Medicine 2005;22:517-535

2. Florez JC, Hirschhorn JN, Altshuler D: The inherited basis of diabetes mellitus: implications for the genetic analysis of complex traits. Annual review of genomics and human genetics 2003;4:257-291

3. Fajans SS, Bell GI, Polonsky KS: Molecular mechanisms and clinical pathophysiology of maturity-onset diabetes of the young. N Engl J Med 2001;345:971-980

4. Deeb SS, Fajas L, Nemoto M, Pihlajamaki J, Mykkanen L, Kuusisto J, Laakso M, Fujimoto W, Auwerx J: A Pro12Ala substitution in PPARγ2 associated with decreased receptor activity, lower body mass index and improved insulin sensitivity. Nat Genet 1998;20:284-287

5. Altshuler D, Hirschhorn JN, Klannemark M, Lindgren CM, Vohl MC, Nemesh J, Lane CR, Schaffner SF, Bolk S, Brewer C, Tuomi T, Gaudet D, Hudson TJ, Daly M, Groop L, Lander ES: The common PPARγ Pro12Ala polymorphism is associated with decreased risk of type 2 diabetes. Nat Genet 2000;26:76-80

6. Hara K, Okada T, Tobe K, Yasuda K, Mori Y, Kadowaki H, Hagura R, Akanuma Y, Kimura S, Ito C, Kadowaki T: The Pro12Ala polymorphism in PPAR gamma2 may confer resistance to type 2 diabetes. Biochem Biophys Res Commun 2000;271:212-216

7. Ardlie KG, Lunetta KL, Seielstad M: Testing for population subdivision and association in four case-control studies. American journal of human genetics 2002;71:304-311

8. Mori H, Ikegami H, Kawaguchi Y, Seino S, Yokoi N, Takeda J, Inoue I, Seino Y, Yasuda K, Hanafusa T, Yamagata K, Awata T, Kadowaki T, Hara K, Yamada N, Gotoda T, Iwasaki N, Iwamoto Y, Sanke T, Nanjo K, Oka Y, Matsutani A, Maeda E, Kasuga M: The Pro12 ® Ala substitution in PPAR-γ is associated with resistance to development of diabetes in the general population: possible involvement in impairment of insulin secretion in individuals with type 2 diabetes. Diabetes 2001;50:891-894.

9. Douglas JA, Erdos MR, Watanabe RM, Braun A, Johnston CL, Oeth P, Mohlke KL, Valle TT, Ehnholm C, Buchanan TA, Bergman RN, Collins FS, Boehnke M, Tuomilehto J: The peroxisome proliferator-activated receptor-γ2 Pro12Ala variant: association with type 2 diabetes and trait differences. Diabetes 2001;50:886-890

10. Ghoussaini M, Meyre D, Lobbens S, Charpentier G, Clement K, Charles M-A, Tauber M, Weill J, Froguel P: Implication of the Pro12Ala polymorphism of the PPAR-gamma 2 gene in type 2 diabetes and obesity in the French population. BMC Medical Genetics 2005;6:11

11. Gloyn AL, Pearson ER, Antcliff JF, Proks P, Bruining GJ, Slingerland AS, Howard N, Srinivasan S, Silva JMCL, Molnes J, Edghill EL, Frayling TM, Temple IK, Mackay D, Shield JPH, Sumnik Z, van Rhijn A, Wales JKH, Clark P, Gorman S, Aisenberg J, Ellard S, Njolstad PR, Ashcroft FM, Hattersley AT: Activating mutations in the gene encoding the ATP-sensitive potassium-channel subunit Kir6.2 and permanent neonatal diabetes. N Engl J Med 2004;350:1838-1849

12. Hani EH, Boutin P, Durand E, Inoue H, Permutt MA, Velho G, Froguel P: Missense mutations in the pancreatic islet beta cell inwardly rectifying K+ channel gene (KIR6.2/BIR): a meta-analysis suggests a role in the polygenic basis of Type II diabetes mellitus in Caucasians. Diabetologia 1998;41:1511-1515

13. Gloyn AL, Hashim Y, Ashcroft SJ, Ashfield R, Wiltshire S, Turner RC: Association studies of variants in promoter and coding regions of beta-cell ATP-sensitive K-channel genes SUR1 and Kir6.2 with Type 2 diabetes mellitus (UKPDS 53). Diabet Med 2001;18:206-212

14. Gloyn AL, Weedon MN, Owen KR, Turner MJ, Knight BA, Hitman G, Walker M, Levy JC, Sampson M, Halford S, McCarthy MI, Hattersley AT, Frayling TM: Large-scale association studies of variants in genes encoding the pancreatic β-cell KATP channel subunits Kir6.2 (*KCNJ11*) and SUR1 (*ABCC8*) confirm that the *KCNJ11* E23K variant is associated with type 2 diabetes. Diabetes 2003;52:568-572

15. Nielsen E-MD, Hansen L, Carstensen B, Echwald SM, Drivsholm T, Glumer C, Thorsteinsson B, Borch-Johnsen K, Hansen T, Pedersen O: The E23K variant of Kir6.2 associates with impaired post-OGTT serum insulin response and increased risk of type 2 diabetes. Diabetes 2003;52:573-577

16. Love-Gregory L, Wasson J, Lin J, Skolnick G, Suarez B, Permutt MA: E23K single nucleotide polymorphism in the islet ATP-sensitive potassium channel gene (Kir6.2) contributes as much to the risk of type II diabetes in Caucasians as the PPARγ Pro12Ala variant. Diabetologia 2003;46:136-137

17. Barroso I, Luan J, Middelberg RPS, Harding A-H, Franks PW, Jakes RW, Clayton D, Schafer AJ, O'Rahilly S, Wareham NJ: Candidate gene association study in type 2 diabetes indicates a role for genes involved in ß-cell function as well as insulin action. PLoS Biology 2003;1:e20

18. Florez JC, Burtt N, de Bakker PIW, Almgren P, Tuomi T, Holmkvist J, Gaudet D, Hudson TJ, Schaffner SF, Daly MJ, Hirschhorn JN, Groop L, Altshuler D: Haplotype structure and genotype-phenotype correlations of the sulfonylurea receptor and the islet ATP-sensitive potassium channel gene region. Diabetes 2004;53:1360-1368

19. Lyssenko V, Almgren P, Anevski D, Orho-Melander M, Sjögren M, Saloranta C, Tuomi T, Groop L, the Botnia Study G: Genetic prediction of future type 2 diabetes. PLoS Medicine 2005;2:e345

20. Schwanstecher C, Meyer U, Schwanstecher M: KIR6.2 polymorphism predisposes to type 2 diabetes by inducing overactivity of pancreatic β-cell ATP-sensitive K+ channels. Diabetes 2002;51:875-879

21. Riedel MJ, Light PE: Saturated and cis/trans unsaturated acyl CoA esters differentially regulate wild-type and polymorphic β-cell ATP-sensitive K+ channels. Diabetes 2005;54:2070-2079

22. Grant SFA, Thorleifsson G, Reynisdottir I, Benediktsson R, Manolescu A, Sainz J, Helgason A, Stefansson H, Emilsson V, Helgadottir A, Styrkarsdottir U, Magnusson KP, Walters GB, Palsdottir E, Jonsdottir T, Gudmundsdottir T, Gylfason A, Saemundsdottir J, Wilensky RL, Reilly MP, Rader DJ, Bagger Y, Christiansen C, Gudnason V, Sigurdsson G, Thorsteinsdottir U, Gulcher JR, Kong A, Stefansson K: Variant of transcription factor 7-like 2 (*TCF7L2*) gene confers risk of type 2 diabetes. Nat Genet 2006;38:320-323

23. Ioannidis JP, Ntzani EE, Trikalinos TA, Contopoulos-Ioannidis DG: Replication validity of genetic association studies. Nat Genet 2001;29:306-309

24. Hirschhorn JN, Lohmueller K, Byrne E, Hirschhorn K: A comprehensive review of genetic association studies. Genetics in Medicine 2002;4:45-61

25. Lohmueller K, Pearce CL, Pike M, Lander ES, Hirschhorn JN: Meta-analysis of genetic association studies supports a contribution of common variants to susceptibility to common disease. Nat Genet 2003;33:177-182

26. Florez JC: Phenotypic consequences of the peroxisome proliferator-activated receptor-γ Pro12Ala polymorphism: the weight of the evidence in genetic association studies. J Clin Endocrinol Metab 2004;89:4234-4237

27. van Dam RM, Hoebee B, Seidell JC, Schaap MM, de Bruin TWA, Feskens EJM: Common variants in the ATP-sensitive K+ channel genes KCNJ11 (Kir6.2) and ABCC8 (SUR1) in relation to glucose intolerance: population-based studies and meta-analyses. Diabetic Medicine 2005;22:590-598

28. McCarthy MI, Abecasis GR, Cardon LR, Goldstein DB, Little J, Ioannidis JP, Hirschhorn JN: Genome-wide association studies for complex traits: consensus, uncertainty and challenges. Nature reviews 2008;9:356-369

29. Sladek R, Rocheleau G, Rung J, Dina C, Shen L, Serre D, Boutin P, Vincent D, Belisle A, Hadjadj S, Balkau B, Heude B, Charpentier G, Hudson TJ, Montpetit A, Pshezhetsky AV, Prentki M, Posner BI, Balding DJ, Meyre D, Polychronakos C, Froguel P: A genome-wide association study identifies novel risk loci for type 2 diabetes. Nature 2007;445:828-830

30. Diabetes Genetics Initiative of Broad Institute of Harvard and MIT, Lund University and Novartis Institutes for BioMedical R, Saxena R, Voight BF, Lyssenko V, Burtt NP, de Bakker PIW, Chen H, Roix JJ, Kathiresan S, Hirschhorn JN, Daly MJ, Hughes TE, Groop L, Altshuler D, Almgren P, Florez JC, Meyer J, Ardlie K, Bengtsson K, Isomaa B, Lettre G, Lindblad U, Lyon HN, Melander O, Newton-Cheh C, Nilsson P, Orho-Melander M, Rastam L, Speliotes EK, Taskinen M-R, Tuomi T, Guiducci C, Berglund A, Carlson J, Gianniny L, Hackett R, Hall L, Holmkvist J, Laurila E, Sjogren M, Sterner M, Surti A, Svensson M, Svensson M, Tewhey R, Blumenstiel B, Parkin M, DeFelice M, Barry R, Brodeur W, Camarata J, Chia N, Fava M, Gibbons J, Handsaker B, Healy C, Nguyen K, Gates C, Sougnez C, Gage D, Nizzari M, Gabriel SB, Chirn G-W, Ma Q, Parikh H, Richardson D, Ricke D, Purcell S: Genome-wide association analysis identifies loci for type 2 diabetes and triglyceride levels. Science 2007;316:1331-1336

31. Zeggini E, Weedon MN, Lindgren CM, Frayling TM, Elliott KS, Lango H, Timpson NJ, Perry JRB, Rayner NW, Freathy RM, Barrett JC, Shields B, Morris AP, Ellard S, Groves CJ, Harries LW, Marchini JL, Owen KR, Knight B, Cardon LR, Walker M, Hitman GA, Morris AD, Doney ASF, The Wellcome Trust Case Control C, McCarthy MI, Hattersley AT: Replication of genome-wide association signals in U.K. samples reveals risk loci for type 2 diabetes. Science 2007;316:1336-1341

32. Scott LJ, Mohlke KL, Bonnycastle LL, Willer CJ, Li Y, Duren WL, Erdos MR, Stringham HM, Chines PS, Jackson AU, Prokunina-Olsson L, Ding C-J, Swift AJ, Narisu N, Hu T, Pruim R, Xiao R, Li X-Y, Conneely KN, Riebow NL, Sprau AG, Tong M, White PP, Hetrick KN, Barnhart MW, Bark CW, Goldstein JL, Watkins L, Xiang F, Saramies J, Buchanan TA, Watanabe RM, Valle TT, Kinnunen L, Abecasis GR, Pugh EW, Doheny KF, Bergman RN, Tuomilehto J, Collins FS, Boehnke M: A genome-wide association study of type 2 diabetes in Finns detects multiple susceptibility variants. Science 2007;316:1341-1345

33. Steinthorsdottir V, Thorleifsson G, Reynisdottir I, Benediktsson R, Jonsdottir T, Walters GB, Styrkarsdottir U, Gretarsdottir S, Emilsson V, Ghosh S, Baker A, Snorradottir S, Bjarnason H, Ng MC, Hansen T, Bagger Y, Wilensky RL, Reilly MP, Adeyemo A, Chen Y, Zhou J, Gudnason V, Chen G, Huang H, Lashley K, Doumatey A, So WY, Ma RC, Andersen G, Borch-Johnsen K, Jorgensen T, van Vliet-Ostaptchouk JV, Hofker MH, Wijmenga C, Christiansen C, Rader DJ, Rotimi C, Gurney M, Chan JC, Pedersen O, Sigurdsson G, Gulcher JR, Thorsteinsdottir U, Kong A, Stefansson K: A variant in *CDKAL1* influences insulin response and risk of type 2 diabetes. Nat Genet 2007;39:770-775

34. Zeggini E, Scott LJ, Saxena R, Voight BF, Marchini JL, Hu T, de Bakker PI, Abecasis GR, Almgren P, Andersen G, Ardlie K, Bostrom KB, Bergman RN, Bonnycastle LL, Borch-Johnsen K, Burtt NP, Chen H, Chines PS, Daly MJ, Deodhar P, Ding CJ, Doney AS, Duren WL, Elliott KS, Erdos MR, Frayling TM, Freathy RM, Gianniny L, Grallert H, Grarup N, Groves CJ, Guiducci C, Hansen T, Herder C, Hitman GA, Hughes TE, Isomaa B, Jackson AU, Jorgensen T, Kong A, Kubalanza K, Kuruvilla FG, Kuusisto J, Langenberg C, Lango H, Lauritzen T, Li Y, Lindgren CM, Lyssenko V, Marvelle AF, Meisinger C, Midthjell K, Mohlke KL, Morken MA, Morris AD, Narisu N, Nilsson P, Owen KR, Palmer CN, Payne F, Perry JR, Pettersen E, Platou C, Prokopenko I, Qi L, Qin L, Rayner NW, Rees M, Roix JJ, Sandbaek A, Shields B, Sjogren M, Steinthorsdottir V, Stringham HM, Swift AJ, Thorleifsson G, Thorsteinsdottir U, Timpson NJ, Tuomi T, Tuomilehto J, Walker M, Watanabe RM, Weedon MN, Willer CJ, Illig T, Hveem K, Hu FB, Laakso M, Stefansson K, Pedersen O, Wareham NJ, Barroso I, Hattersley AT, Collins FS, Groop L, McCarthy MI, Boehnke M, Altshuler D: Meta-analysis of genome-wide association data and large-scale replication identifies additional susceptibility loci for type 2 diabetes. Nat Genet 2008;40:638-645

35. Voight BF, Scott LJ, Steinthorsdottir V, Morris AP, Dina C, Welch RP, Zeggini E, Huth C, Aulchenko YS, Thorleifsson G, McCulloch LJ, Ferreira T, Grallert H, Amin N, Wu G, Willer CJ, Raychaudhuri S, McCarroll SA, Langenberg C, Hofmann OM, Dupuis J, Qi L, Segre AV, van Hoek M, Navarro P, Ardlie K, Balkau B, Benediktsson R, Bennett AJ, Blagieva R, Boerwinkle E, Bonnycastle LL, Bengtsson Bostrom K, Bravenboer B, Bumpstead S, Burtt NP, Charpentier G, Chines PS, Cornelis M, Couper DJ, Crawford G, Doney AS, Elliott KS, Elliott AL, Erdos MR, Fox CS, Franklin CS, Ganser M, Gieger C, Grarup N, Green T, Griffin S, Groves CJ, Guiducci C, Hadjadj S, Hassanali N, Herder C, Isomaa B, Jackson AU, Johnson PR, Jorgensen T, Kao WH, Klopp N, Kong A, Kraft P, Kuusisto J, Lauritzen T, Li M, Lieverse A, Lindgren CM, Lyssenko V, Marre M, Meitinger T, Midthjell K, Morken MA, Narisu N, Nilsson P, Owen KR, Payne F, Perry JR, Petersen AK, Platou C, Proenca C, Prokopenko I, Rathmann W, Rayner NW, Robertson NR, Rocheleau G, Roden M, Sampson MJ, Saxena R, Shields BM, Shrader P, Sigurdsson G, Sparso T, Strassburger K, Stringham HM, Sun Q, Swift AJ, Thorand B, Tichet J, Tuomi T, van Dam RM, van Haeften TW, van Herpt T, van Vliet-Ostaptchouk JV, Bragi Walters G, Weedon MN, Wijmenga C, Witteman J, Bergman RN, Cauchi S, Collins FS, Gloyn AL, Gyllensten U, Hansen T, Hide WA, Hitman GA, Hofman A, Hunter DJ, Hveem K, Laakso M, Mohlke KL, Morris AD, Palmer CN, Pramstaller PP, Rudan I, Sijbrands E, Stein LD, Tuomilehto J, Uitterlinden A, Walker M, Wareham NJ, Watanabe RM, Abecasis GR, Boehm BO, Campbell H, Daly MJ, Hattersley AT, Hu FB, Meigs JB, Pankow JS, Pedersen O, Wichmann HE, Barroso I, Florez JC, Frayling TM, Groop L, Sladek R, Thorsteinsdottir U, Wilson JF, Illig T, Froguel P, van Duijn CM, Stefansson K, Altshuler D, Boehnke M, McCarthy MI: Twelve type 2 diabetes susceptibility loci identified through large-scale association analysis. Nat Genet 2010;42:579-589

36. McCarthy MI: Genomics, type 2 diabetes, and obesity. N Engl J Med 2010;363:2339-2350

37. Prokopenko I, Langenberg C, Florez JC, Saxena R, Soranzo N, Thorleifsson G, Loos RJ, Manning AK, Jackson AU, Aulchenko Y, Potter SC, Erdos MR, Sanna S, Hottenga JJ, Wheeler E, Kaakinen M, Lyssenko V, Chen WM, Ahmadi K, Beckmann JS, Bergman RN, Bochud M, Bonnycastle LL, Buchanan TA, Cao A, Cervino A, Coin L, Collins FS, Crisponi L, de Geus EJ, Dehghan A, Deloukas P, Doney AS, Elliott P, Freimer N, Gateva V, Herder C, Hofman A, Hughes TE, Hunt S, Illig T, Inouye M, Isomaa B, Johnson T, Kong A, Krestyaninova M, Kuusisto J, Laakso M, Lim N, Lindblad U, Lindgren CM, McCann OT, Mohlke KL, Morris AD, Naitza S, Orru M, Palmer CN, Pouta A, Randall J, Rathmann W, Saramies J, Scheet P, Scott LJ, Scuteri A, Sharp S, Sijbrands E, Smit JH, Song K, Steinthorsdottir V, Stringham HM, Tuomi T, Tuomilehto J, Uitterlinden AG, Voight BF, Waterworth D, Wichmann HE, Willemsen G, Witteman JC, Yuan X, Zhao JH, Zeggini E, Schlessinger D, Sandhu M, Boomsma DI, Uda M, Spector TD, Penninx BW, Altshuler D, Vollenweider P, Jarvelin MR, Lakatta E, Waeber G, Fox CS, Peltonen L, Groop LC, Mooser V, Cupples LA, Thorsteinsdottir U, Boehnke M, Barroso I, Van Duijn C, Dupuis J, Watanabe RM, Stefansson K, McCarthy MI, Wareham NJ, Meigs JB, Abecasis GR: Variants in *MTNR1B* influence fasting glucose levels. Nat Genet 2009;41:77-81

38. Dupuis J, Langenberg C, Prokopenko I, Saxena R, Soranzo N, Jackson AU, Wheeler E, Glazer NL, Bouatia-Naji N, Gloyn AL, Lindgren CM, Magi R, Morris AP, Randall J, Johnson T, Elliott P, Rybin D, Thorleifsson G, Steinthorsdottir V, Henneman P, Grallert H, Dehghan A, Hottenga JJ, Franklin CS, Navarro P, Song K, Goel A, Perry JR, Egan JM, Lajunen T, Grarup N, Sparso T, Doney A, Voight BF, Stringham HM, Li M, Kanoni S, Shrader P, Cavalcanti-Proenca C, Kumari M, Qi L, Timpson NJ, Gieger C, Zabena C, Rocheleau G, Ingelsson E, An P, O'Connell J, Luan J, Elliott A, McCarroll SA, Payne F, Roccasecca RM, Pattou F, Sethupathy P, Ardlie K, Ariyurek Y, Balkau B, Barter P, Beilby JP, Ben-Shlomo Y, Benediktsson R, Bennett AJ, Bergmann S, Bochud M, Boerwinkle E, Bonnefond A, Bonnycastle LL, Borch-Johnsen K, Bottcher Y, Brunner E, Bumpstead SJ, Charpentier G, Chen YD, Chines P, Clarke R, Coin LJ, Cooper MN, Cornelis M, Crawford G, Crisponi L, Day IN, de Geus EJ, Delplanque J, Dina C, Erdos MR, Fedson AC, Fischer-Rosinsky A, Forouhi NG, Fox CS, Frants R, Franzosi MG, Galan P, Goodarzi MO, Graessler J, Groves CJ, Grundy S, Gwilliam R, Gyllensten U, Hadjadj S, Hallmans G, Hammond N, Han X, Hartikainen AL, Hassanali N, Hayward C, Heath SC, Hercberg S, Herder C, Hicks AA, Hillman DR, Hingorani AD, Hofman A, Hui J, Hung J, Isomaa B, Johnson PR, Jorgensen T, Jula A, Kaakinen M, Kaprio J, Kesaniemi YA, Kivimaki M, Knight B, Koskinen S, Kovacs P, Kyvik KO, Lathrop GM, Lawlor DA, Le Bacquer O, Lecoeur C, Li Y, Lyssenko V, Mahley R, Mangino M, Manning AK, Martinez-Larrad MT, McAteer JB, McCulloch LJ, McPherson R, Meisinger C, Melzer D, Meyre D, Mitchell BD, Morken MA, Mukherjee S, Naitza S, Narisu N, Neville MJ, Oostra BA, Orru M, Pakyz R, Palmer CN, Paolisso G, Pattaro C, Pearson D, Peden JF, Pedersen NL, Perola M, Pfeiffer AF, Pichler I, Polasek O, Posthuma D, Potter SC, Pouta A, Province MA, Psaty BM, Rathmann W, Rayner NW, Rice K, Ripatti S, Rivadeneira F, Roden M, Rolandsson O, Sandbaek A, Sandhu M, Sanna S, Sayer AA, Scheet P, Scott LJ, Seedorf U, Sharp SJ, Shields B, Sigurethsson G, Sijbrands EJ, Silveira A, Simpson L, Singleton A, Smith NL, Sovio U, Swift A, Syddall H, Syvanen AC, Tanaka T, Thorand B, Tichet J, Tonjes A, Tuomi T, Uitterlinden AG, van Dijk KW, van Hoek M, Varma D, Visvikis-Siest S, Vitart V, Vogelzangs N, Waeber G, Wagner PJ, Walley A, Walters GB, Ward KL, Watkins H, Weedon MN, Wild SH, Willemsen G, Witteman JC, Yarnell JW, Zeggini E, Zelenika D, Zethelius B, Zhai G, Zhao JH, Zillikens MC, Borecki IB, Loos RJ, Meneton P, Magnusson PK, Nathan DM, Williams GH, Hattersley AT, Silander K, Salomaa V, Smith GD, Bornstein SR, Schwarz P, Spranger J, Karpe F, Shuldiner AR, Cooper C, Dedoussis GV, Serrano-Rios M, Morris AD, Lind L, Palmer LJ, Hu FB, Franks PW, Ebrahim S, Marmot M, Kao WH, Pankow JS, Sampson MJ, Kuusisto J, Laakso M, Hansen T, Pedersen O, Pramstaller PP, Wichmann HE, Illig T, Rudan I, Wright AF, Stumvoll M, Campbell H, Wilson JF, Bergman RN, Buchanan TA, Collins FS, Mohlke KL, Tuomilehto J, Valle TT, Altshuler D, Rotter JI, Siscovick DS, Penninx BW, Boomsma DI, Deloukas P, Spector TD, Frayling TM, Ferrucci L, Kong A, Thorsteinsdottir U, Stefansson K, van Duijn CM, Aulchenko YS, Cao A, Scuteri A, Schlessinger D, Uda M, Ruokonen A, Jarvelin MR, Waterworth DM, Vollenweider P, Peltonen L, Mooser V, Abecasis GR, Wareham NJ, Sladek R, Froguel P, Watanabe RM, Meigs JB, Groop L, Boehnke M, McCarthy MI, Florez JC, Barroso I: New genetic loci implicated in fasting glucose homeostasis and their impact on type 2 diabetes risk. Nat Genet 2010;42:105-116

39. Saxena R, Hivert MF, Langenberg C, Tanaka T, Pankow JS, Vollenweider P, Lyssenko V, Bouatia-Naji N, Dupuis J, Jackson AU, Kao WH, Li M, Glazer NL, Manning AK, Luan J, Stringham HM, Prokopenko I, Johnson T, Grarup N, Boesgaard TW, Lecoeur C, Shrader P, O'Connell J, Ingelsson E, Couper DJ, Rice K, Song K, Andreasen CH, Dina C, Kottgen A, Le Bacquer O, Pattou F, Taneera J, Steinthorsdottir V, Rybin D, Ardlie K, Sampson M, Qi L, van Hoek M, Weedon MN, Aulchenko YS, Voight BF, Grallert H, Balkau B, Bergman RN, Bielinski SJ, Bonnefond A, Bonnycastle LL, Borch-Johnsen K, Bottcher Y, Brunner E, Buchanan TA, Bumpstead SJ, Cavalcanti-Proenca C, Charpentier G, Chen YD, Chines PS, Collins FS, Cornelis M, G JC, Delplanque J, Doney A, Egan JM, Erdos MR, Firmann M, Forouhi NG, Fox CS, Goodarzi MO, Graessler J, Hingorani A, Isomaa B, Jorgensen T, Kivimaki M, Kovacs P, Krohn K, Kumari M, Lauritzen T, Levy-Marchal C, Mayor V, McAteer JB, Meyre D, Mitchell BD, Mohlke KL, Morken MA, Narisu N, Palmer CN, Pakyz R, Pascoe L, Payne F, Pearson D, Rathmann W, Sandbaek A, Sayer AA, Scott LJ, Sharp SJ, Sijbrands E, Singleton A, Siscovick DS, Smith NL, Sparso T, Swift AJ, Syddall H, Thorleifsson G, Tonjes A, Tuomi T, Tuomilehto J, Valle TT, Waeber G, Walley A, Waterworth DM, Zeggini E, Zhao JH, Illig T, Wichmann HE, Wilson JF, van Duijn C, Hu FB, Morris AD, Frayling TM, Hattersley AT, Thorsteinsdottir U, Stefansson K, Nilsson P, Syvanen AC, Shuldiner AR, Walker M, Bornstein SR, Schwarz P, Williams GH, Nathan DM, Kuusisto J, Laakso M, Cooper C, Marmot M, Ferrucci L, Mooser V, Stumvoll M, Loos RJ, Altshuler D, Psaty BM, Rotter JI, Boerwinkle E, Hansen T, Pedersen O, Florez JC, McCarthy MI, Boehnke M, Barroso I, Sladek R, Froguel P, Meigs JB, Groop L, Wareham NJ, Watanabe RM: Genetic variation in *GIPR* influences the glucose and insulin responses to an oral glucose challenge. Nat Genet 2010;42:142-148

40. Soranzo N, Sanna S, Wheeler E, Gieger C, Radke D, Dupuis J, Bouatia-Naji N, Langenberg C, Prokopenko I, Stolerman E, Sandhu MS, Heeney MM, Devaney JM, Reilly MP, Ricketts SL, Stewart AF, Voight BF, Willenborg C, Wright B, Altshuler D, Arking D, Balkau B, Barnes D, Boerwinkle E, Bohm B, Bonnefond A, Bonnycastle LL, Boomsma DI, Bornstein SR, Bottcher Y, Bumpstead S, Burnett-Miller MS, Campbell H, Cao A, Chambers J, Clark R, Collins FS, Coresh J, de Geus EJ, Dei M, Deloukas P, Doring A, Egan JM, Elosua R, Ferrucci L, Forouhi N, Fox CS, Franklin C, Franzosi MG, Gallina S, Goel A, Graessler J, Grallert H, Greinacher A, Hadley D, Hall A, Hamsten A, Hayward C, Heath S, Herder C, Homuth G, Hottenga JJ, Hunter-Merrill R, Illig T, Jackson AU, Jula A, Kleber M, Knouff CW, Kong A, Kooner J, Kottgen A, Kovacs P, Krohn K, Kuhnel B, Kuusisto J, Laakso M, Lathrop M, Lecoeur C, Li M, Loos RJ, Luan J, Lyssenko V, Magi R, Magnusson PK, Malarstig A, Mangino M, Martinez-Larrad MT, Marz W, McArdle WL, McPherson R, Meisinger C, Meitinger T, Melander O, Mohlke KL, Mooser VE, Morken MA, Narisu N, Nathan DM, Nauck M, O'Donnell C, Oexle K, Olla N, Pankow JS, Payne F, Peden JF, Pedersen NL, Peltonen L, Perola M, Polasek O, Porcu E, Rader DJ, Rathmann W, Ripatti S, Rocheleau G, Roden M, Rudan I, Salomaa V, Saxena R, Schlessinger D, Schunkert H, Schwarz P, Seedorf U, Selvin E, Serrano-Rios M, Shrader P, Silveira A, Siscovick D, Song K, Spector TD, Stefansson K, Steinthorsdottir V, Strachan DP, Strawbridge R, Stumvoll M, Surakka I, Swift AJ, Tanaka T, Teumer A, Thorleifsson G, Thorsteinsdottir U, Tonjes A, Usala G, Vitart V, Volzke H, Wallaschofski H, Waterworth DM, Watkins H, Wichmann HE, Wild SH, Willemsen G, Williams GH, Wilson JF, Winkelmann J, Wright AF, Zabena C, Zhao JH, Epstein SE, Erdmann J, Hakonarson HH, Kathiresan S, Khaw KT, Roberts R, Samani NJ, Fleming MD, Sladek R, Abecasis G, Boehnke M, Froguel P, Groop L, McCarthy MI, Kao WH, Florez JC, Uda M, Wareham NJ, Barroso I, Meigs JB: Common variants at ten genomic loci influence hemoglobin A1C levels via glycemic and non-glycemic pathways. Diabetes 2010;59:3229-3239

41. Strawbridge RJ, Dupuis J, Prokopenko I, Barker A, Ahlqvist E, Rybin D, Petrie JR, Travers ME, Bouatia-Naji N, Dimas AS, Nica A, Wheeler E, Chen H, Voight BF, Taneera J, Kanoni S, Peden JF, Turrini F, Gustafsson S, Zabena C, Almgren P, Barker DJ, Barnes D, Dennison EM, Eriksson JG, Eriksson P, Eury E, Folkersen L, Fox CS, Frayling TM, Goel A, Gu HF, Horikoshi M, Isomaa B, Jackson AU, Jameson KA, Kajantie E, Kerr-Conte J, Kuulasmaa T, Kuusisto J, Loos RJ, Luan J, Makrilakis K, Manning AK, Martinez-Larrad MT, Narisu N, Nastase Mannila M, Ohrvik J, Osmond C, Pascoe L, Payne F, Sayer AA, Sennblad B, Silveira A, Stancakova A, Stirrups K, Swift AJ, Syvanen AC, Tuomi T, van 't Hooft FM, Walker M, Weedon MN, Xie W, Zethelius B, Ongen H, Malarstig A, Hopewell JC, Saleheen D, Chambers J, Parish S, Danesh J, Kooner J, Ostenson CG, Lind L, Cooper CC, Serrano-Rios M, Ferrannini E, Forsen TJ, Clarke R, Franzosi MG, Seedorf U, Watkins H, Froguel P, Johnson P, Deloukas P, Collins FS, Laakso M, Dermitzakis ET, Boehnke M, McCarthy MI, Wareham NJ, Groop L, Pattou F, Gloyn AL, Dedoussis GV, Lyssenko V, Meigs JB, Barroso I, Watanabe RM, Ingelsson E, Langenberg C, Hamsten A, Florez JC: Genome-wide association identifies nine common variants associated with fasting proinsulin levels and provides new insights into the pathophysiology of type 2 diabetes. Diabetes 2011;60:2624-2634

42. Manning AK, Hivert M-F, Scott RA, Grimsby JL, Bouatia-Naji N, Chen H, Rybin D, Liu C-T, Bielak LF, Amin N, Barnes D, Cadby G, Hottenga J-J, Ingelsson E, Jackson AU, Johnson T, Kanoni S, Ladenvall C, Lagou V, Lahti J, Lecoeur C, Liu Y, Martinez-Larrad MT, Montasser ME, Navarro P, Perry JRB, Rasmussen-Torvik LJ, Salo P, Sattar N, Shungin D, Strawbridge RJ, Tanaka T, van Duijn CM, An P, de Andrade M, Andrews JS, Aspelund T, Atalay M, Aulchenko Y, Balkau B, Bandinelli S, Beckmann JS, Beilby JP, Bellis C, Bergman RN, Blangero J, Boban M, Boehnke M, Boerwinkle E, Bonnycastle LL, Boomsma DI, Borecki IB, Böttcher Y, Bouchard C, Brunner E, Budimir D, Campbell H, Carlson O, Chines PS, Clarke R, Collins FS, Corbatón-Anchuelo A, Couper D, de Faire U, Dedoussis GV, Deloukas P, Dimitriou M, Egan JM, Eiriksdottir G, Erdos MR, Eriksson JG, Eury E, Ferrucci L, Ford I, Forouhi NG, Fox CS, Franzosi MG, Franks PW, Frayling TM, Froguel P, Galan P, de Geus E, Gigante B, Glazer NL, Goel A, Groop L, Gudnason V, Hallmans G, Hamsten A, Hansson O, Harris TB, Hayward C, Heath S, Hercberg S, Hicks AA, Hingorani A, Hofman A, Hui J, Hung J, Jarvelin MR, Jhun MA, Johnson PCD, Jukema JW, Jula A, Kao W, Kaprio J, Kardia SLR, Keinanen-Kiukaanniemi S, Kivimaki M, Kolcic I, Kovacs P, Kumari M, Kuusisto J, Kyvik KO, Laakso M, Lakka T, Lannfelt L, Lathrop GM, Launer LJ, Leander K, Li G, Lind L, Lindstrom J, Lobbens S, Loos RJF, Luan J, Lyssenko V, Mägi R, Magnusson PKE, Marmot M, Meneton P, Mohlke KL, Mooser V, Morken MA, Miljkovic I, Narisu N, O'Connell J, Ong KK, Oostra BA, Palmer LJ, Palotie A, Pankow JS, Peden JF, Pedersen NL, Pehlic M, Peltonen L, Penninx B, Pericic M, Perola M, Perusse L, Peyser PA, Polasek O, Pramstaller PP, Province MA, Räikkönen K, Rauramaa R, Rehnberg E, Rice K, Rotter JI, Rudan I, Ruokonen A, Saaristo T, Sabater-Leal M, Salomaa V, Savage DB, Saxena R, Schwarz P, Seedorf U, Sennblad B, Serrano-Rios M, Shuldiner AR, Sijbrands EJG, Siscovick DS, Smit J, Small KS, Smith NL, Smith AV, Stančáková A, Stirrups K, Stumvoll M, Sun YV, Swift AJ, Tönjes A, Tuomilehto J, Trompet S, Uitterlinden AG, Uusitupa M, Vikström M, Vitart V, Vohl M-C, Voight BF, Vollenweider P, Waeber G, Waterworth DM, Watkins H, Wheeler E, Widen E, Wild SH, Willems SM, Willemsen G, Wilson JF, Witteman JCM, Wright AF, Yaghootkar H, Zelenika D, Zemunik T, Zgaga L, Consortium. tD, Consortium. tM, Wareham NJ, McCarthy MI, Barroso I, Watanabe RM, Florez JC, Dupuis J, Meigs JB, Langenberg C: A genome-wide approach accounting for body mass index identifies genetic variants influencing fasting glycemic traits and insulin resistance. Nat Genet 2012;(in press)

43. Unoki H, Takahashi A, Kawaguchi T, Hara K, Horikoshi M, Andersen G, Ng DP, Holmkvist J, Borch-Johnsen K, Jorgensen T, Sandbaek A, Lauritzen T, Hansen T, Nurbaya S, Tsunoda T, Kubo M, Babazono T, Hirose H, Hayashi M, Iwamoto Y, Kashiwagi A, Kaku K, Kawamori R, Tai ES, Pedersen O, Kamatani N, Kadowaki T, Kikkawa R, Nakamura Y, Maeda S: SNPs in *KCNQ1* are associated with susceptibility to type 2 diabetes in East Asian and European populations. Nat Genet 2008;40:1098-1102

44. Yasuda K, Miyake K, Horikawa Y, Hara K, Osawa H, Furuta H, Hirota Y, Mori H, Jonsson A, Sato Y, Yamagata K, Hinokio Y, Wang HY, Tanahashi T, Nakamura N, Oka Y, Iwasaki N, Iwamoto Y, Yamada Y, Seino Y, Maegawa H, Kashiwagi A, Takeda J, Maeda E, Shin HD, Cho YM, Park KS, Lee HK, Ng MC, Ma RC, So WY, Chan JC, Lyssenko V, Tuomi T, Nilsson P, Groop L, Kamatani N, Sekine A, Nakamura Y, Yamamoto K, Yoshida T, Tokunaga K, Itakura M, Makino H, Nanjo K, Kadowaki T, Kasuga M: Variants in *KCNQ1* are associated with susceptibility to type 2 diabetes mellitus. Nat Genet 2008;40:1092-1097

45. Kooner JS, Saleheen D, Sim X, Sehmi J, Zhang W, Frossard P, Been LF, Chia K-S, Dimas AS, Hassanali N, Jafar T, Jowett JBM, Li X, Radha V, Rees SD, Takeuchi F, Young R, Aung T, Basit A, Chidambaram M, Das D, Grundberg E, Hedman AK, Hydrie ZI, Islam M, Khor C-C, Kowlessur S, Kristensen MM, Liju S, Lim W-Y, Matthews DR, Liu J, Morris AP, Nica AC, Pinidiyapathirage JM, Prokopenko I, Rasheed A, Samuel M, Shah N, Shera AS, Small KS, Suo C, Wickremasinghe AR, Wong TY, Yang M, Zhang F, Abecasis GR, Barnett AH, Caulfield M, Deloukas P, Frayling TM, Froguel P, Kato N, Katulanda P, Kelly MA, Liang J, Mohan V, Sanghera DK, Scott J, Seielstad M, Zimmet PZ, Elliott P, Teo YY, McCarthy MI, Danesh J, Tai ES, Chambers JC: Genome-wide association study in individuals of South Asian ancestry identifies six new type 2 diabetes susceptibility loci. Nat Genet 2011;43:984-989

46. Yamauchi T, Hara K, Maeda S, Yasuda K, Takahashi A, Horikoshi M, Nakamura M, Fujita H, Grarup N, Cauchi S, Ng DP, Ma RC, Tsunoda T, Kubo M, Watada H, Maegawa H, Okada-Iwabu M, Iwabu M, Shojima N, Shin HD, Andersen G, Witte DR, Jorgensen T, Lauritzen T, Sandbaek A, Hansen T, Ohshige T, Omori S, Saito I, Kaku K, Hirose H, So WY, Beury D, Chan JC, Park KS, Tai ES, Ito C, Tanaka Y, Kashiwagi A, Kawamori R, Kasuga M, Froguel P, Pedersen O, Kamatani N, Nakamura Y, Kadowaki T: A genome-wide association study in the Japanese population identifies susceptibility loci for type 2 diabetes at *UBE2E2* and *C2CD4A-C2CD4B*. Nat Genet 2010;42:864-868

47. Cho YS, Chen C-H, Hu C, Long J, Hee Ong RT, Sim X, Takeuchi F, Wu Y, Go MJ, Yamauchi T, Chang Y-C, Kwak SH, Ma RCW, Yamamoto K, Adair LS, Aung T, Cai Q, Chang L-C, Chen Y-T, Gao Y, Hu FB, Kim H-L, Kim S, Kim YJ, Lee JJ-M, Lee NR, Li Y, Liu JJ, Lu W, Nakamura J, Nakashima E, Ng DP-K, Tay WT, Tsai F-J, Wong TY, Yokota M, Zheng W, Zhang R, Wang C, So WY, Ohnaka K, Ikegami H, Hara K, Cho YM, Cho NH, Chang T-J, Bao Y, Hedman AK, Morris AP, McCarthy MI, Takayanagi R, Park KS, Jia W, Chuang L-M, Chan JCN, Maeda S, Kadowaki T, Lee J-Y, Wu J-Y, Teo YY, Tai ES, Shu XO, Mohlke KL, Kato N, Han B-G, Seielstad M: Meta-analysis of genome-wide association studies identifies eight new loci for type 2 diabetes in east Asians. Nat Genet 2012;44:67-72

48. Saxena R, Elbers CC, Guo Y, Peter I, Gaunt TR, Mega JL, Lanktree MB, Tare A, Castillo BA, Li YR, Johnson T, Bruinenberg M, Gilbert-Diamond D, Rajagopalan R, Voight BF, Balasubramanyam A, Barnard J, Bauer F, Baumert J, Bhangale T, Bohm BO, Braund PS, Burton PR, Chandrupatla HR, Clarke R, Cooper-DeHoff RM, Crook ED, Davey-Smith G, Day IN, de Boer A, de Groot MC, Drenos F, Ferguson J, Fox CS, Furlong CE, Gibson Q, Gieger C, Gilhuijs-Pederson LA, Glessner JT, Goel A, Gong Y, Grant SF, Grobbee DE, Hastie C, Humphries SE, Kim CE, Kivimaki M, Kleber M, Meisinger C, Kumari M, Langaee TY, Lawlor DA, Li M, Lobmeyer MT, Maitland-van der Zee AH, Meijs MF, Molony CM, Morrow DA, Murugesan G, Musani SK, Nelson CP, Newhouse SJ, O'Connell JR, Padmanabhan S, Palmen J, Patel SR, Pepine CJ, Pettinger M, Price TS, Rafelt S, Ranchalis J, Rasheed A, Rosenthal E, Ruczinski I, Shah S, Shen H, Silbernagel G, Smith EN, Spijkerman AW, Stanton A, Steffes MW, Thorand B, Trip M, van der Harst P, van der AD, van Iperen EP, van Setten J, van Vliet-Ostaptchouk JV, Verweij N, Wolffenbuttel BH, Young T, Zafarmand MH, Zmuda JM, Boehnke M, Altshuler D, McCarthy M, Kao WH, Pankow JS, Cappola TP, Sever P, Poulter N, Caulfield M, Dominiczak A, Shields DC, Bhatt DL, Zhang L, Curtis SP, Danesh J, Casas JP, van der Schouw YT, Onland-Moret NC, Doevendans PA, Dorn GW, 2nd, Farrall M, FitzGerald GA, Hamsten A, Hegele R, Hingorani AD, Hofker MH, Huggins GS, Illig T, Jarvik GP, Johnson JA, Klungel OH, Knowler WC, Koenig W, Marz W, Meigs JB, Melander O, Munroe PB, Mitchell BD, Bielinski SJ, Rader DJ, Reilly MP, Rich SS, Rotter JI, Saleheen D, Samani NJ, Schadt EE, Shuldiner AR, Silverstein R, Kottke-Marchant K, Talmud PJ, Watkins H, Asselbergs FW, de Bakker PI, McCaffery J, Wijmenga C, Sabatine MS, Wilson JG, Reiner A, Bowden DW, Hakonarson H, Siscovick DS, Keating BJ: Large-scale gene-centric meta-analysis across 39 studies identifies type 2 diabetes loci. American journal of human genetics 2012;90:410-425

49. Morris AP, Voight BF, Teslovich TM, Ferreira T, Segre AV, Steinthorsdottir V, Strawbridge RJ, Khan H, Grallert H, Mahajan A, Prokopenko I, Kang HM, Dina C, Esko T, Fraser RM, Kanoni S, Kumar A, Lagou V, Langenberg C, Luan J, Lindgren CM, Muller-Nurasyid M, Pechlivanis S, Rayner NW, Scott LJ, Wiltshire S, Yengo L, Kinnunen L, Rossin EJ, Raychaudhuri S, Johnson AD, Dimas AS, Loos RJ, Vedantam S, Chen H, Florez JC, Fox C, Liu CT, Rybin D, Couper DJ, Kao WH, Li M, Cornelis MC, Kraft P, Sun Q, van Dam RM, Stringham HM, Chines PS, Fischer K, Fontanillas P, Holmen OL, Hunt SE, Jackson AU, Kong A, Lawrence R, Meyer J, Perry JR, Platou CG, Potter S, Rehnberg E, Robertson N, Sivapalaratnam S, Stancakova A, Stirrups K, Thorleifsson G, Tikkanen E, Wood AR, Almgren P, Atalay M, Benediktsson R, Bonnycastle LL, Burtt N, Carey J, Charpentier G, Crenshaw AT, Doney AS, Dorkhan M, Edkins S, Emilsson V, Eury E, Forsen T, Gertow K, Gigante B, Grant GB, Groves CJ, Guiducci C, Herder C, Hreidarsson AB, Hui J, James A, Jonsson A, Rathmann W, Klopp N, Kravic J, Krjutskov K, Langford C, Leander K, Lindholm E, Lobbens S, Mannisto S, Mirza G, Muhleisen TW, Musk B, Parkin M, Rallidis L, Saramies J, Sennblad B, Shah S, Sigurethsson G, Silveira A, Steinbach G, Thorand B, Trakalo J, Veglia F, Wennauer R, Winckler W, Zabaneh D, Campbell H, van Duijn C, Uitterlinden AG, Hofman A, Sijbrands E, Abecasis GR, Owen KR, Zeggini E, Trip MD, Forouhi NG, Syvanen AC, Eriksson JG, Peltonen L, Nothen MM, Balkau B, Palmer CN, Lyssenko V, Tuomi T, Isomaa B, Hunter DJ, Qi L, Shuldiner AR, Roden M, Barroso I, Wilsgaard T, Beilby J, Hovingh K, Price JF, Wilson JF, Rauramaa R, Lakka TA, Lind L, Dedoussis G, Njolstad I, Pedersen NL, Khaw KT, Wareham NJ, Keinanen-Kiukaanniemi SM, Saaristo TE, Korpi-Hyovalti E, Saltevo J, Laakso M, Kuusisto J, Metspalu A, Collins FS, Mohlke KL, Bergman RN, Tuomilehto J, Boehm BO, Gieger C, Hveem K, Cauchi S, Froguel P, Baldassarre D, Tremoli E, Humphries SE, Saleheen D, Danesh J, Ingelsson E, Ripatti S, Salomaa V, Erbel R, Jockel KH, Moebus S, Peters A, Illig T, de Faire U, Hamsten A, Morris AD, Donnelly PJ, Frayling TM, Hattersley AT, Boerwinkle E, Melander O, Kathiresan S, Nilsson PM, Deloukas P, Thorsteinsdottir U, Groop LC, Stefansson K, Hu F, Pankow JS, Dupuis J, Meigs JB, Altshuler D, Boehnke M, McCarthy MI: Large-scale association analysis provides insights into the genetic architecture and pathophysiology of type 2 diabetes. Nat Genet 2012;

50. Scott RA, Lagou V, Welch RP, Wheeler E, Montasser ME, Luan Ja, Magi R, Strawbridge RJ, Rehnberg E, Gustafsson S, Kanoni S, Rasmussen-Torvik LJ, Yengo L, Lecoeur C, Shungin D, Sanna S, Sidore C, Johnson PCD, Jukema JW, Johnson T, Mahajan A, Verweij N, Thorleifsson G, Hottenga J-J, Shah S, Smith AV, Sennblad B, Gieger C, Salo P, Perola M, Timpson NJ, Evans DM, Pourcain BS, Wu Y, Andrews JS, Hui J, Bielak LF, Zhao W, Horikoshi M, Navarro P, Isaacs A, O'Connell JR, Stirrups K, Vitart V, Hayward C, Esko T, Mihailov E, Fraser RM, Fall T, Voight BF, Raychaudhuri S, Chen H, Lindgren CM, Morris AP, Rayner NW, Robertson N, Rybin D, Liu C-T, Beckmann JS, Willems SM, Chines PS, Jackson AU, Kang HM, Stringham HM, Song K, Tanaka T, Peden JF, Goel A, Hicks AA, An P, Muller-Nurasyid M, Franco-Cereceda A, Folkersen L, Marullo L, Jansen H, Oldehinkel AJ, Bruinenberg M, Pankow JS, North KE, Forouhi NG, Loos RJF, Edkins S, Varga TV, Hallmans G, Oksa H, Antonella M, Nagaraja R, Trompet S, Ford I, Bakker SJL, Kong A, Kumari M, Gigante B, Herder C, Munroe PB, Caulfield M, Antti J, Mangino M, Small K, Miljkovic I, Liu Y, Atalay M, Kiess W, James AL, Rivadeneira F, Uitterlinden AG, Palmer CNA, Doney ASF, Willemsen G, Smit JH, Campbell S, Polasek O, Bonnycastle LL, Hercberg S, Dimitriou M, Bolton JL, Fowkes GR, Kovacs P, Lindstrom J, Zemunik T, Bandinelli S, Wild SH, Basart HV, Rathmann W, Grallert H, Maerz W, Kleber ME, Boehm BO, Peters A, Pramstaller PP, Province MA, Borecki IB, Hastie ND, Rudan I, Campbell H, Watkins H, Farrall M, Stumvoll M, Ferrucci L, Waterworth DM, Bergman RN, Collins FS, Tuomilehto J, Watanabe RM, de Geus EJC, Penninx BW, Hofman A, Oostra BA, Psaty BM, Vollenweider P, Wilson JF, Wright AF, Hovingh GK, Metspalu A, Uusitupa M, Magnusson PKE, Kyvik KO, Kaprio J, Price JF, Dedoussis GV, Deloukas P, Meneton P, Lind L, Boehnke M, Shuldiner AR, van Duijn CM, Morris AD, Toenjes A, Peyser PA, Beilby JP, Korner A, Kuusisto J, Laakso M, Bornstein SR, Schwarz PEH, Lakka TA, Rauramaa R, Adair LS, Smith GD, Spector TD, Illig T, de Faire U, Hamsten A, Gudnason V, Kivimaki M, Hingorani A, Keinanen-Kiukaanniemi SM, Saaristo TE, Boomsma DI, Stefansson K, van der Harst P, Dupuis J, Pedersen NL, Sattar N, Harris TB, Cucca F, Ripatti S, Salomaa V, Mohlke KL, Balkau B, Froguel P, Pouta A, Jarvelin M-R, Wareham NJ, Bouatia-Naji N, McCarthy MI, Franks PW, Meigs JB, Teslovich TM, Florez JC, Langenberg C, Ingelsson E, Prokopenko I, Barroso I: Large-scale association analyses identify new loci influencing glycemic traits and provide insight into the underlying biological pathways. Nat Genet 2012;44:991-1005

51. Suter SL, Nolan JJ, Wallace P, Gumbiner B, Olefsky JM: Metabolic effects of new oral hypoglycemic agent CS-045 in NIDDM subjects. Diabetes Care 1992;15:193-203

52. Nolan JJ, Ludvik B, Beerdsen P, Joyce M, Olefsky J: Improvement in glucose tolerance and insulin resistance in obese subjects treated with troglitazone. N Engl J Med 1994;331:1188-1193

53. Lebovitz HE, Dole JF, Patwardhan R, Rappaport EB, Freed MI: Rosiglitazone monotherapy is effective in patients with type 2 diabetes. J Clin Endocrinol Metab 2001;86:280-288

54. Buchanan TA, Xiang AH, Peters RK, Kjos SL, Marroquin A, Goico J, Ochoa C, Tan S, Berkowitz K, Hodis HN, Azen SP: Preservation of pancreatic β-cell function and prevention of type 2 diabetes by pharmacological treatment of insulin resistance in high-risk Hispanic women. Diabetes 2002;51:2796-2803

55. Kirchheiner J, Brockm-Iller J, Meineke I, Bauer S, Rohde W, Meisel C, Roots I: Impact of CYP2C9 amino acid polymorphisms on glyburide kinetics and on the insulin and glucose response in healthy volunteers. Clin Pharmacol Ther 2002;71:286-296

56. Niemi M, Cascorbi I, Timm R, Kroemer HK, Neuvonen PJ, Kivistö KT: Glyburide and glimepiride pharmacokinetics in subjects with different CYP2C9 genotypes. Clin Pharmacol Ther 2002;72:326-332

57. Wolford JK, Yeatts KA, Dhanjal SK, Black MH, Xiang AH, Buchanan TA, Watanabe RM: Sequence variation in *PPARG* may underlie differential response to troglitazone. Diabetes 2005;54:3319-3325

58. Sesti G, Laratta E, Cardellini M, Andreozzi F, Del Guerra S, Irace C, Gnasso A, Grupillo M, Lauro R, Hribal ML, Perticone F, Marchetti P: The E23K variant of *KCNJ11* encoding the pancreatic β-cell KATP channel subunits Kir6.2 is associated with an increased risk of secondary failure to sulfonylurea in patients with type 2 diabetes. J Clin Endocrinol Metab 2006;online

59. Lander ES, Linton LM, Birren B, Nusbaum C, Zody MC, Baldwin J, Devon K, Dewar K, Doyle M, FitzHugh W, Funke R, Gage D, Harris K, Heaford A, Howland J, Kann L, Lehoczky J, LeVine R, McEwan P, McKernan K, Meldrim J, Mesirov JP, Miranda C, Morris W, Naylor J, Raymond C, Rosetti M, Santos R, Sheridan A, Sougnez C, Stange-Thomann N, Stojanovic N, Subramanian A, Wyman D, Rogers J, Sulston J, Ainscough R, Beck S, Bentley D, Burton J, Clee C, Carter N, Coulson A, Deadman R, Deloukas P, Dunham A, Dunham I, Durbin R, French L, Grafham D, Gregory S, Hubbard T, Humphray S, Hunt A, Jones M, Lloyd C, McMurray A, Matthews L, Mercer S, Milne S, Mullikin JC, Mungall A, Plumb R, Ross M, Shownkeen R, Sims S, Waterston RH, Wilson RK, Hillier LW, McPherson JD, Marra MA, Mardis ER, Fulton LA, Chinwalla AT, Pepin KH, Gish WR, Chissoe SL, Wendl MC, Delehaunty KD, Miner TL, Delehaunty A, Kramer JB, Cook LL, Fulton RS, Johnson DL, Minx PJ, Clifton SW, Hawkins T, Branscomb E, Predki P, Richardson P, Wenning S, Slezak T, Doggett N, Cheng JF, Olsen A, Lucas S, Elkin C, Uberbacher E, Frazier M, Gibbs RA, Muzny DM, Scherer SE, Bouck JB, Sodergren EJ, Worley KC, Rives CM, Gorrell JH, Metzker ML, Naylor SL, Kucherlapati RS, Nelson DL, Weinstock GM, Sakaki Y, Fujiyama A, Hattori M, Yada T, Toyoda A, Itoh T, Kawagoe C, Watanabe H, Totoki Y, Taylor T, Weissenbach J, Heilig R, Saurin W, Artiguenave F, Brottier P, Bruls T, Pelletier E, Robert C, Wincker P, Smith DR, Doucette-Stamm L, Rubenfield M, Weinstock K, Lee HM, Dubois J, Rosenthal A, Platzer M, Nyakatura G, Taudien S, Rump A, Yang H, Yu J, Wang J, Huang G, Gu J, Hood L, Rowen L, Madan A, Qin S, Davis RW, Federspiel NA, Abola AP, Proctor MJ, Myers RM, Schmutz J, Dickson M, Grimwood J, Cox DR, Olson MV, Kaul R, Shimizu N, Kawasaki K, Minoshima S, Evans GA, Athanasiou M, Schultz R, Roe BA, Chen F, Pan H, Ramser J, Lehrach H, Reinhardt R, McCombie WR, de la Bastide M, Dedhia N, Blocker H, Hornischer K, Nordsiek G, Agarwala R, Aravind L, Bailey JA, Bateman A, Batzoglou S, Birney E, Bork P, Brown DG, Burge CB, Cerutti L, Chen HC, Church D, Clamp M, Copley RR, Doerks T, Eddy SR, Eichler EE, Furey TS, Galagan J, Gilbert JG, Harmon C, Hayashizaki Y, Haussler D, Hermjakob H, Hokamp K, Jang W, Johnson LS, Jones TA, Kasif S, Kaspryzk A, Kennedy S, Kent WJ, Kitts P, Koonin EV, Korf I, Kulp D, Lancet D, Lowe TM, McLysaght A, Mikkelsen T, Moran JV, Mulder N, Pollara VJ, Ponting CP, Schuler G, Schultz J, Slater G, Smit AF, Stupka E, Szustakowki J, Thierry-Mieg D, Wagner L, Wallis J, Wheeler R, Williams A, Wolf YI, Wolfe KH, Yang SP, Yeh RF, Collins F, Guyer MS, Peterson J, Felsenfeld A, Wetterstrand KA, Patrinos A, Morgan MJ: Initial sequencing and analysis of the human genome. Nature 2001;409:860-921

60. Venter JC, Adams MD, Myers EW, Li PW, Mural RJ, Sutton GG, Smith HO, Yandell M, Evans CA, Holt RA, Gocayne JD, Amanatides P, Ballew RM, Huson DH, Wortman JR, Zhang Q, Kodira CD, Zheng XH, Chen L, Skupski M, Subramanian G, Thomas PD, Zhang J, Gabor Miklos GL, Nelson C, Broder S, Clark AG, Nadeau J, McKusick VA, Zinder N, Levine AJ, Roberts RJ, Simon M, Slayman C, Hunkapiller M, Bolanos R, Delcher A, Dew I, Fasulo D, Flanigan M, Florea L, Halpern A, Hannenhalli S, Kravitz S, Levy S, Mobarry C, Reinert K, Remington K, Abu-Threideh J, Beasley E, Biddick K, Bonazzi V, Brandon R, Cargill M, Chandramouliswaran I, Charlab R, Chaturvedi K, Deng Z, Di Francesco V, Dunn P, Eilbeck K, Evangelista C, Gabrielian AE, Gan W, Ge W, Gong F, Gu Z, Guan P, Heiman TJ, Higgins ME, Ji RR, Ke Z, Ketchum KA, Lai Z, Lei Y, Li Z, Li J, Liang Y, Lin X, Lu F, Merkulov GV, Milshina N, Moore HM, Naik AK, Narayan VA, Neelam B, Nusskern D, Rusch DB, Salzberg S, Shao W, Shue B, Sun J, Wang Z, Wang A, Wang X, Wang J, Wei M, Wides R, Xiao C, Yan C, Yao A, Ye J, Zhan M, Zhang W, Zhang H, Zhao Q, Zheng L, Zhong F, Zhong W, Zhu S, Zhao S, Gilbert D, Baumhueter S, Spier G, Carter C, Cravchik A, Woodage T, Ali F, An H, Awe A, Baldwin D, Baden H, Barnstead M, Barrow I, Beeson K, Busam D, Carver A, Center A, Cheng ML, Curry L, Danaher S, Davenport L, Desilets R, Dietz S, Dodson K, Doup L, Ferriera S, Garg N, Gluecksmann A, Hart B, Haynes J, Haynes C, Heiner C, Hladun S, Hostin D, Houck J, Howland T, Ibegwam C, Johnson J, Kalush F, Kline L, Koduru S, Love A, Mann F, May D, McCawley S, McIntosh T, McMullen I, Moy M, Moy L, Murphy B, Nelson K, Pfannkoch C, Pratts E, Puri V, Qureshi H, Reardon M, Rodriguez R, Rogers YH, Romblad D, Ruhfel B, Scott R, Sitter C, Smallwood M, Stewart E, Strong R, Suh E, Thomas R, Tint NN, Tse S, Vech C, Wang G, Wetter J, Williams S, Williams M, Windsor S, Winn-Deen E, Wolfe K, Zaveri J, Zaveri K, Abril JF, Guigo R, Campbell MJ, Sjolander KV, Karlak B, Kejariwal A, Mi H, Lazareva B, Hatton T, Narechania A, Diemer K, Muruganujan A, Guo N, Sato S, Bafna V, Istrail S, Lippert R, Schwartz R, Walenz B, Yooseph S, Allen D, Basu A, Baxendale J, Blick L, Caminha M, Carnes-Stine J, Caulk P, Chiang YH, Coyne M, Dahlke C, Mays A, Dombroski M, Donnelly M, Ely D, Esparham S, Fosler C, Gire H, Glanowski S, Glasser K, Glodek A, Gorokhov M, Graham K, Gropman B, Harris M, Heil J, Henderson S, Hoover J, Jennings D, Jordan C, Jordan J, Kasha J, Kagan L, Kraft C, Levitsky A, Lewis M, Liu X, Lopez J, Ma D, Majoros W, McDaniel J, Murphy S, Newman M, Nguyen T, Nguyen N, Nodell M, Pan S, Peck J, Peterson M, Rowe W, Sanders R, Scott J, Simpson M, Smith T, Sprague A, Stockwell T, Turner R, Venter E, Wang M, Wen M, Wu D, Wu M, Xia A, Zandieh A, Zhu X: The sequence of the human genome. Science 2001;291:1304-1351.

61. Altshuler D, Brooks LD, Chakravarti A, Collins FS, Daly MJ, Donnelly P, Consortium TIH: A haplotype map of the human genome. Nature 2005;437:1299-1320

62. Guttmacher AE, Collins FS: Realizing the promise of genomics in biomedical research. JAMA 2005;294:1399-1402

63. Marreiro DN, Geloneze B, Tambascia MA, Lerario AC, Halpern A, Cozzolino SM: Effect of zinc supplementation on serum leptin levels and insulin resistance of obese women. Biol Trace Elem Res 2006;112:109-118

64. Singh RB, Niaz MA, Rastogi SS, Bajaj S, Gaoli Z, Shoumin Z: Current zinc intake and risk of diabetes and coronary artery disease and factors associated with insulin resistance in rural and urban populations of North India. J Am Coll Nutr 1998;17:564-570

65. Maestro B, Molero S, Bajo S, Davila N, Calle C: Transcriptional activation of the human insulin receptor gene by 1,25-dihydroxyvitamin D(3). Cell Biochem Funct 2002;20:227-232

66. Scragg R, Sowers M, Bell C: Serum 25-hydroxyvitamin D, diabetes, and ethnicity in the Third National Health and Nutrition Examination Survey. Diabetes Care 2004;27:2813-2818

67. Pittas AG, Dawson-Hughes B, Li T, Van Dam RM, Willett WC, Manson JE, Hu FB: Vitamin D and calcium intake in relation to type 2 diabetes in women. Diabetes Care 2006;29:650-656

68. Pittas AG, Harris SS, Stark PC, Dawson-Hughes B: The effects of calcium and vitamin D supplementation on blood glucose and markers of inflammation in nondiabetic adults. Diabetes Care 2007;30:980-986

69. Florez JC, Agapakis CM, Burtt NP, Sun M, Almgren P, Rastam L, Tuomi T, Gaudet D, Hudson TJ, Daly MJ, Ardlie KG, Hirschhorn JN, Groop L, Altshuler D: Association testing of the protein tyrosine phosphatase 1B gene (*PTPN1*) with type 2 diabetes in 7,883 people. Diabetes 2005;54:1884-1891

70. Florez JC, Wiltshire S, Agapakis CM, Burtt NP, de Bakker PIW, Almgren P, Bengtsson Bostrom K, Tuomi T, Gaudet D, Daly MJ, Hirschhorn JN, McCarthy MI, Altshuler D, Groop L: High-density haplotype structure and association testing of the insulin-degrading enzyme (*IDE*) gene with type 2 diabetes in 4,206 people. Diabetes 2006;55:128-135

71. Sun MW, Lee JY, de Bakker PIW, Burtt NP, Almgren P, Rastam L, Tuomi T, Gaudet D, Daly MJ, Hirschhorn JN, Altshuler D, Groop L, Florez JC: Haplotype structures and large-scale association testing of the 5' AMP-activated protein kinase genes *PRKAA2*, *PRKAB1*, and *PRKAB2* with type 2 diabetes. Diabetes 2006;55:849-855

72. Saxena R, Gianniny L, Burtt NP, Lyssenko V, Giuducci C, Sjogren M, Florez JC, Almgren P, Isomaa B, Orho-Melander M, Lindblad U, Daly MJ, Tuomi T, Hirschhorn JN, Ardlie KG, Groop LC, Altshuler D: Common single nucleotide polymorphisms in *TCF7L2* are reproducibly associated with type 2 diabetes and reduce the insulin response to glucose in nondiabetic individuals. Diabetes 2006;55:2890-2895

73. Florez JC, Sjögren M, Burtt N, Orho-Melander M, Schayer S, Sun M, Almgren P, Lindblad U, Tuomi T, Gaudet D, Hudson TJ, Daly MJ, Ardlie KG, Hirschhorn JN, Altshuler D, Groop L: Association testing in 9,000 people fails to confirm the association of the insulin receptor substrate-1 G972R polymorphism with type 2 diabetes. Diabetes 2004;53:3313-3318

74. Lyon HN, Florez JC, Bersaglieri T, Saxena R, Winckler W, Almgren P, Lindblad U, Tuomi T, Gaudet D, Zhu X, Cooper R, Ardlie KG, Daly MJ, Altshuler D, Groop L, Hirschhorn JN: Common variants in the *ENPP1* gene are not reproducibly associated with diabetes or obesity. Diabetes 2006;55:3180-3184

75. Florez JC, Jablonski KA, Bayley N, Pollin TI, de Bakker PIW, Shuldiner AR, Knowler WC, Nathan DM, Altshuler D, for the Diabetes Prevention Program Research G: *TCF7L2* polymorphisms and progression to diabetes in the Diabetes Prevention Program. N Engl J Med 2006;355:241-250

76. Florez JC, Jablonski KA, Kahn SE, Franks PW, Dabelea D, Hamman RF, Knowler WC, Nathan DM, Altshuler D, for the Diabetes Prevention Program Research G: Type 2 diabetes-associated missense polymorphisms *KCNJ11* E23K and *ABCC8* A1369S influence progression to diabetes and response to interventions in the Diabetes Prevention Program. Diabetes 2007;56:531-536

77. Kieffer TJ, Habener JF: The glucagon-like peptides. Endocr Rev 1999;20:876-913

78. DPPRG: Reduction in the incidence of type 2 diabetes with lifestyle intervention or metformin. N Engl J Med 2002;346:393-403

79. Lyssenko V, Almgren P, Anevski D, Perfekt R, Lahti K, Nissen M, Isomaa B, Forsen B, Homstrom N, Saloranta C, Taskinen M-R, Groop L, Tuomi T, for the Botnia Study Group: Predictors of and longitudinal changes in insulin sensitivity and secretion preceding onset of type 2 diabetes. Diabetes 2005;54:166-174

80. Smirnakis KV, Chasan-Taber L, Wolf M, Markenson G, Ecker JL, Thadhani R: Postpartum diabetes screening in women with a history of gestational diabetes. Obstet Gynecol 2005;106:1297-1303

81. Levey AS, Bosch JP, Lewis JB, Greene T, Rogers N, Roth D, for the Modification of Diet in Renal Disease Study Group*: A more accurate method to estimate glomerular filtration rate from serum creatinine: a new prediction equation. Ann Intern Med 1999;130:461-470

82. Tang K, Fu DJ, Julien D, Braun A, Cantor CR, Koster H: Chip-based genotyping by mass spectrometry. Proc Natl Acad Sci U S A 1999;96:10016-10020

83. Egan JM, Meneilly GS, Habener JF, Elahi D: Glucagon-like peptide-1 augments insulin-mediated glucose uptake in the obese state. J Clin Endocrinol Metab 2002;87:3768-3773

84. Matthews DR, Hosker JP, Rudenski AS, Naylor BA, Treacher DF, Turner RC: Homeostasis model assessment: insulin resistance and beta-cell function from fasting plasma glucose and insulin concentrations in man. Diabetologia 1985;28:412-419
