# Supplementary material for: The Study to Understand the Genetics of the Acute Response to Metformin and Glipizide in Humans (SUGAR-MGH): Design of a pharmacogenetic Resource for Type 2 Diabetes
Source: PLoS One. 2015 Mar 26;10(3):e0121553. doi: 10.1371/journal.pone.0121553 (PMC4374872; doi:10.1371/journal.pone.0121553)
Supplement: S1 Table — Single nucleotide polymorphisms (SNPs) previously associated with fasting glucose were used to calculate the fasting glucose genetic risk score (GRS); SNPs previously associated with fasting insulin were used to calculated the fasting insulin GRS. For each SNP, an effect allele, which raises fasting glucose or fasting insulin, is provided. (DOCX) [file pone.0121553.s016.docx]

**S1 Table.** Investigated genetic markers

| SNP | Chromosome | Nearest gene | Effect allele/Other allele |
| --- | --- | --- | --- |
| **SNPs associated with fasting glucose** | | |  |
| rs11708067 | 3 | *ADCY5* | *A/G* |
| rs10885122 | 10 | *ADRAA2* | *G/T* |
| rs11715915 | 3 | *AMT* | *C/T* |
| rs11071657 | 15 | *C2CD4B* | *A/G* |
| rs9368222 | 6 | *CDKAL1* | *A/C* |
| rs10811661 | 9 | *CDKN2A/B* | *T/C* |
| rs11605924 | 11 | *CRY2* | *A/C* |
| rs2191349 | 7 | *DGKB/TMEM195* | *T/G* |
| rs1371614 | 2 | *DPYSL5* | *T/C* |
| rs174550 | 11 | *FADS1* | *T/C* |
| rs6113722 | 20 | *FOXA2* | *G/A* |
| rs560887 | 2 | *G6PC2* | *C/T* |
| rs4607517 | 7 | *GCK* | *A/G* |
| rs1260326 | 2 | *GCKR* | *C/T* |
| rs2302593 | 19 | *GIPR* | *C/G* |
| rs7034200 | 9 | *GLIS3* | *A/C* |
| rs6943153 | 7 | *GRB10* | *T/C* |
| rs4402960 | 3 | *IGF2BP2* | *T/G* |
| rs576674 | 13 | *KL* | *G/A* |
| rs7944584 | 11 | *MADD* | *A/T* |
| rs10830963 | 11 | *MTNR1B* | *G/C* |
| rs1483121 | 11 | *OR4S1* | *G/A* |
| rs10747083 | 12 | *P2RX2* | *A/G* |
| rs6235 | 5 | *PCSK1* | *C/G* |
| rs2293941 | 13 | *PDX1* | *A/G* |
| rs4841132 | 8 | *PPP1R3B* | *A/G* |
| rs11920090 | 3 | *SLC2A2* | *T/A* |
| rs13266634 | 8 | *SLC30A8* | *C/T* |
| rs7903146 | 10 | *TCF7L2* | *T/C* |
| rs6072275 | 20 | *TOP1* | *A/G* |
| rs4502156 | 15 | *VSP13C/C2CD4A/B* | *T/C* |
| rs3783347 | 14 | *WARS* | *G/T* |
| rs4457053 | 5 | *ZBED3* | *G/A* |
| rs17762454 | 6 | *RREB1* | *T/C* |
| **SNPs associated with fasting insulin** | | |  |
| rs7607980 | 2 | *COBLL1-GRB14* | *T/C* |
| rs9939609 | 16 | *FTO* | *A/T* |
| rs1260326 | 2 | *GCKR* | *C/T* |
| rs10195252 | 2 | *GRB14* | *T/C* |
| rs1167800 | 7 | *HIP1* | *A/G* |
| rs35767 | 12 | *IGF1* | *G/A* |
| rs7578326 | 2 | *IRS1* | *A/G* |
| rs2820436 | 1 | *LYPLAL1* | *C/A* |
| rs4691380 | 4 | *PDGFC* | *C/T* |
| rs4841132 | 8 | *PPP1R3B* | *A/G* |
| rs9884482 | 4 | *TET2* | *C/T* |
| rs731839 | 19 | *PEPD* | *G/A* |
| rs2745353 | 6 | *RSPO3* | *T/C* |
| rs1530559 | 2 | *YSK4* | *A/G* |
| SNP: single nucleotide polymorphism | | | |
